# Reporting quality of randomized controlled trials of angina pectoris with integrated traditional Chinese and western medicine interventions: a cross-sectional study

**DOI:** 10.1186/s12874-023-01953-1

**Published:** 2023-05-23

**Authors:** Jiashuai Deng, Juan He, Juan Wang, Chung Wah Cheng, Yalin Jiao, Nana Wang, Ji Li, Ping Wang, Fei Han, Aiping Lyu, Zhaoxiang Bian, Xuan Zhang

**Affiliations:** 1 School of Traditional Chinese Medicine, Beijing University of Chinese Medicine, Beijing, China; 2 Chinese EQUATOR Centre, Chinese Clinical Trial Registry (Hong Kong), Hong Kong Chinese Medicine Clinical Study Centre, School of Chinese Medicine, Hong Kong Baptist University, Hong Kong SAR, China; 3 Department of Pediatrics, Guang’anmen Hospital, China Academy of Chinese Medical Sciences, Beijing, China; 4 Xiyuan Hospital, China Academy of Chinese Medical Sciences, Beijing, China; 5 Centre for Chinese Herbal Medicine Drug Development Limited, Hong Kong Baptist University, Hong Kong SAR, China

**Keywords:** Angina pectoris (AP), CONSORT guideline, Randomized controlled trials (RCTs), Reporting quality, Integrated traditional Chinese and western medicine (ITCWM)

## Abstract

**Background and objective:**

Integrated traditional Chinese and western medicine (ITCWM), as a representative type of complex intervention, is commonly used for the treatment of angina pectoris (AP) in clinical practice. However, it is unclear whether the details of ITCWM interventions, such as rationale for selection and design, implementation and potential interactions for different therapies, were adequately reported. Therefore, this study aimed to describe the reporting characteristics and quality in randomized controlled trials (RCTs) of AP with ITCWM interventions.

**Methods:**

Through a search of 7 electronic databases, we identified RCTs of AP with ITCWM interventions published in both English and Chinese from 1^st^ Jan 2017 to 6^th^ Aug 2022. The general characteristics of included studies were summarized, further, the quality of reporting was assessed based on three Checklists, including the CONSORT with 36 items (except for one item 1b about abstract), the CONSORT for abstracts (17 items), and a self-designed ITCWM-related checklist (21 items covering rationale and details of interventions, outcome assessment and analysis). The quality of RCTs published in English and Chinese, as well as journals and dissertations were also compared.

**Results:**

A total of 451 eligible RCTs were included. For the reporting compliance, the mean score (95% Confidence Interval) of the CONSORT (72 scores in total), CONSORT for abstract (34 scores in total), and ITCWM-related (42 scores in total) checklists was 27.82 (27.44–28.19), 14.17 (13.98–14.37) and 21.06 (20.69–21.43), respectively. More than half items were evaluated as poor quality (reporting rate < 50%) among each Checklist. Moreover, the reporting quality of publications in English journals was higher than that in Chinese journals in terms of the CONSORT items. The reporting of published dissertations was better than that in journal publications regarding both the CONSORT and ITCWM-specific items.

**Conclusion:**

Although the CONSORT appears to have enhanced the reporting of RCTs in AP, the quality of ITCWM specifics is variable and in need of improvement. Reporting guideline of the ITCWM recommendations should be developed thus to improve their quality.

**Supplementary Information:**

The online version contains supplementary material available at 10.1186/s12874-023-01953-1.

## Introduction

Angina pectoris (AP), as a type of ischemic heart diseases (IHD), is presented by recurrent chest discomforts (radiate up to the neck or jaw, or down either arm or to the epigastric area) due to physical exertion or emotional distress [1, 2]. In clinical practice, AP usually defined as an early warning signal for various underlying heart diseases, although its symptoms were hidden (such as crescendos over a period of minutes which dissipates with rest or nitroglycerin treatment) [3, 4]. For example, some studies found that patients with stable AP had higher rates of cardiovascular death, myocardial infarction (MI), or stroke, and lower quality of life than those without AP [5, 6]. Currently, the common therapies of AP include pharmacological and invasive treatments, but several adverse effects are unavoidable, including renal failure, hepatotoxicity, bleeding complications, anaphylaxis, and hematologic abnormalities, etc. Furthermore, the medical and surgical expenses in treating AP are expensive [7–9]. Therefore, traditional Chinese medicine (TCM) is more accepted by AP patients as its straightforward effect with few side effects, and inexpensive [10]. Based on previous evidence, TCM interventions could relieve myocardial ischemia, reduce myocardial oxygen consumption, exert antiplatelet and anticoagulant activity, and relieve pain [11].

In China, most patients preferred integrated traditional Chinese and western medicine (ITCWM) over TCM or WM alone [12–15]. Scholars have identified the promising therapeutical results of ITCWM interventions for the treatment of AP for decades, after the first ITCWM interventional trial of AP was published in 1994 [16]. To standard the clinical practice, scholars made lots of efforts. Specifically, the first guideline of ITCWM treatment protocol for AP was developed in 2010 [17]. In 2017, an international consensus-based recommendations for personalized treatment protocol of AP was issued [18]. During 2018–2022, several standards or guidelines for AP with ITCWM interventions have been published and updated [19–23]. Consequently, an increasing number of ITCWM interventional randomized controlled trials (RCTs) for AP was registered and conducted, but the quality of these RCTs is variable. Previous systematic reviews (SRs) with meta-analyses (MAs) indicated that there is a large room for improvements of the included RCTs, particular in methodological quality [24–30]. Appraisal of the trial quality largely depends on the reporting level of study design, implementation, and analysis. Although poor reporting does not necessarily mean poor methodology or trial quality, it is an essential indicator for reviewers and readers to evaluate the reliability and validity of trial findings.

However, no previous study has assessed whether ITCWM-related aspects (e.g., selection rationale, study design, implementation and potential interactions of different therapies) are sufficiently reported in the published RCTs of AP; indeed, no study has identified what key information affecting the quality of research should be described in the reports. Therefore, the present review is designed i) to summarize general characteristics from published RCTs concerning ITCWM for AP; ii) to assess the reporting quality of these trials based on the CONSORT; and iii) to evaluate whether necessary information related to ITCWM design, implementation and analysis is adequately reported based on a self-designed ITCWM checklist.

## Methods

### Eligibility criteria

This study included ITCWM interventional RCTs of AP published in English and Chinese from 1^st^ January 2017 to 6^th^ August 2022. Subjects with a definite diagnosis of AP, regardless of age, gender, type of disease and severity, were included. The ITCWM intervention was defined as a combination of TCM treatments and western medicine (WM) therapies. Specifically, we included a wide range of TCM interventions, such as Chinese herbal medicine, acupuncture and moxibustion. A various form of pharmaceutical or revascularization intervention, such as percutaneous coronary intervention (PCI), external counter pulsation and coronary angioplasty bypass grafting (CABG), were also included in WM interventions. There were no limitations in the types of control groups and the assessed outcomes. Duplicate publications, retracted RCTs, non-randomized or non-controlled trials, quasi-RCTs, non-ITCWM interventional trials, study protocols, reviews, observational studies, case reports, abstracts or non-full-text reports, non-human studies, and non-English/Chinese reports were excluded. Considering the quality of included articles, regarding Chinese publications, we further excluded the publications from Non-Core journals [31, 32]. All data screening, processing and reporting in this review are based on the PRISMA 2020 checklist (Supplementary file 1).

### Search strategy

A systematic search was conducted on 6^th^ August 2022 for the following databases: All EBM Reviews, AMED (Allied and Complementary Medicine), Embase, Ovid MEDLINE, CNKI (China National Knowledge Infrastructure), Wanfang Database and VIP Chinese Medical Journal Database. The search terms included “Angina Pectoris”, “stenocardia”, “randomized controlled trial”, “random”, “Chinese medicine”, “herbal”, and “drug”, etc. The detailed search strategy is presented in Supplementary file 2.

### Study selection

Two authors (JSD and JW) independently screened the titles and abstracts of the retrieved records based on the inclusion and exclusion criteria. Full reports of any potentially relevant papers were reviewed and double-checked for further assessment of eligibility. Endnote X9 was used for screening and processing the articles, which facilitate a double-check for all records. Differences of opinion were settled by consensus or referral to a third author (XZ).

### Data extraction

We developed a data extraction form of general characteristics of included records, including title, publication year and language, information of corresponding author(s), types of publications (e.g., dissertation or journal publication), study design (e.g., assignment and sample size), features of interventions, participants (e.g., types of AP and TCM patterns), duration of treatment and follow-up (if any) and types of outcome(s). Two authors (JSD and JW) independently extracted the data, and another two authors (XZ and NNW) conducted a second check. Disagreements were resolved by discussion.

### Reporting quality assessment

The reporting quality of included studies was evaluated according to the CONSORT 2010 checklist, of which the checklist of the CONSORT for Abstract was independently extracted for separate evaluation. In addition, a special-designed checklist comprised of 21 items related to specific, unique characteristics of ITCWM interventional trials was formulated (Table 1) based on internal discussion across five authors (XZ, JL, PW, FH and ZXB). This checklist focused on the identification of critical issues in the design, implementation and assessment of ITCWM interventions, particular in the selection rationale, details of combination, efficacy assessment, and potential interaction (if applicable). Each item/question was scored in terms of four levels: ① “2” for “fully reported”; ② “1” for “partially reported”; ③ “0” for “not reported”; and ④ “Not applicable”. The details of scoring rules for ITCWM are presented in Supplementary file 3 which includes the explanations for each question and examples of eligible reporting. For rating the CONSORT items, the assessment principles were referred to the CONSORT 2010 statement (including the CONSORT for Abstract guideline) with its explanation and elaboration (E&E) documents [33, 34]. The quality assessment was independently conducted by one author (JSD) and verified by another author (JW). Agreement for inclusion was calculated using the Cohen ‘s kappa (k) coefficient with the following scale: a k of 0.40 or lower, poor; 0.41 to 0.60, moderate; 0.61 to 0.80, substantial; and 0.81 to 1, almost perfect [35]. Possible disagreements were resolved with the consultation of a third senior author (XZ).

**Table 1:** Questions for assessing the reporting of ITCWM-specific items

Item no	Specifics
**Q1**	Whether the feature of ITCWM was presented in Title?
**Q2**	Whether the eligibility criteria of participants included both Chinese and western medical diagnosis in Methods of Abstract?
**Q3**	Whether the study objectives or hypotheses were focused on the ITCWM interventions in Abstract?
**Q4**	Whether the outcome measures included both TCM and WM related endpoints in Abstract?
**Q5**	Whether the effect of studied ITCWM interventions was reported in Conclusion of Abstract?
**Q6**	Whether the feature or design of ITCWM study were reflected in Keywords?
**Q7**	Whether the reason/rationale about ITCWM intervention for the study design was reported in the Background?
**Q8**	Whether the objectives or hypotheses were focused on the ITCWM interventions in the Background?
**Q9**	Whether the eligibility criteria of participants included both Chinese and western medical diagnosis in Methods?
**Q10**	Whether the specific information of disease (e.g., classification of disease, treatment points, stages of diseases) of the ITCWM was reported in Methods?
**Q11**	Whether the specific type/way of integration of TCM and WM interventions (such as overlying, one-after-another, or add-on design) was reported in Methods?
**Q12**	In the ITCWM group, whether TCM intervention(s) was reported with sufficient details to allow replication, including how and when they were administered?
**Q13**	In the ITCWM group, whether WM intervention(s) was reported with sufficient details to allow replication, including how and when they were administered?
**Q14**	In the control group, whether sufficient details were reported to allow replication?
**Q15**	Whether the outcome measures included both TCM and WM related endpoints in Methods?
**Q16**	For the studies with open label, whether any reasons or explanations for such design was reported?
**Q17**	In the control group(s), did the placebo of WM invention(s) was included? If so, whether sufficient details were provided?
**Q18**	In the control group(s), did the placebo of TCM invention(s) was included? If so, whether sufficient details were provided?
**Q19**	In the section of Results, whether any information about the participants exposed to ITCWM treatment prior to recruitment was mentioned in the baseline data?
**Q20**	Whether interpretation and significance of studied ITCWM interventions for the disease was reported in Discussion?
**Q21**	Whether any potential conflicts of interests were clearly reported?

*Abbreviations*: *ITCWM* Integrated traditional Chinese medicine and western medicine, *TCM* Traditional Chinese medicine, *WM* Western medicine

### Data analysis

We applied frequency and percentage to present categorical variables, and median and interquartile range (IQR) to present continuous variables. For individual item of reporting quality, the compliance rate was calculated with the number of items acquired “2 scores” based on the total number of included reports (except for “not applicable” numbers), which was further categorized as three levels: excellent compliance (> 90%), good compliance (between 50 and 90%), and poor compliance (< 50%). For the overall items of the CONSORT, the CONSORT for Abstract and ITCWM-specific checklists, the reporting score was recorded as mean and 95% Confidence Interval (CI). To clearly demonstration of the results, Figures were used to present the trend and comparison, while the Tables in Supplementary files were adopted to report the details. Subgroups comparisons, including i) “Journal Publications (both in English and Chinese)” vs “Published Dissertation (only in Chinese)” and ii) “English Publications” vs “Chinese Publications”, have been further analyzed, respectively. All data were collected and recorded in Microsoft Office Excel (Microsoft 365). Statistics analyses were performed using SPSS software, version 27.0.

## Results

### Search result

**Fig. 1:**
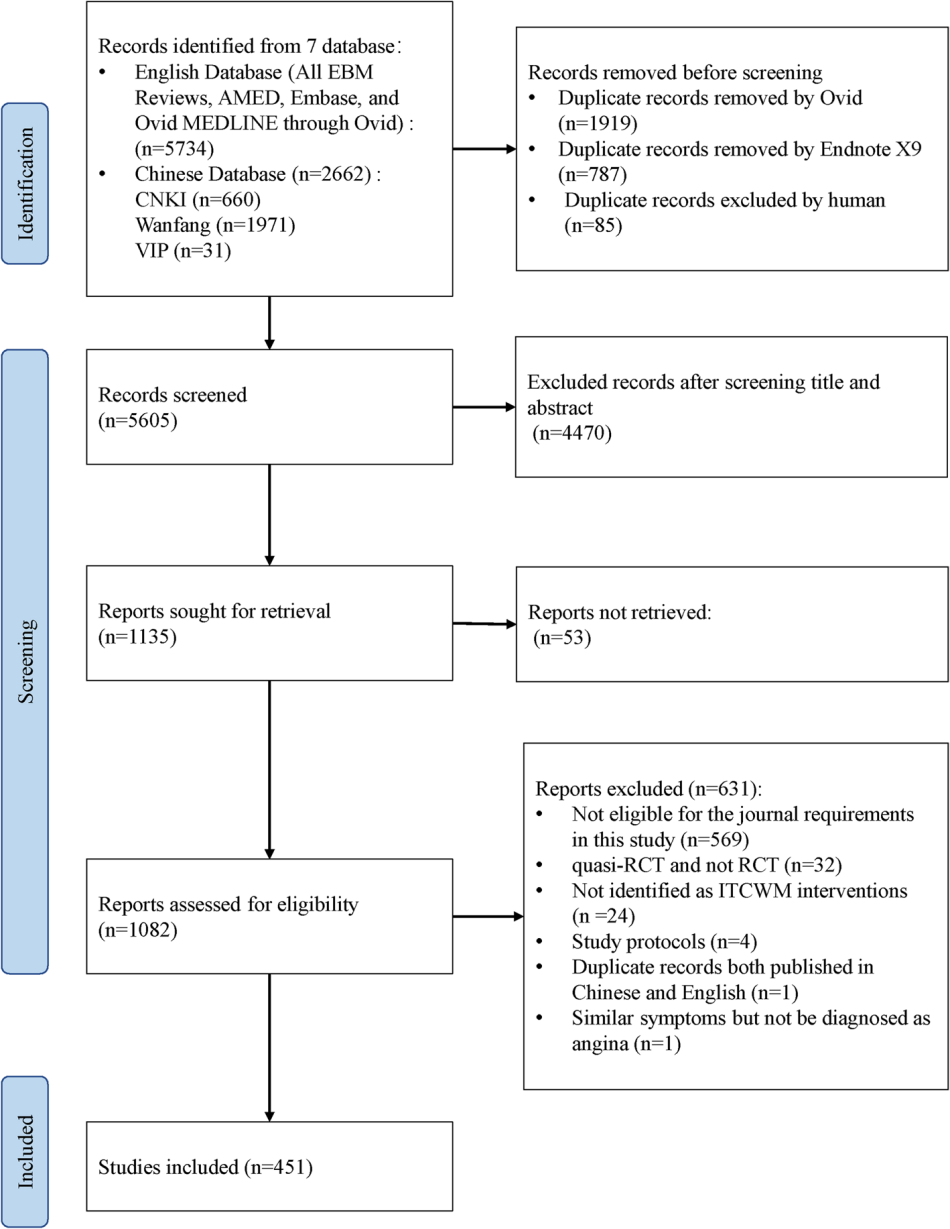
Flow chart of the search and selection process

The initial search identified a total of 8,396 records, among which 1,082 studies were retained after excluding duplication or title and abstract screening. After reviewing the full text, 451 ITCWM interventional RCTs of AP were included for analysis (Fig. 1). The lists of included and excluded articles are provided in Supplementary files 4 and 5.

### Characteristics of included trials

**Fig. 2:**
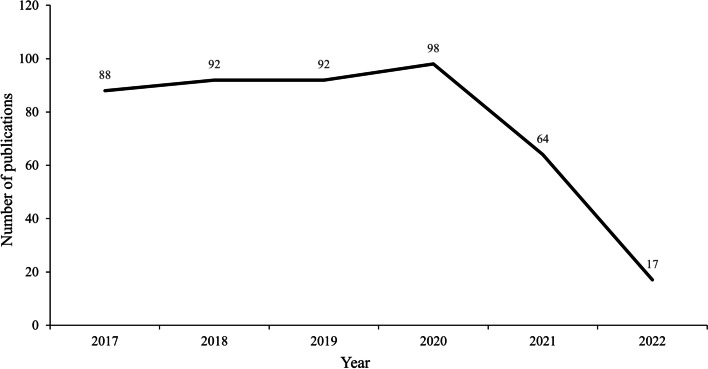
The number of publications during 2017–2022

**Fig. 3:**
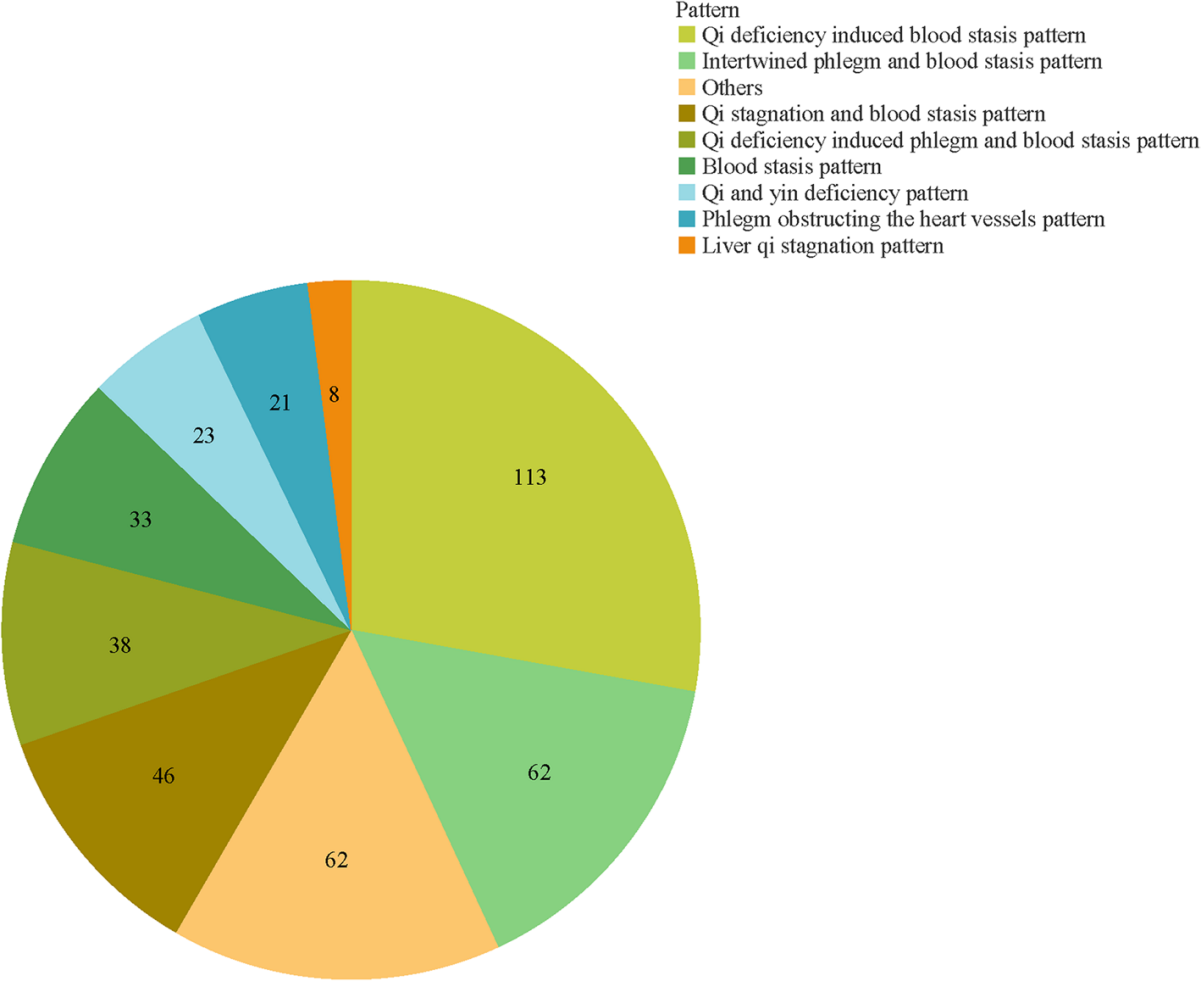
The TCM patterns of AP in the included articles

Among 451 included studies, the average number of publications was nearly 93 before 2021, while a decreased by 54% was identified afterword (Fig. 2). The most common integrative intervention was Chinese herbal formulas and western drugs. A total of 390 articles included TCM pattern criteria of AP diagnosis, of which the top 5 were *Qi deficiency induced blood stasis pattern*, *intertwined phlegm and blood stasis pattern*, *Qi stagnation and blood stasis pattern*, *Qi deficiency and intertwined phlegm and blood stasis pattern*, and *blood stasis pattern* (Fig. 3). More details are shown in Table 2 and Supplementary file 6.

**Table 2:** Characteristics of included studies (*n* = 451)

Characteristic	N (%)
Language of publications	
English	9 (2.00)
Chinese^a^	442 (98.00)
Diagnostic criteria	
Single western medicine diagnosis	49 (10.86)
Both TCM and western medicine diagnosis	402 (89.14)
Including TCM Pattern^b^	390 (97.01)
Types of AP	
Stable	225 (49.89)
Unstable	115 (25.50)
Others^c^	111 (24.61)
Sample size	
≤ 50	12 (2.66)
51–100	350 (77.61)
101–300	85 (18.85)
> 300	4 (0.89)
Number of assigned groups^d^	
2	438 (97.12)
3	7 (1.55)
4	6 (1.33)
Types of TCM interventions^e^	
Chinese herbal formula	389 (86.25)
Chinese single herb	10 (2.22)
Complex intervention	25 (5.54)
External therapy	27 (5.99)
Type of western medicine interventions	
Pharmacological treatment	439 (97.34)
Non-pharmacological treatment^f^	10 (2.22)
Not specific^g^	2 (0.44)
Treatment duration	
Median (IQR) /day	42 (28–56)
Follow-up duration	
Median (IQR) /day	117 (30–180)
Outcomes	
Including TCM-related indicators	390 (86.47)

^a^Including 259 Chinese dissertations

^b^198 of 390 studies included TCM disease diagnosis

^c^^, d, e^Supplementary file 6 for details

^f^Including 8 external counter pulsation, 1 percutaneous coronary intervention and 1 cardiac rehabilitation exercise

^g^Only reported the use of WM but without any description

### Ratings of overall quality and factors associated with reporting quality

**Fig. 4:**
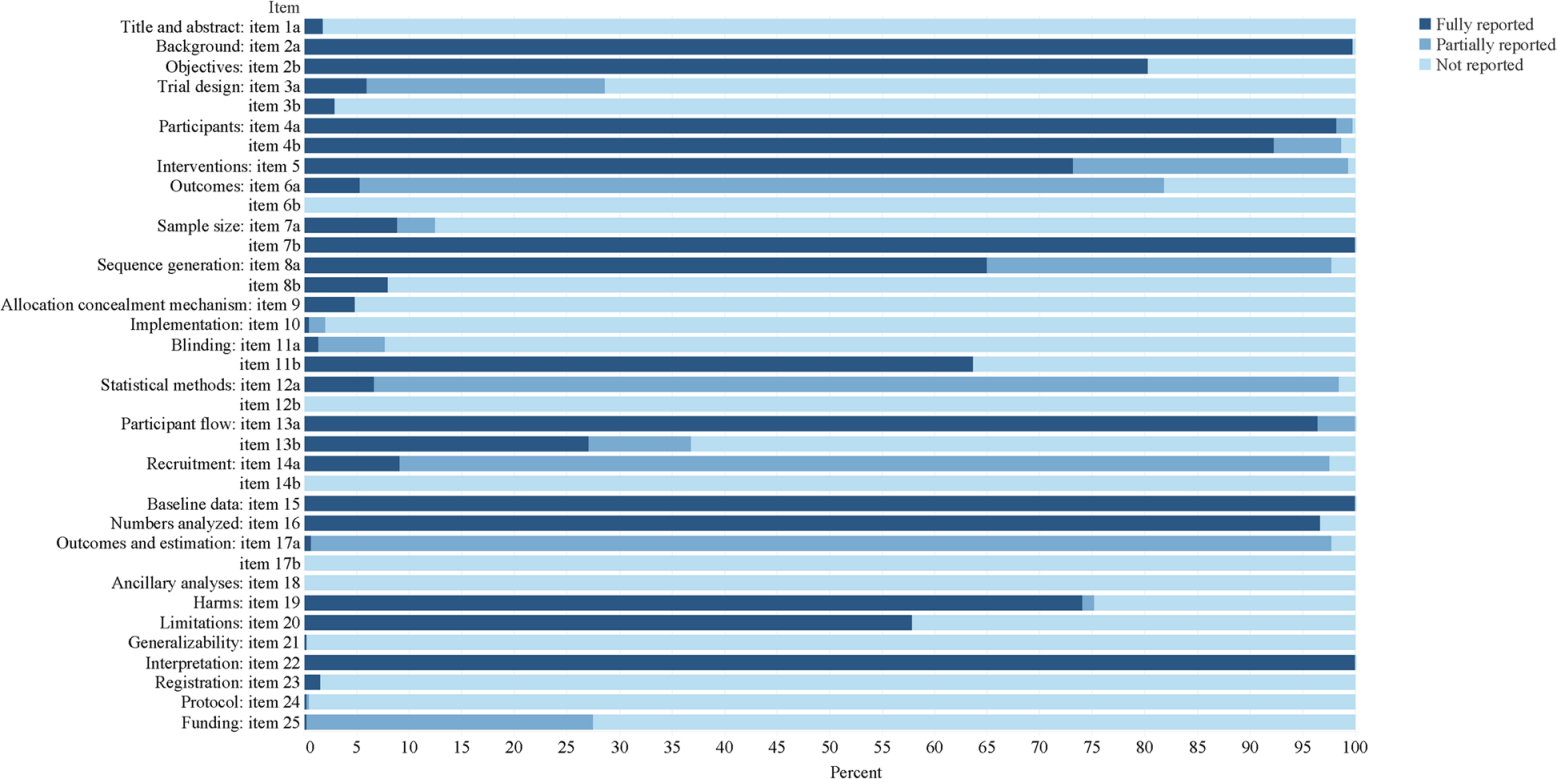
Reporting percentages of the CONSORT checklist

**Fig. 5:**
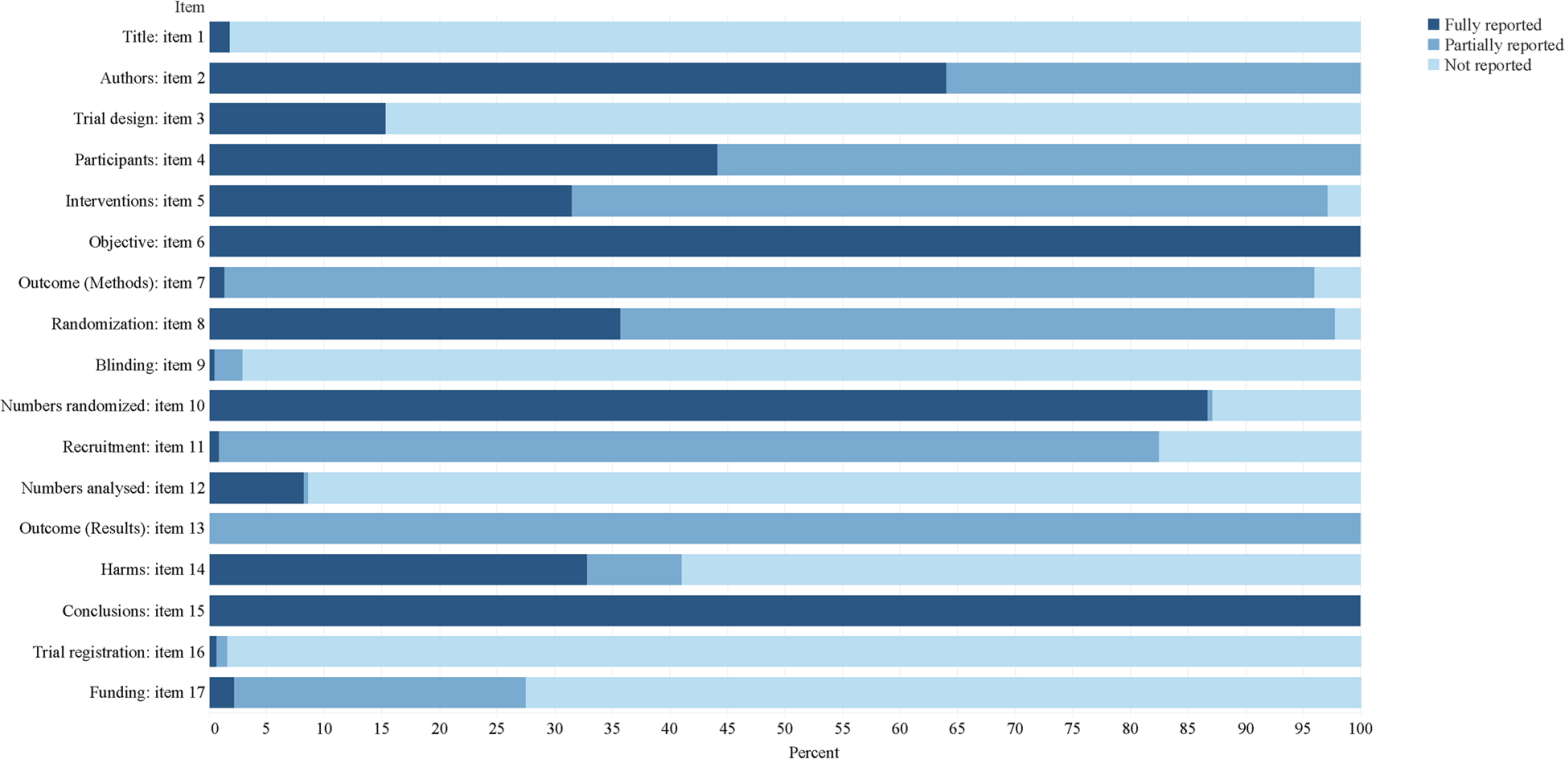
Reporting percentages of the CONSORT for Abstract checklist

**Fig. 6:**
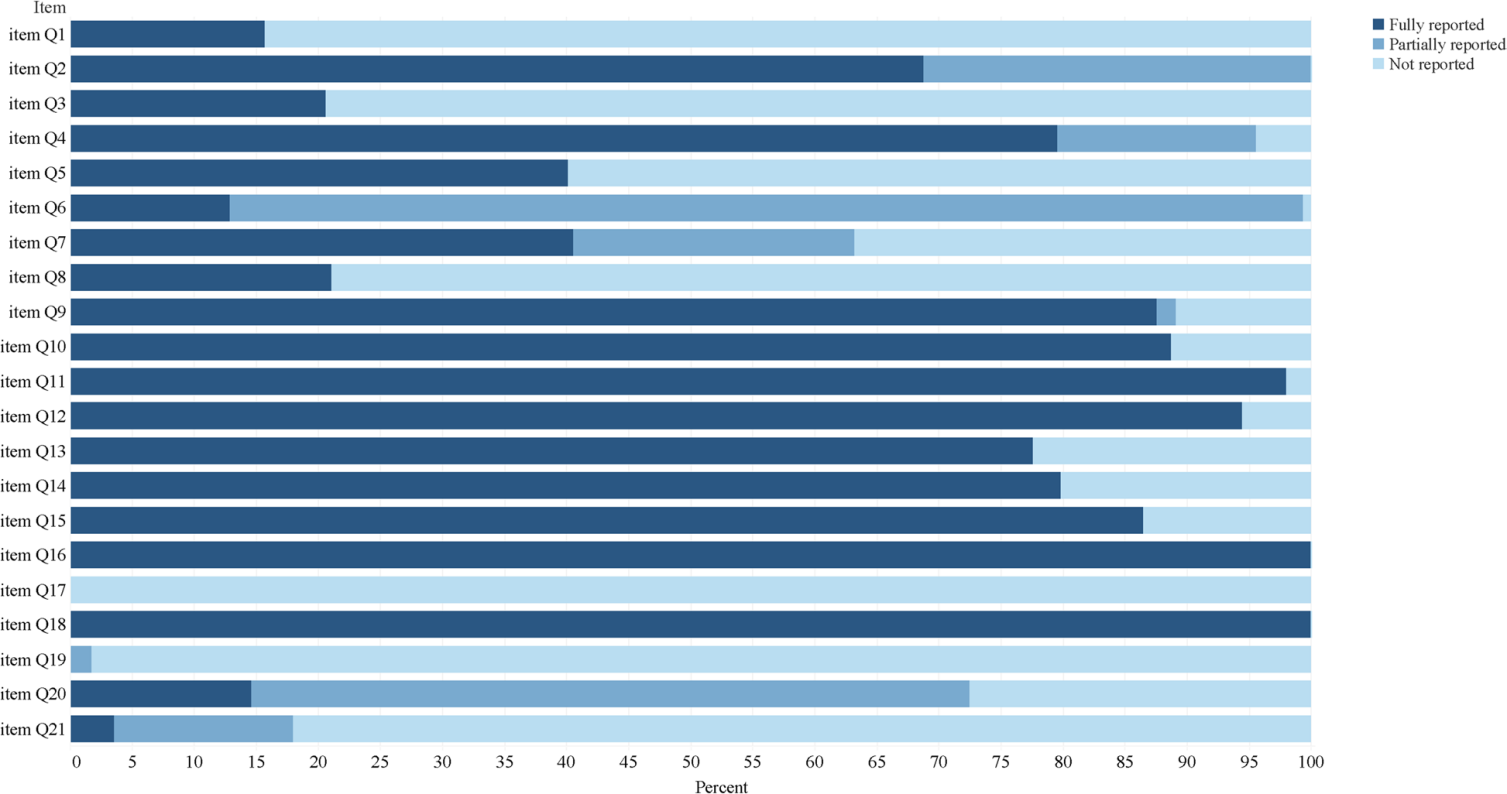
Reporting percentages of ITCWM-specific checklist

The results of adherence to the CONSORT, the CONSORT for Abstract, and ITCWM-specific checklist are presented in Figs. 4, 5 and 6. Together, more than half items were evaluated as poor quality (reporting rate < 50%) among each Checklist. For the completeness of the CONSORT checklist, the mean (95% CI) reporting score was 27.82 (27.44–28.19), including 8 items (2a, 4a, 4b, 7b, 13a, 15, 16 and 22) were assessed as excellent (> 90%). Moreover, the score for the CONSORT for Abstract was 14.17 (13.98–14.37) with only 2 items (6 and 15) assessed as excellent reporting (> 90%). In comparison, the score of the ITCWM-specific items was 21.06 (20.69–21.43), relatively low. Ten items were reported poorly (Q1, Q3, Q5, Q6, Q7, Q8, Q17, Q19, Q20 and Q21), of which 8 showed markedly low (< 30%), covering the identification of ITCWM in the title and keywords, provide objectives or hypotheses of ITCWM design, details for placebo design, baseline data relevant to ITCWM, interpretation of studied ITCWM interventions and conflicts of interests. During the assessments, the average value of Cohen κ for each item was more than 0.85 (Supplementary files 7 and 8).

**Fig. 7:**
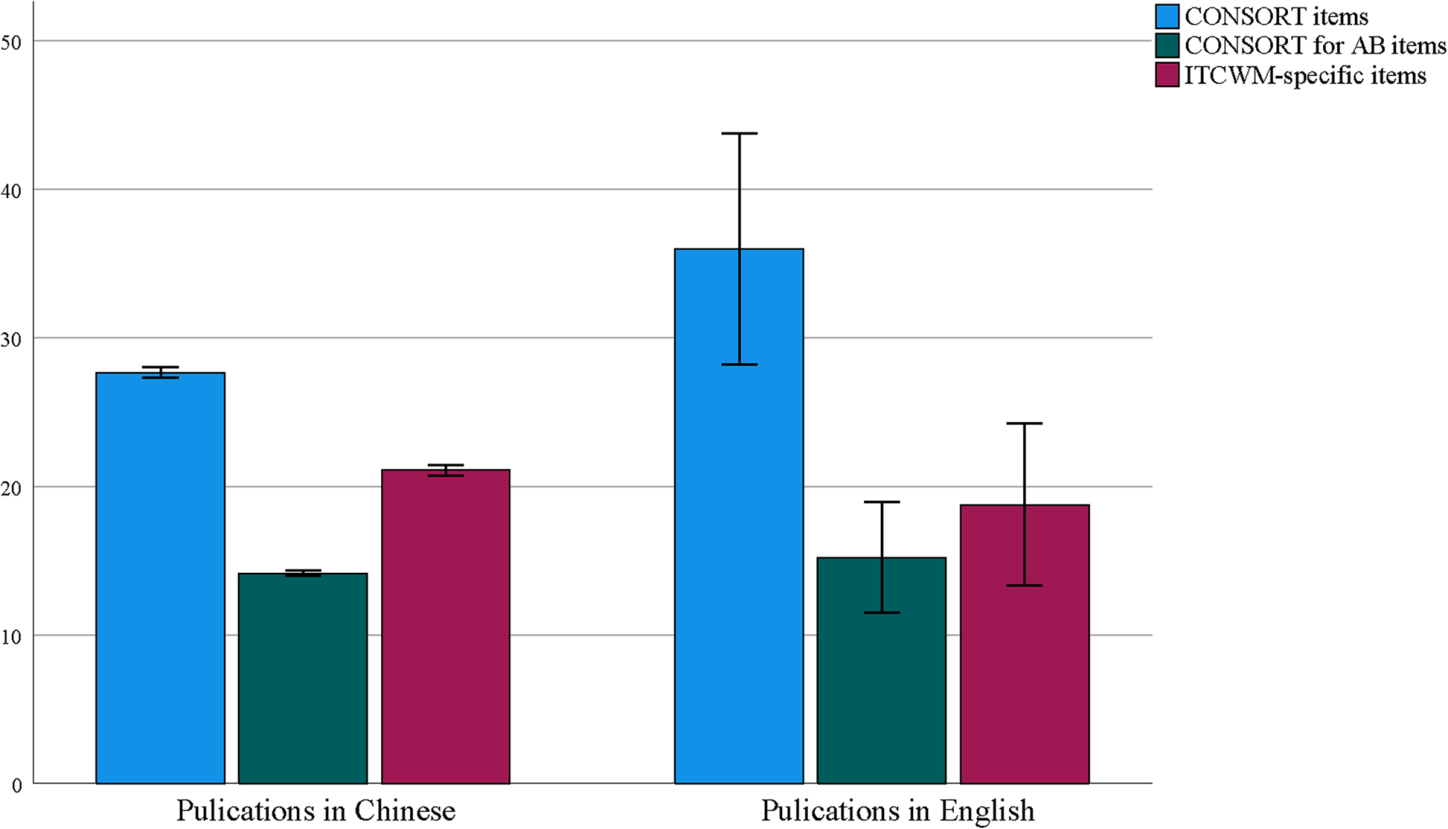
The subgroup analysis of publications in English and Chinese

**Fig. 8:**
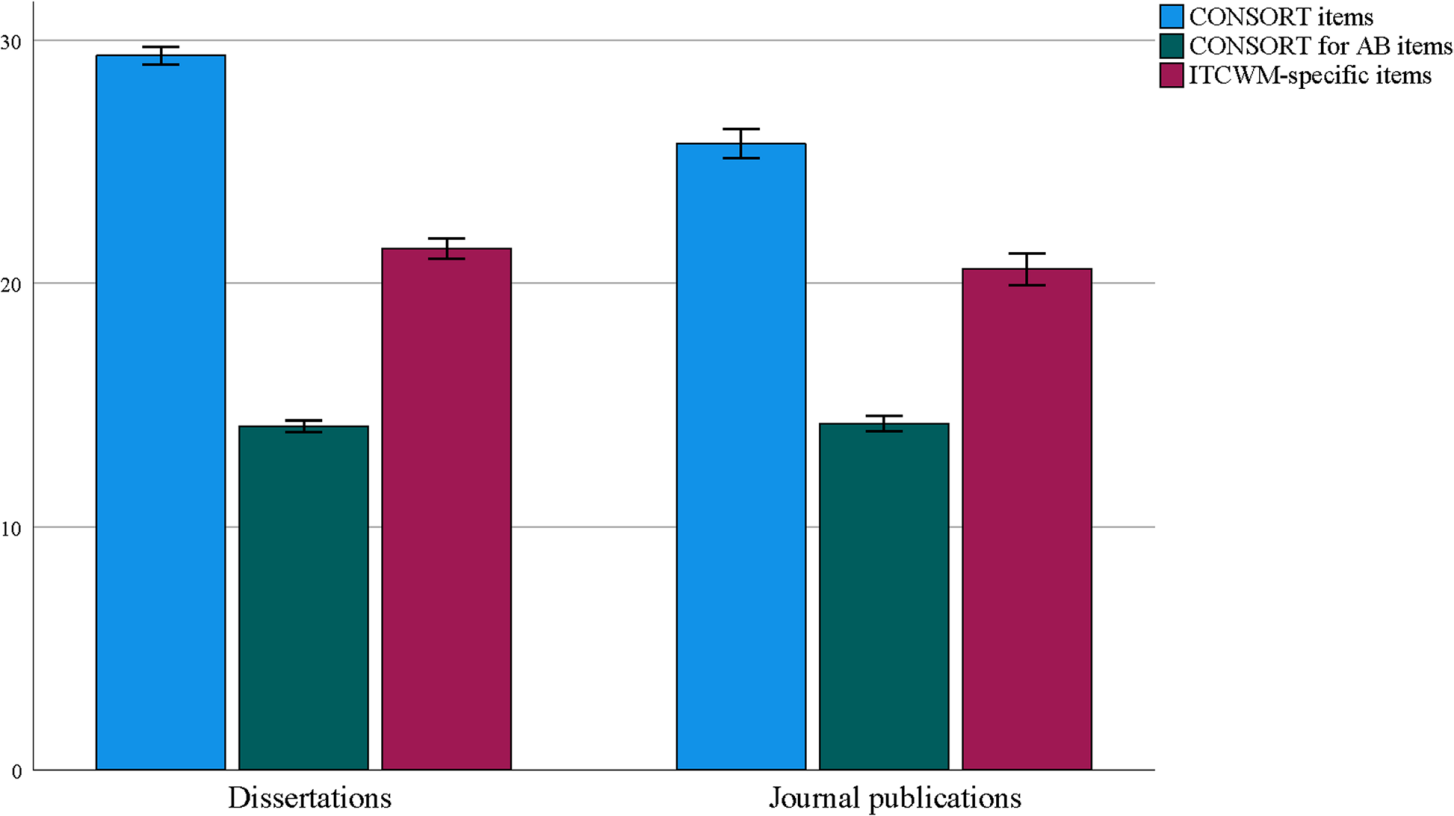
The subgroup analysis of dissertations and journal publications

For the subgroup analysis, the reporting score of publications in Chinese was lower than that in English regarding the CONSORT assessment (Fig. 7). Furthermore, the reporting of dissertations (in Chinese) was better than that in journal papers (including both English and Chinese) in the CONSORT and ITCWM-specific evaluation (Fig. 8). More details are provided in Supplementary file 9.

## Discussion

### General features of ITCWM interventional RCTs for AP

The number of ITCWM interventional RCTs for AP during 2017–2020 was increasing, despite a decrease arisen in 2021 (e.g., COVID-19 epidemic influence) [36]. Clear reporting of diagnosis is the key for ITCWM-related studies [37]. Although 89.14% studies diagnosed AP according to both TCM and WM criteria, however, some trials represented inadequate reporting, such as did not specify the types of AP, or TCM diagnosis reference. Through the calculation of TCM patterns included in the articles, three critical pattern elements of “*Qi deficiency*”, “*Intertwined phlegm*”, and “*Blood stasis*” were identified as major pathogenesis of cardiovascular disease, which is consistent with the mechanism recognition of WM [38]. Among TCM various interventions, Chinese herbal formula was the most common therapy (86.25%), but 13.53% studies did not include TCM related outcome for efficacy assessment. The inconsistent adoption of TCM pattern, Chinese herbal formula intervention, and TCM outcome assessments would induce potential bias in terms of appropriate evaluation for ITCWM designed studies [30].

### Quality assessment of the CONSORT

Regarding the reporting compliance of the CONSORT items, the average reporting rate was 35.63% (ranging from 0 to 100%). Twenty-two items were reported poorly (< 50%), most of which (50%, 11/22) related to methodological domains, such as trial design (item 3a and 3b), primary and secondary outcome measures (item 6a and 6b), sample size (item 7a), sequence generation (item 8b), allocation concealment mechanism (item 9), randomization implementation (item 10), blinding (item 11a), and statistical methods (item 12a and 12b). Previous studies have indicated that the methodology-related items are crucial for the assessment of bias risk and the reliability of reported effects [39–41]. Other items, including title (item 1a), losses and exclusions after randomization (item 13b), dates of recruitment and follow-up (item 14a and 14b), outcome (item 17a and 17b), ancillary analyses (item 18), generalizability (item 21), registration (item 23), protocol (item 24), and funding (item 25) were also reported poorly. Skepticism and criticism invited by inadequate reporting of trial registration, protocol, and ethical approval significantly damage the values of RCTs for AP [42]. In addition, a considerable number of the CONSORT for Abstract items were missing or incompletely reported; the average reporting rate was only 30.92%, with a range of 0 to 100%. 13 items (1, 3, 4, 5, 7, 8, 9, 11, 12, 13, 14, 16 and 17) were reported poorly, covering several sections of “Title”, “Trial design”, “Methods”, “Results” and “Other information”. In a manuscript, the “Abstract” is designed to highlight key points of the research, which provides crucial details to facilitate readers’ understanding and decide whether it is relevant to their interests [43]. This review found that the reporting of Abstracts should be urgently improved. Generally, the international journals have a better endorsement of the CONSORT than those in Chinese journals. In this review, we also found that the reporting score of the CONSORT in English papers was higher than that in Chinese papers, which is similar to previous studies [44]. However, there was no obvious improvements regarding the CONSORT for Abstract. Thus, more attention should be paid to the reporting of Abstracts of ITCWM interventional RCTs for AP.

### Quality assessment of ITCWM-specific items

For ITCWM items, the average reporting rate was 53.82%, with a range of 0% to 100%. The least reported information about ITCWM details can be summarized in the following aspects: identification of ITCWM in title and keywords, the rationale of the ITCWM was being used, objectives or hypotheses of ITCWM, trial design of ITCWM, and the description of ITCWM interventions. It is important for subsequent research in this field that readers can clearly distinguish different research areas from thousands of literatures and recognize the significance of the use of ITCWM therapy. The first identifier, with no doubt, includes the title and keywords. Further, the details of the implementations with related factors for quality control are essential for the replication of trials [45–47]. Unfortunately, in this study, we still found unsatisfactory reporting for ITCWM interventions although relevant reporting guidelines were issued, either the CONSORT 2010 or its Extensions (e.g., CONSORT Extension for Chinese Herbal Medicine Formulas 2017) [30]. In the comparison of different subgroups, we found that the reporting quality in Chinese Dissertations was better than those in journal publications regarding the ITCWM specifics. This could be attributed to the page and words limitations for Chinese journal publications [48]. Additionally, as the Dissertation is the basis for scholars to obtain their degree, higher requirements of quality were included. Thus, more attention should be paid on Dissertations in the field of ITCWM interventional RCTs for AP.

### Improvement measures and suggestions

According to the deficiencies of reporting identified in this study, specific improvements are needed. Previous findings confirm that guidelines do help improve the quality of reporting. Therefore, editors and reviewers should be more rigorous in their requirements that authors should adhere to the reporting standards of the CONSORT (especially for the CONSORT for Abstract) in RCTs. There are two paths forward: strengthen reporting of the CONSORT and develop a series of standard reporting items relevant to RCTs with ITCWM interventions [49].

## Limitations

This study has some limitations. First, we only identified RCTs of AP with ITCWM published from 1^st^ January 2017 to 6^th^ August 2022 in English and Chinese, so we have not assessed other trials published before 2017 or in other languages. Second, methodology quality (e.g., using a Cochrane tool) assessment was not conducted because this review focused on the reporting quality. Although the results of this review may not necessarily be comprehensive, we do believe that the general trends indicated by this study are valid. Besides, considering this study is not a systematic review and meta-analysis, we did not prospectively registration and publish its protocol.

## Conclusion

Without complete and transparent reporting of how a RCTs of AP with ITCWM was designed and implemented, it is difficult for readers to assess the reliability and validity of trial findings. Although the CONSORT does improve the quality of reporting, developing standard reporting items specifically relevant to ITCWM interventional RCTs as an extension to the CONSORT might be an effective means to achieving the improvement needed.

## Supplementary Information

**Additional file 1: Supplementary file 1.** PRISMA 2020 Checklist. **Supplementary file 2.** Search strategy. **Supplementary file 3.** Scoring rules of ITCWM items. **Supplementary file 4.** List of included articles in this study. **Supplementary file 5.** List of excluded articles (only in the step of full-text screening) in this study. **Supplementary file 6.** Specific information of Table 2 and Fig. 3. **Supplementary file 7.** Details of the CONSORT and ITCWM-specific items. **Supplementary file 8.** Details of the CONSORT for abstract and ITCWM-specific items. **Supplementary file 9.** Overall reporting scores for included studies, by subgroup.

## Data Availability

All data analyzed during this study are included in the manuscript and supplementary files.

## Abbreviations

AP: Angina pectoris
IHD: Ischemic heart diseases
MI: Myocardial infarction
TCM: Traditional Chinese medicine
ITCWM: Integrated traditional Chinese and western medicine
RCTs: Randomized controlled trials
WM: Western medicine
CNKI: China National Knowledge Infrastructure
E&E: Explanation and elaboration
IQR: Interquartile range
CI: Confidence interval
