# Supplementary material for: Reporting quality of randomized controlled trials of angina pectoris with integrated traditional Chinese and western medicine interventions: a cross-sectional study
Source: BMC Med Res Methodol. 2023 May 23;23:124. doi: 10.1186/s12874-023-01953-1 (PMC10204018; doi:10.1186/s12874-023-01953-1)
Supplement: Supplementary file 1 — Additional file 1: Supplementary file 1. PRISMA 2020 Checklist. Supplementary file 2. Search strategy. Supplementary file 3. Scoring rules of ITCWM items. Supplementary file 4. List of included articles in this study. Supplementary file 5. List of excluded articles (only in the step of full-text screening) in this study. Supplementary file 6. Specific information of Table 2 and Fig. 3. Supplementary file 7. Details of the CONSORT and ITCWM-specific items. Supplementary file 8. Details of the CONSORT for abstract and ITCWM-specific items. Supplementary file 9. Overall reporting scores for included studies, by subgroup. [file 12874_2023_1953_MOESM1_ESM.docx]

Contents

[Supplementary file 1 PRISMA 2020 Checklist. 2](#_Toc132439311)

[Supplementary file 2 Search strategy 6](#_Toc132439312)

[Supplementary file 3 Scoring rules of ITCWM items 11](#_Toc132439318)

[Supplementary file 4 List of included articles in this study 20](#_Toc132439319)

[Supplementary file 5 List of excluded articles (only in the step of full-text screening) in this study 51](#_Toc132439320)

[Supplementary file 6 Specific information of Specific information of Table 2 and Figure 3 93](#_Toc132439321)

[Supplementary file 7 Details of the CONSORT and ITCWM-specific items 98](#_Toc132439327)

[Supplementary file 8 Details of the CONSORT for abstract and ITCWM-specific items. 105](#_Toc132439328)

[Supplementary file 9 Overall reporting scores for included studies, by subgroup 107](#_Toc132439329)

**Supplementary file 1 PRISMA 2020 Checklist.**

| **Section and Topic** | **Item #** | **Checklist item** | **Location where item is reported** |
| --- | --- | --- | --- |
| **TITLE** | | |  |
| Title | 1 | Identify the report as a systematic review. | 1 |
| **ABSTRACT** | | |  |
| Abstract | 2 | See the PRISMA 2020 for Abstracts checklist. | 2-3 |
| **INTRODUCTION** | | |  |
| Rationale | 3 | Describe the rationale for the review in the context of existing knowledge. | 3-5 |
| Objectives | 4 | Provide an explicit statement of the objective(s) or question(s) the review addresses. | 5 |
| **METHODS** | | |  |
| Eligibility criteria | 5 | Specify the inclusion and exclusion criteria for the review and how studies were grouped for the syntheses. | 5-6 |
| Information sources | 6 | Specify all databases, registers, websites, organisations, reference lists and other sources searched or consulted to identify studies. Specify the date when each source was last searched or consulted. | 6 |
| Search strategy | 7 | Present the full search strategies for all databases, registers and websites, including any filters and limits used. | 6 |
| Selection process | 8 | Specify the methods used to decide whether a study met the inclusion criteria of the review, including how many reviewers screened each record and each report retrieved, whether they worked independently, and if applicable, details of automation tools used in the process. | 6 |
| Data collection process | 9 | Specify the methods used to collect data from reports, including how many reviewers collected data from each report, whether they worked independently, any processes for obtaining or confirming data from study investigators, and if applicable, details of automation tools used in the process. | 7 |
| Data items | 10a | List and define all outcomes for which data were sought. Specify whether all results that were compatible with each outcome domain in each study were sought (e.g. for all measures, time points, analyses), and if not, the methods used to decide which results to collect. | 7 |
|  | 10b | List and define all other variables for which data were sought (e.g. participant and intervention characteristics, funding sources). Describe any assumptions made about any missing or unclear information. | 7 |
| Study risk of bias assessment | 11 | Specify the methods used to assess risk of bias in the included studies, including details of the tool(s) used, how many reviewers assessed each study and whether they worked independently, and if applicable, details of automation tools used in the process. | NA (13 limitations) |
| Effect measures | 12 | Specify for each outcome the effect measure(s) (e.g. risk ratio, mean difference) used in the synthesis or presentation of results. | 8 |
| Synthesis methods | 13a | Describe the processes used to decide which studies were eligible for each synthesis (e.g. tabulating the study intervention characteristics and comparing against the planned groups for each synthesis (item #5)). | 7-8 |
|  | 13b | Describe any methods required to prepare the data for presentation or synthesis, such as handling of missing summary statistics, or data conversions. | 7-8 |
|  | 13c | Describe any methods used to tabulate or visually display results of individual studies and syntheses. | 8 |
|  | 13d | Describe any methods used to synthesize results and provide a rationale for the choice(s). If meta-analysis was performed, describe the model(s), method(s) to identify the presence and extent of statistical heterogeneity, and software package(s) used. | 7-8 |
|  | 13e | Describe any methods used to explore possible causes of heterogeneity among study results (e.g. subgroup analysis, meta-regression). | 8 |
|  | 13f | Describe any sensitivity analyses conducted to assess robustness of the synthesized results. | 8 |
| Reporting bias assessment | 14 | Describe any methods used to assess risk of bias due to missing results in a synthesis (arising from reporting biases). | NA (13 limitations) |
| Certainty assessment | 15 | Describe any methods used to assess certainty (or confidence) in the body of evidence for an outcome. | 8 |
| **RESULTS** | | |  |
| Study selection | 16a | Describe the results of the search and selection process, from the number of records identified in the search to the number of studies included in the review, ideally using a flow diagram. | 9 |
|  | 16b | Cite studies that might appear to meet the inclusion criteria, but which were excluded, and explain why they were excluded. | 9 (Supplementary 5) |
| Study characteristics | 17 | Cite each included study and present its characteristics. | 9 |
| Risk of bias in studies | 18 | Present assessments of risk of bias for each included study. | NA (13 limitations) |
| Results of individual studies | 19 | For all outcomes, present, for each study: (a) summary statistics for each group (where appropriate) and (b) an effect estimate and its precision (e.g. confidence/credible interval), ideally using structured tables or plots. | Fig 4-8, Supplementary 6-9 |
| Results of syntheses | 20a | For each synthesis, briefly summarise the characteristics and risk of bias among contributing studies. | 9-10 |
|  | 20b | Present results of all statistical syntheses conducted. If meta-analysis was done, present for each the summary estimate and its precision (e.g. confidence/credible interval) and measures of statistical heterogeneity. If comparing groups, describe the direction of the effect. | 9-10 |
|  | 20c | Present results of all investigations of possible causes of heterogeneity among study results. | 10 |
|  | 20d | Present results of all sensitivity analyses conducted to assess the robustness of the synthesized results. | 10 |
| Reporting biases | 21 | Present assessments of risk of bias due to missing results (arising from reporting biases) for each synthesis assessed. | NA (13 limitations) |
| Certainty of evidence | 22 | Present assessments of certainty (or confidence) in the body of evidence for each outcome assessed. | 10 |
| **DISCUSSION** | | |  |
| Discussion | 23a | Provide a general interpretation of the results in the context of other evidence. | 10-13 |
|  | 23b | Discuss any limitations of the evidence included in the review. | 13 |
|  | 23c | Discuss any limitations of the review processes used. | 13 |
|  | 23d | Discuss implications of the results for practice, policy, and future research. | 13 |
| **OTHER INFORMATION** | | |  |
| Registration and protocol | 24a | Provide registration information for the review, including register name and registration number, or state that the review was not registered. | 13 |
|  | 24b | Indicate where the review protocol can be accessed, or state that a protocol was not prepared. | 14 |
|  | 24c | Describe and explain any amendments to information provided at registration or in the protocol. | NA |
| Support | 25 | Describe sources of financial or non-financial support for the review, and the role of the funders or sponsors in the review. | 15 |
| Competing interests | 26 | Declare any competing interests of review authors. | 15 |
| Availability of data, code and other materials | 27 | Report which of the following are publicly available and where they can be found: template data collection forms; data extracted from included studies; data used for all analyses; analytic code; any other materials used in the review. | 16 |

*From:*  Page MJ, McKenzie JE, Bossuyt PM, Boutron I, Hoffmann TC, Mulrow CD, et al. The PRISMA 2020 statement: an updated guideline for reporting systematic reviews. BMJ 2021;372:n71. doi: 10.1136/bmj.n71

For more information, visit: <http://www.prisma-statement.org/>

**Supplementary file 2 Search strategy**

1. English Databases, including **All EBM Reviews** (EBM Reviews - ACP Journal Club <1991 to July 2022>, EBM Reviews - Cochrane Central Register of Controlled Trials <July 2022>, EBM Reviews - Cochrane Database of Systematic Reviews <2005 to August 3, 2022>, EBM Reviews - Cochrane Clinical Answers <July 2022>, EBM Reviews - Cochrane Methodology Register <3rd Quarter 2012>, EBM Reviews - Database of Abstracts of Reviews of Effects <1st Quarter 2016>, EBM Reviews - Health Technology Assessment <4th Quarter 2016>, EBM Reviews - NHS Economic Evaluation Database <1st Quarter 2016>), **AMED** (Allied and Complementary Medicine) <1985 to August 2022>, **Embase** <1974 to 2022 August 05>, **Ovid MEDLINE(R)** <1946 to August 05, 2022>.

| Search Date: August 6, 2022 | |
| --- | --- |
| Search formula: | |
| 1 | exp Angina Pectoris/ |
| 2 | angina*.tw. |
| 3 | stenocardia*.tw. |
| 4 | angor pectoris.tw. |
| 5 | or 1-4 |
| 6 | randomized controlled trial/ |
| 7 | RCT.tw. |
| 8 | controlled clinical trial/ |
| 9 | random$.tw. |
| 10 | (crossover$ or cross over$ or cross-over$).tw. |
| 11 | placebo$.tw. |
| 12 | single blind.mp. |
| 13 | double blind.mp. |
| 14 | triple blind.mp. |
| 15 | (singl$ adj blind$).tw. |
| 16 | (double$ adj blind$).tw. |
| 17 | (tripl$ adj blind$).tw. |
| 18 | assign$.tw. |
| 19 | allocat$.tw. |
| 20 | or 6-19 |
| 21 | exp Drugs, Chinese Herbal/ or Chinese Herbal Drugs.mp. |
| 22 | Chinese Traditional Medicine.mp. |
| 23 | Chinese medicine$ or traditional medicine$.mp. |
| 24 | herbal drug$ or herbal medicine$.mp. |
| 25 | herb$ or herb$ formula$ or decoction$.mp. |
| 26 | Chinese Medicine Patent Prescription or proprietary Chinese medicines.mp. |
| 27 | Chinese patent adj3 (medicine or drug$).mp. |
| 28 | or 21-27 |
| 29 | exp Acupuncture Therapy/ |
| 30 | exp Acupuncture/ |
| 31 | Electroacupuncture.tw,kf. |
| 32 | acupuncture.tw,kf. |
| 33 | Acupoint*.tw. |
| 34 | exp Massage/ |
| 35 | massage* or tuina:ti,ab,kw |
| 36 | Moxibustion/ |
| 37 | (moxa or moxibustion).mp. |
| 38 | meridian*.tw,kf. |
| 39 | Acupoint application.mp. |
| 40 | Auricular-laster.mp. |
| 41 | Cupping.mp. |
| 42 | Fumigation.mp. |
| 43 | Guasha.mp. |
| 44 | Daoyin or Qigong or taijiquan or baduanjin.mp. |
| 45 | or 29-44 |
| 46 | Integrated adj1 Chinese and western medicine.tw. |
| 47 | Combination of Chinese and western medicine.tw. |
| 48 | Chinese and western medicine.tw. |
| 49 | Combined Chinese and western medicine.tw. |
| 50 | Western medicine.tw. |
| 51 | myocardial infarction or MI.mp. |
| 52 | coronary artery bypass graft or CABG.mp. |
| 53 | percutaneous coronary intervention or PCI.mp. |
| 54 | Revascularization or Intervention$ or surgery.mp. |
| 55 | Nitrate*.mp. |
| 56 | Beta-blocker*.mp. |
| 57 | Calcium channel blockers.mp. |
| 58 | Ivabradine.mp. |
| 59 | Nicorandil.mp. |
| 60 | Ranolazine.mp. |
| 61 | Trimetazidine.mp. |
| 62 | Allopurinol.mp. |
| 63 | antiplatelet.mp. |
| 64 | antithrombotic.mp. |
| 65 | Proton pump inhibitors.mp. |
| 66 | Statin* or Lipid-lowering medication*.mp. |
| 67 | ACE inhibitors or ACEI or angiotensin receptor blocker or ARB |
| 68 | aspirin.mp. |
| 69 | clopidogrel.mp. |
| 70 | Amlodipine.mp. |
| 71 | or 46-70 |
| 72 | 28 or 45 or 71 |
| 73 | 5 and 20 and 72 |
| 74 | limit 73 to yr="2017 -Current" |
| 75 | remove duplicates from 74 |
| Result | 3815 |

1. Chinese Databases, including CNKI, VIP and Wanfang.

CNKI:

| Search Date: August 6, 2022 | |
| --- | --- |
| Search Range: 1^st^ Jan 2017 - August 6, 2022 | |
| 1 | SU=('心绞痛'+'稳定性心绞痛'+'稳定型心绞痛'+'不稳定性心绞痛'+'不稳定型心绞痛'+'变异性心绞痛'+'微血管心绞痛'+'冠心病心绞痛'+'心肌缺血'+'心痛'+'胸痹') AND SU=('中医'+'中医药'+'中药'+'复方'+'汤剂'+'中成药'+'注射剂 '+'加减方'+'针灸'+'针刺'+'穴位 '+'经络'+'灸'+'推拿'+'电针'+'敷贴'+'拔罐'+'熏蒸'+'刮痧'+'导引'+'气功'+'太极拳'+'八段锦') AND SU=('中西医结合'+'中西结合'+'中西医联合'+'中西联用'+'中西医结合疗法'+'中西医结合试验'+'中西医结合治疗'+'中西医'+'中西'+'冠状动脉旁路搭桥术'+'搭桥'+'经皮冠状动脉介入治疗'+'介入'+'手术'+'硝酸甘油'+'倍他洛克'+'倍他乐克'+'β受体阻滞剂'+'美托洛尔'+'钙通道阻滞剂'+'伊伐布雷定'+'尼可地尔'+'雷诺嗪'+'曲美他嗪'+'抗血小板'+'抗凝'+'他汀'+'血管紧张素转换酶抑制剂'+'ACEI'+'血管紧张素受体阻滞剂'+'ARB'+'阿司匹林'+'氯吡格雷'+'氨氯地平') AND SU=('临床试验'+'随机'+'对照'+'随机试验'+'随机对照试验'+'临床研究') |
| Result | 660 |

WanFang:

| Search Date: August 6, 2022 | |
| --- | --- |
| Search Range: 2017 - August 6, 2022 | |
| 1 | 主题:(( 心绞痛 or 稳定性心绞痛 or 稳定型心绞痛 or 不稳定性心绞痛 or 不稳定型心绞痛 or 变异性心绞痛 or 微血管心绞痛 or 冠心病心绞痛 or 心肌缺血 or 心痛 or 胸痹 ) and (中医 or 中医药 or 中药 or 复方 or 汤剂 or 中成药 or 注射剂 or 加减方 or 针灸 or 针刺 or 穴位 or 经络 or 灸 or 推拿 or 电针 or 敷贴 or 拔罐 or 熏蒸 or 刮痧 or 导引 or 气功 or 太极拳 or 八段锦 ) and (中西医结合 or 中西结合 or 中西医联合 or 中西联用 or 中西医结合疗法 or 中西医结合试验 or 中西医结合治疗 or 中西医 or 中西 or 冠状动脉旁路搭桥术 or 搭桥 or 经皮冠状动脉介入治疗 or 介入 or 手术 or 硝酸甘油 or 倍他洛克 or 倍他乐克 or β受体阻滞剂 or 美托洛尔 or 钙通道阻滞剂 or 伊伐布雷定 or 尼可地尔 or 雷诺嗪 or 曲美他嗪 or 抗血小板 or 抗凝 or 他汀 or 血管紧张素转换酶抑制剂 or ACEI or 血管紧张素受体阻滞剂 or ARB or 阿司匹林 or 氯吡格雷 or 氨氯地平 ) and (临床试验 or 随机 or 对照 or 随机试验 or 随机对照试验 or 临床研究 )) |
| Result | 1971 |

VIP:

| Search Date: August 6, 2022 | |
| --- | --- |
| Search Range: 2017 - August 6, 2022 | |
| 1 | M=(心绞痛 OR 稳定性心绞痛 OR 稳定型心绞痛 OR 不稳定性心绞痛 OR 不稳定型心绞痛 OR 变异性心绞痛 OR 微血管心绞痛 OR 冠心病心绞痛 OR 心肌缺血 OR 心痛 OR 胸痹) AND M=(中医 OR 中医药 OR 中药 OR 复方 OR 汤剂 OR 中成药 OR 注射剂 OR 加减方 OR 针灸 OR 针刺 OR 穴位 OR 经络 OR 灸 OR 推拿 OR 电针 OR 敷贴 OR 拔罐 OR 熏蒸 OR 刮痧 OR 导引 OR 气功 OR 太极拳 OR 八段锦) AND M=(中西医结合 OR 中西结合 OR 中西医联合 OR 中西联用 OR 中西医结合疗法 OR 中西医结合试验 OR 中西医结合治疗 OR 中西医 OR 中西 OR 冠状动脉旁路搭桥术 OR 搭桥 OR 经皮冠状动脉介入治疗 OR 介入 OR 手术 OR 硝酸甘油 OR 倍他洛克 OR 倍他乐克 OR β受体阻滞剂 OR 美托洛尔 OR 钙通道阻滞剂 OR 伊伐布雷定 OR 尼可地尔 OR 雷诺嗪 OR 曲美他嗪 OR 抗血小板 OR 抗凝 OR 他汀 OR 血管紧张素转换酶抑制剂 OR ACEI OR 血管紧张素受体阻滞剂 OR ARB OR 阿司匹林 OR 氯吡格雷 OR 氨氯地平) AND (临床试验 OR 随机 OR 对照 OR 随机试验 OR 随机对照试验 OR 临床研究) |
| Result | 31 |

*Notes:* Translating Search strategy of Chinese databases in English

心绞痛=angina pectoris; 稳定性心绞痛+稳定型心绞痛=stable angina pectoris; 不稳定性心绞痛+不稳定型心绞痛=unstable angina pectoris; 变异性心绞痛= variant angina pectoris; 微血管心绞痛= microvascular angina pectoris; 冠心病心绞痛= coronary atherosclerotic heart disease and angina pectoris; 心肌缺血= Myocardial ischemia; 心痛=xin tong; 胸痹=xiong bi; 中医+中医药=traditional Chinese medicine; 中药= Chinese herbal medicine; 复方= compound formulas; 汤剂= decoction; 中成药= Chinese patent medicine; 注射剂=injection; 加减方= modified formula; 针灸= acupuncture and moxibustion; 针刺= acupuncture; 穴位=acupoint; 经络= meridian; 灸= moxibustion; 推拿=massage; 电针= electroacupuncture; 敷贴=acupoint application; 拔罐= cupping; 熏蒸= fumigation; 刮痧=Guasha; 导引=daoyin;气功=qigong; 太极拳=taijiquan; 八段锦=baduanjin; 中西医结合+中西结合+中西医联合+中西联用+中西医结合疗法+中西医结合试验+中西医结合治疗= integrated/integrative/combined Chinese and western medicine; 中西医+中西= Chinese and western medicine; 冠状动脉旁路搭桥术+搭桥= coronary artery bypass graft; 经皮冠状动脉介入治疗+介入= percutaneous coronary intervention; 手术= surgery; 硝酸甘油= Nitroglycerin; 倍他洛克+倍他乐克+β受体阻滞剂+美托洛尔= Beta-blocker; 钙通道阻滞剂= Calcium channel blockers; 伊伐布雷定= Ivabradine; 尼可地尔= Nicorandil; 雷诺嗪= Ranolazine; 曲美他嗪= Trimetazidine; 抗血小板= antiplatelet; 抗凝= antithrombotic; 他汀= Statin; 血管紧张素转换酶抑制剂+ACEI= ACE inhibitors; 血管紧张素受体阻滞剂+ARB= angiotensin receptor blocker; 阿司匹林=Aspirin; 氯吡格雷=Clopidogrel; 氨氯地平= Amlodipine; 临床试验= clinical trial; 随机= random; 对照=control; 随机试验=randomized trial; 随机对照试验= randomized controlled trial; 临床研究=clinical study

**Supplementary file 3 Scoring rules of ITCWM items**

| **Section** | **Q No.** | **Specifics** | **Definition of Fully reported (scored as 2), Partially reported (scored as 1), Not reported (scored as 0) and Not Applicable (did not be calculated)** | **Example(s) of “Fully reported”** |
| --- | --- | --- | --- | --- |
| Title | Q1 | Whether the feature of ITCWM was presented in Title? | “Fully reported” was considered if the reader can determine ITCWM design from the title. Generally, it was presented as follows: i) the word “ITCWM” (similar words) was used; ii) reported as specific names of TCM interventions combined with WM interventions in the title.  “Not reported” was considered if title could not be identified as ITCWM design.  “Partially reported” was not identified in this item.  “Not applicable” was not identified in this item. | 1. “Therapeutic effect of modified Dachaihu Decoction combined with Clopidogrel Bisulfate Tablets and effects on IL-37, ILT3 level in patients with angina pectoris” 2. “Clinical effect of Yiqihuoxue formula combined with western medicine in the treatment of the angina pectoris” |
| Abstract | Q2 | Whether the eligibility criteria of participants included both Chinese and western medical diagnosis in Methods of Abstract? | “Fully reported” was considered if both TCM and Western criteria for AP were reported in the Method of Abstract.  “Partially reported” was considered if there was only WM or TCM criteria (or pattern name) of AP.  “Not reported” was considered if there were no diagnosis in the Methods of Abstract.  “Not applicable” was not identified in this item. | 1. “To observe the clinical effect of Yiqi Fumai Lyophilized Injection combined with nicorandil in the treatment of microvascular angina with Qi and Yin deficiency.” |
|  | Q3 | Whether the study objectives or hypotheses were focused on the ITCWM interventions in Abstract? | “Fully reported” was considered if the reader can identify the ITCWM design in objectives or hypotheses. Generally, it was presented as follows: i) the word “ITCWM” (or similar words) was used; ii) reported as specific names of TCM interventions combined with WM interventions in the title.  “Not reported” was considered if there was no description relevant to ITCWM.  “Not applicable” refers to there is no objective or hypotheses in Abstract.  “Partially reported” was not identified in this item. | 1. “To observe the therapeutic effect of Gualouxiebaibanxia Decoction combined with Xuefuzhuyu Decoction and western medicine in treatment of Xiongbi (angina pectoris).” 2. “To explore the therapeutic effect of integrated traditional Chinese and western medicine on unstable angina pectoris.” |
|  | Q4 | Whether the outcome measures included both TCM and WM related endpoints in Abstract? | “Fully reported” was considered if both TCM and WM related endpoints were reported in Methods of Abstract.  “Partially reported” was considered if there was only WM or TCM related endpoints in Methods of Abstract.  “Not reported” was considered if no specific endpoints were reported in Methods of Abstract.  “Not applicable” was not identified in this item. | 1. “After six months of continuous treatment, the clinical efficacy, traditional Chinese medicine (TCM) syndrome scores, Pittsburgh Sleep Quality Index (PSQI), and the level of coronary CT vascular stenosis were observed.” |
|  | Q5 | Whether the effect of studied ITCWM interventions was reported in Conclusion of Abstract? | “Fully reported” was considered if ITCWM can be identified in Conclusion of Abstract, including the word “ITCWM” or the specific names of TCM interventions combined with WM interventions.  “Not reported” was considered if there was no description relevant to ITCWM.  “Partially reported” was not identified in this item.  “Not applicable” was not identified in this item. | 1. “Compound Danshen dropping pill combined with clopidogrel were effective in the treatment of angina pectoris.” |
| Key words | Q6 | Whether the feature or design of ITCWM study were reflected in Keywords? | “Fully reported” was considered if i) the word “ITCWM” (similar words) was used; or ii) reported as specific names of TCM interventions combined with WM interventions in the title.  “Partially reported” was considered if Keywords only included specific names of TCM or WM interventions.  “Not reported” was considered if there were not Keywords or any interventions in the Keywords.  “Not applicable” was not identified in this item. | 1. “Angina pectoris; Buben-jieyu granules; Percutaneous coronary intervention; integrated TCM and WM; Treatment outcome” 2. “Dachaihu Decoction; Clopidogrel Bisulfate Tablets; coronary heart disease; angina pectoris; antimicrobial peptides; immunoglobulin like transcript;” |
| Introduction | Q7 | Whether the reason/rationale about ITCWM intervention for the study design was reported in the Background? | “Fully reported” was considered if the advantage or disadvantage of TCM and WM, or the necessity of ITCWM were reported in the treatment of angina pectoris in Introduction.  “Partially reported” was considered if there was only reason of TCM or WM intervention in Introduction.  “Not reported” was considered if no description of reason of TCM or WM intervention in Introduction.  “Not applicable” was not identified in this item. | 1. “Stable angina is the most common type of AP... Nowadays, pharmaceutical therapy, PCI, surgery, etc. were the common treatments, however, there are many problems, such as contraindications, drug resistance, toxicity and side effects. Surgical treatment is not only expensive, high risk, but also has higher requirements for the technical level of doctors, and the long-term effect is not necessarily ideal. Traditional Chinese medicine named it "Xiongbi Xintong". ... With the continuous developments of the Department of cardiology in the field of TCM, as well as the characteristics of good therapeutic effect, high safety and cheap price, more patients are willing to use traditional Chinese medicine to treat AP. … ITCWM not only improve the quality of life, but also reduce the frequency of hospitalization and treatment period, common symptoms such as chest pain, palpitation, shortness of breath also rapidly improved. Compared with WM alone, the curative effect is significant.” |
|  | Q8 | Whether the objectives or hypotheses were focused on the ITCWM interventions in the Background? | “Fully reported” was considered if objectives or hypotheses were focused on the ITCWM interventions in the Background. Generally, ITCWM was presented as follows:. i) the word “ITCWM” (similar words) was used; ii) reported as specific names of TCM interventions combined with WM interventions.  “Not reported” was considered if objectives or hypotheses were not focused on the ITCWM interventions.  “Partially reported” was not identified in this item.  “Not applicable” was identified in this item. | 1. “The purpose of this study was to investigate the efficacy of compound Danshen dropping pills combined with Ticagrelor in the treatment of angina pectoris after PCI in patients.” |
| **Methods** | | | | |
| Participants | Q9 | Whether the eligibility criteria of participants included both Chinese and western medical diagnosis in Methods? | “Fully reported” was considered if the eligibility criteria of participants included both TCM and WM diagnosis and provided references.  “Partially reported” was considered if the eligibility criteria of participants included both TCM and WM diagnostic criteria, but no references.  “Not reported” was considered if there was only TCM or WM diagnosis in the eligibility criteria of participants.  “Not applicable” was identified in this item. | 1. “Diagnostic criteria: (1) The WM diagnosis should refer to the 2007 Guidelines for the Diagnosis and Treatment of Chronic Stable Angina Pectoris. (2) The grading of AP was determined according to the CCS grading standard formulated by the Canadian Cardiovascular Society. Qi stagnation and Blood stasis Syndrome differentiation standards refer to the Guiding Principles for Clinical Research on New Chinese Medicines (Trial) (2002 edition) , the Criteria for Diagnosis and Efficacy of TCM Diseases and Syndromes and the second edition of Internal Medicine of Traditional Chinese Medicine. Inclusion criteria: (1) Met the diagnostic criteria for AP, and AP was classified as grade I-ⅲ; (2)Patients with Qi-stagnation and blood-stasis syndrome conforming to TCM syndrome differentiation. |
|  | Q10 | Whether the specific information of disease (e.g., classification of disease, treatment points, stages of diseases) of the ITCWM was reported in Methods? | “Fully reported” was considered if the classification of angina pectoris was provided in Methods.  “Not reported” was considered if there was only “angina pectoris” without any classification.  “Partially reported” was not identified in this item.  “Not applicable” was not identified in this item. | 1. “All the patients … was stable angina pectoris, while the syndrome differentiation of traditional Chinese medicine was liver qi stagnation.” |
| Intervention | Q11 | Whether the specific type/way of integration of TCM and WM interventions (such as overlying, one-after-another, or add-on design) was reported in Methods? | “Fully reported” was considered if the specific type/way of the combination of TCM and WM was reported.  “Not reported” was considered if there was no description of specific way.  “Partially reported” was not identified in this item.  “Not applicable” was not identified in this item. | 1. “Patients were randomly divided into control group and YXR group, respectively. Both control group and YXR group were intervened with routine treatment of western medicine for six successive months, including aspirin enteric-coated tablets (Bayer HealthCare Co., Ltd GYZ Zi J20130078, 100 mg, po, qd) and isosorbide dinitrate tablets (Shanghai Fudan Fuhua Pharmaceutical Co., Ltd GYZ Zi H31021370,10 mg, po, tid). Meanwhile, patients in YXR group were also treated with YXR twice daily in combination with routine western medicine.” |
|  | Q12 | In the ITCWM group, whether TCM intervention(s) was reported with sufficient details to allow replication, including how and when they were administered? | “Fully reported” was considered if sufficient details of TCM interventions in the ITCWM-design group were reported. Generally, the details at least include name, dosage, route of administration, frequency of intervention, and therapeutic period. If the TCM intervention is a formula, it ought to report the name and dosage of each herb included in the formula. If the TCM intervention was acupuncture or other external therapy, it should report the acupoint(s) or specific method.  “Not reported” was considered if there was insufficient information that reader could not repeat the trial followed the direction.  “Partially reported” was not identified in this item.  “Not applicable” was not identified in this item. | 1. “TCM: Jieyu-Buben formula (Granules) (including Duan-Longgu 30g, Duan-Muli 30g, Baizhu 15g, Shudihuang 10g, Huainiuxi 10g, Sangjisheng 10g, dangshen 10g, Baishao 10g, Yujin 10g, Fuzi 6g, Guizhi 6g, Chaihu 6g, and Gancao 3g), oral, one dose per day, twice a day, once in the morning and once in the evening. ” 2. “The experimental group was treated with moxibustion method under the basic drug treatment of the control group, and moxibustion was performed once a day at each point for 30 minutes, 5 times a week, for a total of 20 times. (1) acupoint selection: RN8 (Shenque), bilateral PC6 (Neiguan), bilateral LR3 (Taichong), bilateral SP10 (Xuehai); (2) Position of acupoints: All position of acupoints according to the 2006 National Standard of the People's Republic of China (GBIT12346-2006).” 3. “Patients in the experimental group received both the conventional western treatment and SYD orally at the same time (batch munber: 1902001, specification: 0.4/tables, and producer: Beijing Hospital of Traditional Chinese Medicine Affiliated to Capital Medical University). They took SYD from 3 d before the interventional treatment to 7 d after surgery continuously with 4 capsules 3 times a day. |
|  | Q13 | In the ITCWM group, whether WM intervention(s) was reported with sufficient details to allow replication, including how and when they were administered? | “Fully reported” was considered if sufficient details of WM interventions in the ITCWM group were reported. Generally, the details at least include name, dosage, route of administration, frequency of intervention, and therapeutic period.  “Not reported” was considered if there was insufficient information that reader could not repeat the trial followed the direction.  “Partially reported” was not identified in this item.  “Not applicable” was not identified in this item. | 1. “Observation group: Western medicine: clopidogrel hydrogen sulfate tablets (Lepu Pharmaceutical Co., Ltd., Sinopharm H20123116, specification: 75 mg per tablet, batch number: 8A579), 75mg each time, once a day, orally. Traditional Chinese medicine: the formula of Da Chai Hu Tang ......the above decoctions are provided by the Chinese medicine pharmacy of our hospital. Patients in both groups were treated continuously for 4 weeks.” |
|  | Q14 | In the control group, whether sufficient details were reported to allow replication? | “Fully reported” was considered if sufficient details of interventions in the controlled group were reported, the requirement was as same as the ITCWM group.  “Not reported” was considered if there was insufficient information that reader could not repeat the trial followed the direction.  “Partially reported” was not identified in this item.  “Not applicable” was not identified in this item. | 1. “Controlled group: Provide routine western medicine intervention based on “guidelines for diagnosis and treatment of stable angina Pectoris”: aspirin enteric-coated tablets (Shenyang Ojina Pharmaceutical Co., Ltd., Chinese medicine J20171021), 100mg each time, once a day…” |
| Outcome | Q15 | Whether the outcome measures included both TCM and WM related endpoints in Methods? | “Fully reported” was considered if outcome measures included both TCM and WM related endpoints.  “Not reported” was considered if there was only TCM or WM endpoints.  “Partially reported” was not identified in this item.  “Not applicable” was not identified in this item. | 1. “Outcome Measures: GRACE angina score scale (GRACE), Seattle angina score scale (SAQ) and Angina pectoris symptom grading table of coronary heart disease in Clinical guidelines of traditional Chinese Medicine were used to evaluate the syndromes of AP before and 4 weeks after treatment. GRACE score, SAQ score and TCM symptom score were compared between the two groups before and after treatment.” |
| Blinding | Q16 | For the studies with open label, whether any reasons or explanations for such design was reported? | “Fully reported” was considered if it explained why an open-label trial was designed.  “Not reported” was considered if there was no explanation for open-label design.  “Not applicable” was considered if the study was blinding design or it did not reported whether it was a blinding-design trial.  “Partially reported” was not identified in this item. | 1. “The purpose of this experiment is to observe the efficacy of hospital preparations and listed proprietary Chinese medicines on the basis of routine treatment of western medicine. The number of tablets used is different and the funds are limited, so it did not set the blind in the trial.” 2. “Because the three groups of interventions can be naturally distinguished, it is not possible to blind participants and clinical researchers.” |
| Control | Q17 | In the control group(s), did the placebo of WM invention(s) was included? If so, whether sufficient details were provided? | “Fully reported” was considered if the study reported the WM placebo.  “Not reported” was considered if the trial conducted the placebo design but there was no WM placebo.  “Not applicable” was considered if the trial did not include placebo design.  “Partially reported” was not identified in this item. | 1. No eligible examples for “Fully reporting”. |
|  | Q18 | In the control group(s), did the placebo of TCM invention(s) was included? If so, whether sufficient details were provided? | “Fully reported” was considered if the study reported the TCM placebo.  “Not reported” was considered if the trial had the placebo design but there was no TCM placebo.  “Not applicable” was considered if the trial did not include placebo design.  “Partially reported” was not identified in this item. | 1. “Control group: Xuefu Zhuyu capsule simulation agent (appearance and packaging are the same as and treatment group); Composition: starch, dextrin, edible pigment; Production batch number: 20170301; Production: Tianjin Hongrentang Co., LTD. Packing specification: 0.4 gx12 grains.” |
| **Results** | | | | |
| Baseline | Q19 | In the section of Results, whether any information about the participants exposed to ITCWM treatment prior to recruitment was mentioned in the baseline data? | “Fully reported” was considered if the information about the participants exposed to ITCWM treatment prior to recruitment (restrict to ITCWM interventions) was provided.  “Partially reported” was considered if there was requirment about exposing or not exposing to TCM treatment prior to recruitment.  “Not reported” was considered if there was no description about previous treatment of ITCWM at baseline.  “Not applicable” was not identified in this item. | 1. No eligible examples for “Fully reporting”. |
| **Discussion** | | | | |
|  | Q20 | Whether interpretation and significance of studied ITCWM interventions for the disease was reported in Discussion? | “Fully reported” was considered if the article discussed the interpretations of TCM intervention(s) combined with WM intervention(s) and interpreted the results of ITCWM interventions.  “Partially reported” was considered if the article only reported the efficacy of TCM or WM intervention(s), respectively, or it only reported “ITCWM works better” but without any interpretations.  “Not reported” was considered if there was no description about ITCWM interventions.  “Not applicable” was not identified in this item. | 1. “Clopidogrel hydrosulfate tablets mainly inhibit platelet aggregation, improve clinical symptoms and cardiac function in angina pectoris, resist myocardial oxidation and reduce stress reaction. Clopidogrel sulfate is the main drug in the treatment of anti-platelet aggregation. And it is often used in the treatment of cardiovascular diseases. ……The use of Da Chaihu decoction in patients with angina pectoris can shorten attack time, reduce attack frequency, resist myocardial oxidation, improve cardiac function and promote the recovery of vascular endothelial function.…… Dachaihu decoction combined with clopidogrel sulfate tablets in the treatment of angina pectoris can improve the indexes of cardiac function, regulate the levels of LL-37 and ILT3 in patients with angina pectoris of coronary heart disease, and effectively relieve the inflammatory reaction of patients.” |
| **Other information** | | | | |
| Interests | Q21 | Whether any potential conflicts of interests were clearly reported? | “Fully reported” was considered if the article reported the conflicts of interests; or it declared no interest.  “Partially reported” was considered if the potential conflicts of interests could be found (such as trial sponsor and formula invented people) but the article did not clearly report the potential conflicts of interests.  “Not reported” was considered if the article did not mention any conflicts of interests.  “Not applicable” was not identified in this item. | 1. “Declaration of Competing Interest: The authors declare no competing financial interest.” |

Integrated Traditional Chinese and Western medicine (ITCWM); traditional Chinese medicine (TCM); Western medicine (WM); Not Reported (NR); Not Applicable (NA)

**Supplementary file 4 List of included articles in this study**

1. Zhao L, Li D, Zheng H, Chang X, Cui J, Wang R, Shi J, Fan H, Li Y, Sun X, Zhang F, Wu X, Liang F. Acupuncture as Adjunctive Therapy for Chronic Stable Angina: A Randomized Clinical Trial. JAMA Intern Med. 2019 Oct 1;179(10):1388-1397. doi: 10.1001/jamainternmed.2019.2407. PMID: 31355870; PMCID: PMC6664382.
2. Lyu J, Xue M, Li J, Lyu W, Wen Z, Yao P, Li J, Zhang Y, Gong Y, Xie Y, Chen K, Wang L, Chai Y. Clinical effectiveness and safety of salvia miltiorrhiza depside salt combined with aspirin in patients with stable angina pectoris: A multicenter, pragmatic, randomized controlled trial. Phytomedicine. 2021 Jan;81:153419. doi: 10.1016/j.phymed.2020.153419. Epub 2020 Dec 10. PMID: 33360345.
3. Li, Haide, Xiaoyan Lin, Qingwei Liu and Yang Zhang. Curative effect of Danhong injection on the clinical symptoms, adverse reactions and electrocardiogram of angina pectoris of coronary heart disease. Int J Clin Exp Med 2018;11(2):910-915.
4. Zhang Z, Xing W, Liu H, Zhou Q, Liu X, Shang J. Effects of Shen-Yuan-Dan on Periprocedural Myocardial Injury and the Number of Peripheral Blood Endothelial Progenitor Cells in Patients with Unstable Angina Pectoris Undergoing Elective Percutaneous Coronary Intervention. Evid Based Complement Alternat Med. 2022 Jan 7;2022:9055585. doi: 10.1155/2022/9055585. PMID: 35035512; PMCID: PMC8759927.
5. Li Y, Zhang L, Lv S, Wang X, Zhang J, Tian X, Zhang Y, Chen B, Liu D, Yang J, Dong P, Xu Y, Song Y, Shi J, Li L, Wang X, Han Y. Efficacy and safety of oral Guanxinshutong capsules in patients with stable angina pectoris in China: a prospective, multicenter, double-blind, placebo-controlled, randomized clinical trial. BMC Complement Altern Med. 2019 Dec 11;19(1):363. doi: 10.1186/s12906-019-2778-z. PMID: 31829173; PMCID: PMC6907120.
6. Liu J, Dong Y, Hu X. Efficacy of Yangxin Recipe in Combination with Conventional Western Medicine in Treatment of Angina Pectoris of Coronary Heart Disease. Clin Appl Thromb Hemost. 2022 Jan-Dec;28:10760296221076152. doi: 10.1177/10760296221076152. PMID: 35287480; PMCID: PMC8928372.
7. Ma, Xiaomei, Dehui Yang, Wei Shen, Wei Liang and Qian Lin. Therapeutic effect of Xuezhitong capsule on microvascular angina.” Tropical Journal of Pharmaceutical Research. 2021; 20 (9): 1991-1997.
8. Wu M, Yang S, Liu G, Gu C, Ren P, Zhao R, Zhao Y, Xing Y, Liu L, Liang J. Treating unstable angina with detoxifying and blood-activating formulae: A randomized controlled trial. J Ethnopharmacol. 2021 Dec 5;281:114530. doi: 10.1016/j.jep.2021.114530. Epub 2021 Aug 17. PMID: 34416295.
9. Lan L, Yin T, Tian Z, Lan Y, Sun R, Li Z, Jing M, Wen Q, Li S, Liang F, Zeng F. Acupuncture Modulates the Spontaneous Activity and Functional Connectivity of Calcarine in Patients With Chronic Stable Angina Pectoris. Front Mol Neurosci. 2022 Apr 26;15:842674.
10. 张元贵,李亮,林丰夏,曾志聪,宋银枝.“丹芪散”对冠心病稳定型心绞痛患者的防治作用与机制研究——附30例临床资料[J].江苏中医药,2019,51(10):29-32.
11. 王永年,王士兵.保元汤合桃红四物汤化裁联合美托洛尔辨治气虚血瘀证冠心病心绞痛的临床研究[J].中西医结合心脑血管病杂志,2018,16(18):2652-2656.
12. 付强,齐霁. 谷红注射液辅助治疗冠心病患者PCI术后再发心绞痛的临床疗效及其对炎性因子和血管内皮功能的影响[J]. 医学临床研究,2018,35(1):79-82.
13. 郭聪. “冬病夏治”治疗冠心病稳定型心绞痛的临床观察[D].山东中医药大学,2019.
14. 郭喜平. “理气通腑法自拟方”治疗不稳定型心痛合并便秘（肝郁腑实型）疗效观察[D].北京中医药大学,2020.
15. 赵妍. “宣痹通瘀方”联合运动疗法对冠心病PCI术后胸痛（气滞血瘀证）的临床观察[D].长春中医药大学,2019.
16. 李崇钗. “益气活血，化痰通络”法治疗冠心病心绞痛痰瘀互结证临床观察[D].辽宁中医药大学,2021.
17. 李思维,周亚滨.艾灸联合养心汤治疗冠心病稳定性心绞痛[J].长春中医药大学学报,2021,37(02):322-325.
18. 梁胜波,吴晶晶,陶宇琴.安定汤加味治疗冠心病稳定型心绞痛(气阴两虚兼瘀证)的疗效观察[J].中国中医急症,2021,30(11):1996-1998.
19. 高娟,蒋谷芬,陈芳.八段锦联合五行音乐对稳定性心绞痛伴心肾不交型失眠患者的效果观察[J].中医药导报,2020,26(14):71-75.
20. 丁萌. 八段锦运动应用于心脏康复对冠心病稳定型心绞痛患者疗效的研究[D].山东中医药大学,2021.
21. 李露. 八味通络颗粒治疗瘀血阻络型稳定型心绞痛的临床观察[D].山西省中医药研究院,2018.
22. 叶楠. 保丹通络汤治疗正虚络痹型冠心病稳定型心绞痛的临床观察[D].华北理工大学,2021.
23. 胡黎文. 保元活血颗粒治疗冠心病稳定性心绞痛气虚血瘀证的临床观察[D].湖南中医药大学,2019.
24. 周鹏飞. 保元养心方治疗冠心病稳定性心绞痛（气阴两虚兼血瘀型）的临床试验及机理研究[D].河南中医药大学,2017.
25. 杨月东,王敏健,王新东,沈建平.补本解郁颗粒剂治疗冠心病经皮冠状动脉介入术后脾肾两虚型心绞痛的临床疗效[J].实用心脑肺血管病杂志,2018,26(03):75-78.
26. 陈斌. 补气化浊汤对冠心病稳定型心绞痛（气虚痰阻血瘀证）的临床疗效观察[D]. 陕西中医药大学,2019.
27. 单士润. 补气养阴通脉汤治疗气阴两虚、瘀血阻络型不稳定型心绞痛的临床研究[D].山东中医药大学,2018.
28. 王微. 补肾活血汤治疗冠心病PCI术后再发心绞痛（肾虚血瘀证）的临床研究[D].长春中医药大学,2017.
29. 徐飞. 补肾活血稳压颗粒治疗稳定性心绞痛临床研究[D].江西中医药大学,2019.
30. 何桂莲. 补肾启枢强心颗粒治疗慢性稳定性心绞痛临床研究[D].江西中医药大学,2019.
31. 张仙德. 补阳还五汤化裁治疗冠心病心绞痛的疗效观察及对血清hs-CRP、Hcy的影响[D].黑龙江中医药大学,2020.
32. 郭俊伶. 参柴舒心汤治疗冠心病心绞痛合并焦虑症临床疗效观察[D].黑龙江中医药大学,2017.
33. 许铭娟. 参红通络胶囊治疗冠心病不稳定性心绞痛（气虚血瘀，痰浊阻络证）的临床研究[D].长春中医药大学,2019.
34. 彭星. 参七复脉方治疗气虚血瘀型冠心病稳定型心绞痛的临床研究[D].成都中医药大学,2019.
35. 高鲁. 参七冠心方治疗PCI术后稳定型心绞痛（气虚血瘀证）疗效观察[D].云南中医药大学,2021.
36. 于震寰. 参芪活血方对稳定型心绞痛（气虚血瘀型）患者临床疗效观察及PDW、MPV水平的影响[D].黑龙江中医药大学,2021.
37. 徐莉,王勤.参芪六味汤与基础西药联合治疗稳定型心绞痛临床疗效观察[J].四川中医,2019,37(12):68-71.
38. 韩佶志. 参芪通脉饮对冠心病稳定型心绞痛气虚血瘀证患者疗效的临床观察[D]. 天津中医药大学,2017.
39. 卢雪梅. 参芪养心汤联合常规西医治疗针对冠心病稳定型心绞痛（胸痹-气虚血瘀型）患者的临床疗效观察[D].成都中医药大学,2020.
40. 邹林岑. 参芪逐瘀汤治疗气虚血瘀型原发性稳定型微血管心绞痛的临床疗效观察[D]. 四川:成都中医药大学,2019.
41. 刘羿妍,李浦媛,李晟琰,等. 参芍胶囊治疗冠心病不稳定型心绞痛的临床观察[J]. 中国药房,2017,28(11):1541-1544.
42. 郭英杰,彭筱平,高辉,等. 参芍养心方联合针刺内关穴治疗气阴两虚型稳定型心绞痛的临床研究[J]. 环球中医药,2021,14(4):730-732.
43. 赵红亮,张明轩,张向宇,等. 参松养心胶囊对不稳定型心绞痛伴慢性失眠患者临床疗效的影响[J]. 世界科学技术-中医药现代化,2020,22(2):511-515.
44. 张春蓉. 参松养心胶囊对冠状动脉粥样硬化性心绞痛失眠患者心肌保护作用和睡眠质量的影响[J]. 中医学报,2018,33(2):303-306.
45. 贾思涵. 参元丹对不稳定型心绞痛择期PCI围手术期心肌保护的临床研究及肠道微生态的影响[D].北京中医药大学,2020.
46. 宋艺璇. 参蛭通心胶囊治疗冠心病PCI术后心绞痛气虚血瘀证的临床研究[D].山东中医药大学,2020.
47. 孙玲. 苍矢通络汤治疗冠心病心绞痛合并超重/肥胖痰瘀阻络证的临床观察[D].湖南中医药大学,2019.
48. 陈宇娜. 柴胡解郁汤治疗冠心病稳定型心绞痛（气滞血瘀兼痰浊）伴焦虑状态患者的临床疗效观察[D].黑龙江中医药大学,2019.
49. 蒋泉秀. 柴胡疏肝散合桃红四物汤加味治疗稳定型心绞痛（气滞血瘀证）临床观察[D].成都中医药大学,2021.
50. 陈其林. 柴胡疏肝散加减联合心理干预治疗稳定性心绞痛（肝气郁结证）合并抑郁症的临床疗效观察[D].贵阳中医学院,2018.
51. 徐曼. 柴胡疏肝散加味方治疗稳定型心绞痛（气滞心胸证）的临床研究[D].黑龙江中医药大学,2020.
52. 胡胜南. 柴胡疏肝散治疗冠心病稳定型心绞痛（气滞心胸型）伴焦虑状态的临床疗效观察[D].黑龙江中医药大学,2020.
53. 崔晓燕. 柴胡温胆汤治疗稳定型心绞痛（痰阻心脉证）的临床研究[D].山西中医药大学,2017.
54. 刘雨情. 柴芎舒心汤治疗PCI术后心绞痛气滞血瘀证的临床疗效观察及作用机制研究[D].山东中医药大学,2018.
55. 韩尚晓. 柴枣疏肝化瘀汤治疗稳定型心绞痛（气滞血瘀型）的临床研究[D].山东中医药大学,2020.
56. 李成伟,秦俊岭,刘永娟,等. 菖远汤治疗痰瘀阻络型老年择期经皮冠状动脉介入治疗术后心绞痛80例:一项前瞻性多中心随机对照研究[J]. 中国中西医结合急救杂志,2020,27(1):76-80.
57. 宋海玖,田雅楠,相文阁. 刺五加片对不稳定型心绞痛病人炎性因子及磷酸化Akt的影响[J]. 中西医结合心脑血管病杂志,2020,18(19):3253-3255.
58. 陈铭泰. 从肝论治法治疗冠心病伴焦虑抑郁状态患者的系统评价与临床观察[D].广州中医药大学,2018.
59. 张松峰,郑直,石洪,等. 从脾论治气虚血瘀证不稳定型心绞痛的临床研究[J]. 世界中医药,2017,12(9):2014-2018.
60. 冯薇,栾绍华,郭会敏. 大柴胡汤加减联合硫酸氢氯吡格雷片对冠心病心绞痛患者临床疗效及LL-37、ILT3水平的影响[J]. 药物评价研究,2022,45(2):324-330.
61. 刘艳,朱怡. 大株红景天辅助治疗老年不稳定型心绞痛的疗效及对血清炎症因子的影响[J]. 现代中西医结合杂志,2019,28(34):3836-3839.
62. 吕静静,葛阳涛,马燕楠. 大株红景天治疗不稳定型心绞痛的疗效 及对心肌损伤、血小板活性的影响[J]. 世界中医药,2018,13(9):2193-2195,2199.
63. 朱振宇. 丹参多酚酸盐对不稳定型心绞痛患者经皮冠状动脉介入治疗围手术期心肌损伤及炎性因子的影响[D]. 内蒙古医科大学,2017.
64. 张同乐. 丹参多酚酸盐联合麝香保心丸治疗冠心病心绞痛疗效观察[J]. 陕西中医,2017,38(2):176-177.
65. 张春晓. 丹参红花方治疗冠心病稳定型心绞痛血瘀气滞证的临床疗效及其调控Hippo通路机制的研究[D]. 山东:山东中医药大学,2020.
66. 马欣. 丹参三七方治疗冠心病稳定型心绞痛血瘀气滞证的临床疗效及作用机制研究[D]. 山东:山东中医药大学,2020.
67. 祝婕,董薇,林建华. 丹参酮ⅡA磺酸钠联合尼可地尔治疗冠心病稳定型心绞痛的临床疗效及其对血脂和血清同型半胱氨酸水平的影响[J]. 实用心脑肺血管病杂志,2017,25(2):107-110.
68. 郭斯叶. 丹参饮加减辨证治疗冠心病稳定型心绞痛的临床研究[D].黑龙江中医药大学,2018.
69. 牛芊,邢文龙,刘红旭,王雨桐,朱彦.丹红注射液对不稳定型心绞痛患者围手术期代谢组学的影响[J].世界中医药,2021,16(12):1900-1908.
70. 蒲玉翠.丹红注射液对冠心病不稳定型心绞痛的治疗效果[J].河北医药,2017,39(01):75-77.
71. 刘鹏宇. 丹蒌片治疗痰瘀互结型冠心病稳定型心绞痛的临床研究[D]. 天津中医药大学,2017.
72. 刘艳艳. 丹蒌饮对稳定型心绞痛（痰瘀互结型）患者临床疗效观察及IL-6、IL-17水平的影响[D]. 黑龙江:黑龙江中医药大学,2021.
73. 刘翠霞. 丹七活血汤联合阿托伐他汀对心血瘀阻型冠心病心绞痛病人血脂代谢及血管内皮功能的影响[J]. 中西医结合心脑血管病杂志,2018,16(16):2344-2348.
74. 袁琪旻. 当归四逆汤治疗阴寒凝滞型冠心病不稳定性心绞痛的临床研究[D].长春中医药大学,2020.
75. 张万里. 导痰汤合桂枝茯苓汤加减治疗稳定型心绞痛（痰瘀互结型）的临床观察[D].黑龙江中医药大学,2020.
76. 高红英.灯盏生脉胶囊联合抗血小板药物及他汀治疗不稳定型心绞痛的临床研究[J].海南医学院学报,2018,24(15):1402-1405.
77. 冷亚南. 涤痰汤化裁方治疗冠心病稳定型心绞痛（痰浊内阻证）临床观察[D].黑龙江中医药大学,2020.
78. 张宁,崔瑾,冯麟,孙佳,丁国恒,杨孝芳.电针疗法联合基础药物对慢性稳定性心绞痛患者临床症状的影响[J].中华中医药杂志,2019,34(03):1262-1265.
79. 李迎亚. 调肝健脾通阳方治疗稳定型心绞痛的临床研究[D].云南中医学院,2017.
80. 刘孟. 调脂汤对痰瘀阻络型冠心病心绞痛患者血脂异常达标率的临床疗效观察[D].南京中医药大学,2017.
81. 刘玉.调脂通脉汤联合阿托伐他汀钙对冠心病合并心绞痛患者血脂代谢、血流动力学及血清VCAM-1水平的影响[J].四川中医,2018,36(08):53-56.
82. 胡连霞. 盾叶冠心宁片治疗稳定型心绞痛（气滞血瘀证）的临床研究[D].南京中医药大学,2019.
83. 韩向莉. 耳穴压豆联合痰瘀双解方治疗冠心病（心绞痛）合并抑郁症的卫生技术评估研究[D].山西医科大学,2017.
84. 刘玉.调脂通脉汤联合阿托伐他汀钙对冠心病合并心绞痛患者血脂代谢、血流动力学及血清VCAM-1水平的影响[J].四川中医,2018,36(08):53-56.
85. 曾博斯,杨帆.耳穴压豆配合化痰通脉方治疗冠心病稳定型心绞痛的疗效观察[J].中西医结合心脑血管病杂志,2019,17(22):3557-3560.
86. 杨月,杨倩,张敏.耳穴压籽对心血瘀阻型冠心病不稳定型心绞痛的疗效观察[J].中医药学报,2017,45(01):80-83.
87. 白宇明,黄莉.二陈汤合血府逐瘀汤治疗痰瘀互结型冠状动脉粥样硬化性心脏病心绞痛的临床效果[J]. 中国医药,2019,14(05):659-663.
88. 张祥. 负电场治疗仪治疗冠心病稳定型心绞痛（心血瘀阻证）患者的临床观察[D].成都中医药大学,2018.
89. 李耀征,白保强,马向阳,孙亚勤.复方丹参滴丸对老年冠心病心绞痛sCD40L、Lp-PLA2水平的影响[J].分子诊断与治疗杂志,2022,14(02):317-320+324.
90. 杨晔. 从脾论治法干预冠心病稳定型心绞痛脾虚痰浊证患者最佳适应证的研究[D].辽宁中医药大学,2019.
91. 徐闵,周开梅,游正林,杨启才.复方丹参滴丸联合阿司匹林治疗老年不稳定型心绞痛的临床疗效及其对凝血功能的影响研究[J].实用心脑肺血管病杂志,2017,25(01):160-162.
92. 张晓红.复方丹参滴丸联合苯磺酸左旋氨氯地平治疗冠心病心绞痛的疗效观察[J].现代药物与临床,2018,33(07):1599-1602.
93. 黄春雨,周果,李刚,吴建明,徐雪梅,崔红艳,季燕华,耿桂灵.复方丹参滴丸联合耳穴埋豆及穴位按摩对稳定性心绞痛的干预[J].中国实验方剂学杂志,2017,23(02):175-180.
94. 张晶,周慧瑾.复方丹参滴丸联合氯吡格雷的疗效观察及对冠心病心绞痛患者血清白细胞介素6及白细胞介素10水平影响[J].药物生物技术,2021,28(01):70-73.
95. 董晨艳,陈海燕,李源.复方丹参滴丸联合替格瑞洛治疗冠心病PCI术后心绞痛的临床疗效[J].中西医结合心脑血管病杂志,2022,20(02):292-294.
96. 黄芳,李迎,李科宇,等. 复方丹参滴丸联合辛伐他汀治疗冠心病心绞痛疗效观察[J]. 医学临床研究,2017,34(2):373-375.
97. 白玛央宗,次旦罗布. 复方丹参滴丸联合左卡尼汀治疗不稳定型心绞痛的临床研究[J]. 现代药物与临床,2019,34(6):1641-1646.
98. 康艳生,刘静静,张伟,等. 复方丹参颗粒、复方丹参胶囊、复方丹参片与复方丹参滴丸对冠心病心绞痛患者疗效、炎性因子及氧化应激指标的影响[J]. 药物评价研究,2020,43(2):287-292.
99. 杨芳,段洪涛,童安荣,等. 复方龙血竭胶囊联合曲美他嗪对冠心病稳定型心绞痛心血瘀阻证的疗效及对氧化应激指标水平的影响[J]. 医学临床研究,2019,36(11):2220-2222.
100. 覃玉慧. 复方三七护脉汤治疗冠心病稳定型心绞痛临床观察及对hs-CRP的影响[D].安徽中医药大学,2019.
101. 袁召. 复方三七汤治疗冠心病稳定型心绞痛的临床疗效观察及对脂质代谢的影响[D].安徽中医药大学,2017.
102. 李晶. 复方生脉散治疗冠心病PCI术后再发稳定型心绞痛（气阴两虚，心脉瘀阻证）的临床观察[D].云南中医药大学,2020.
103. 张雪. 复方水蛭散治疗冠心病心绞痛的实验与临床研究[D].延边大学,2018.
104. 裴晓宁.葛兰心宁软胶囊联合富马酸比索洛尔治疗冠心病心绞痛的临床研究[J].中华中医药学刊,2020,38(06):169-172.
105. 廖郁文. 瓜蒌通脉汤治疗痰浊闭阻证冠心病（稳定型心绞痛）的临床观察[D].湖南中医药大学,2021.
106. 王瑞雪. 瓜蒌通脉丸治疗湿热郁阻型心绞痛的临床研究[D].承德医学院,2018.
107. 何蕾.瓜蒌夏苓汤治疗冠心病稳定型心绞痛痰阻心脉证的临床效果[J].中国医药导报,2020,17(21):153-156.
108. 田盼盼. 瓜蒌薤白半夏汤合丹参饮治疗不稳定型心绞痛的临床研究[D].北京中医药大学,2021.
109. 杨帆.瓜蒌薤白半夏汤合涤痰汤加减治疗痰浊闭阻型胸痹心痛临床观察[J].陕西中医,2017,38(02):178-179.
110. 朱瑞,韩影,何勇,张静.瓜蒌薤白半夏汤合血府逐瘀汤加减联合西医治疗胸痹心痛临床研究[J].四川中医,2020,38(11):95-98.
111. 林东. 瓜蒌薤白半夏汤合血府逐瘀汤治疗痰瘀阻络型心绞痛伴高同型半胱氨酸血症的临床研究[D].浙江中医药大学,2019.
112. 单自琴,许剑婕.瓜蒌薤白半夏汤加减治疗冠心病心绞痛痰浊痹阻证对于心肌血运重建的影响[J].世界中医药,2017,12(01):53-56+60.
113. 付虹. 瓜蒌薤白半夏汤加味治疗冠心病心绞痛（胸痹心痛）痰瘀互结证的临床研究[D].长春中医药大学,2017.
114. 周宏伟,孟建宏,张红鸽,张欢,王敏.瓜蒌薤白半夏汤治疗冠状动脉粥样硬化性心脏病心绞痛疗效观察[J].中医学报,2018,33(10):2012-2015.
115. 杨红,杨波,熊秋霞.冠脉宁通方结合美托洛尔治疗冠心病心绞痛疗效及对冠脉斑块的影响[J].陕西中医,2017,38(08):1006-1007.
116. 李国林,姜雪.冠脉宁通方治疗气虚痰瘀互结证冠心病心绞痛效果及对冠状动脉斑块的影响[J].现代中西医结合杂志,2020,29(04):419-422.
117. 朱晓伟. 冠通方治疗冠心病PCI术后心绞痛气虚血瘀证患者的疗效观察[D].广西中医药大学,2020.
118. 骆虹. 冠通方治疗稳定型心绞痛气虚血瘀证的临床观察及对IL-35的影响[D].广西中医药大学,2018.
119. 苗鸿杏. 冠通方治疗PCI术后再发心绞痛的临床观察及对凝血纤溶系统影响[D].广西中医药大学,2019.
120. 干承. 冠通方Ⅱ号治疗老年冠心病（气阴两虚瘀证）的临床研究及对血液流变学的影响[D].广西中医药大学,2020.
121. 廖宗元. 冠通方Ⅱ号治疗气阴两虚型稳定性心绞痛的疗效及对hs-CRP、血脂影响[D].广西中医药大学,2019.
122. 李成龙. 冠通贴辅助治疗冠心病心绞痛临床观察[D]. 陕西中医药大学,2017.
123. 张丹丹,韩安邦,武婧,王倩文,张冰冰,庞博,崔向宁.冠心丹参滴丸对冠心病稳定性心绞痛血瘀证患者炎症因子干预作用的研究[J].世界中西医结合杂志,2018,13(04):509-513.
124. 靳文学,何德英,乔秀兰.冠心颗粒对稳定性心绞痛炎症反应的影响[J].中国实验方剂学杂志,2017,23(12):182-187.
125. 石洁,阿不都吉力力阿不力孜.冠心舒通胶囊治疗冠心病稳定型心绞痛的临床研究[J].中西医结合心脑血管病杂志,2018,16(02):199-201
126. 卜阳阳. 冠心舒治疗痰阻血瘀型稳定型心绞痛的临床观察[D].湖北中医药大学,2018.
127. 赵欣. 冠心汤治疗气虚血瘀型冠心病介入术后残余病变心绞痛的临床疗效研究[D].南京中医药大学,2017.
128. 赵欣,王忠良,刘敏,梁田,张寒梅,王如侠.冠心汤治疗气虚血瘀型冠心病介入术后残余病变心绞痛的临床研究[J].南京中医药大学学报,2020,36(01):14-18.
129. 李同一. 冠心通痹饮对气虚血瘀稳定型心绞痛的临床疗效研究[D].山东中医药大学,2021.
130. 龙阿凤. 冠心通脉胶囊对稳定性心绞痛（气阴两虚、心血瘀阻证）的患者肿瘤坏死因子-α、Hcy的影响[D].湖南中医药大学,2020.
131. 唐晶,姜钧文,肖蕾,等. 冠心通脉贴膏结合西医常规疗法治疗冠心病心绞痛痰瘀互结证临床研究[J]. 国际中医中药杂志,2022,44(3):257-262.
132. 杨佳欣. 冠心香单片治疗PCI术后心绞痛的临床观察[D]. 陕西中医药大学,2017.
133. 黄秋兰. 冠心止痛贴贴敷对冠心病介入治疗术后心绞痛气滞血瘀证的临床观察[D]. 广西中医药大学,2018.
134. 郭娜.汗法治疗阴寒凝滞型不稳定性心绞痛疗效及对患者血浆CRP、ET-1和NO的影响[J].陕西中医,2020,41(04):465-467.
135. 张露露,张其慧.和化宣痹中药穴位贴敷治疗慢性稳定性心绞痛的临床研究[J]. 河北中医,2018,40(01):113-117.
136. 王斌. 荷丹片联合瑞舒伐他汀治疗不稳定型心绞痛患者的疗效观察[D].河北医科大学,2018.
137. 周志雄. 护心康片对微血管心绞痛（气虚痰瘀互阻证）患者Hcy与hs-CRP影响的临床观察[D].湖南中医药大学,2021.
138. 姜晓桐. 护心止痛方治疗经皮冠状动脉支架植入术后心绞痛气滞血瘀证的临床研究[D].山东中医药大学,2021.
139. 王莹威,王静,姜晖.化痰活血方治疗冠心病稳定型心绞痛（痰阻血瘀证）的疗效及对sdLDL-C、hs-CRP的影响[J].中国中医急症,2021,30(06):987-990.
140. 邱元权. 化痰开痹汤对痰阻心脉型胸痹（冠心病稳定型心绞痛）临床疗效观察[D].广西中医药大学,2018.
141. 何春麦. 化痰祛瘀方对改善冠心病稳定型心绞痛（痰瘀互结证）及降低Hcy疗效分析[D].成都中医药大学,2021.
142. 李瑞奕. 化痰祛瘀复方改善痰瘀交阻型稳定性心绞痛患者生活质量临床研究[D].辽宁中医药大学,2019.
143. 杜倩,郑蛟东.化痰祛瘀通脉汤加减治疗冠心病不稳定型心绞痛患者的疗效及其对心肌酶谱、血清基质金属蛋白酶9、可溶性细胞间黏附因子-1水平的影响[J].世界中西医结合杂志,2021,16(12):2255-2260.
144. 朱彬,范忠才.化瘀通脉消痹汤联合自适应运动训练对冠心病心绞痛预后的影响[J].中华中医药学刊,2018,36(06):1478-1481.
145. 张彩凤. 黄连解毒汤合桂枝茯苓汤治疗不稳定性心绞痛（瘀热相搏型）的临床观察[D].黑龙江中医药大学,2020.
146. 常晓雨. 黄连温胆汤合丹参饮加减治疗痰热瘀阻型冠心病不稳定型心绞痛的临床观察[D]. 天津中医药大学,2019.
147. 马旬旬. 黄连枳实薤白桂枝汤对痰阻心脉证冠心病心绞痛患者Hey、SOD、hs-CRP的影响[D].南京中医药大学,2018.
148. 孙凤. 黄龙汤加减方治疗稳定型心绞痛（气虚血瘀型）伴功能性便秘的临床研究[D].山东中医药大学,2019.
149. 李广浩,徐雯婷,曹敏,王佑华,周端,沈琳,赵慧,杨爱玲,樊华,彭珑萍.黄芪保心汤治疗冠心病心绞痛的临床观察[J].上海中医药大学学报,2019,33(01):20-23+41.
150. 赵国良,李达,冯振宇,闫建玲,袁长玲,杨园园.黄芪桂枝五物汤加味治疗冠心病稳定型心绞痛(阳虚脉阻证)的临床研究[J].中西医结合心脑血管病杂志,2019,17(21):3365-3368.
151. 杨园园. 黄芪桂枝五物汤加味治疗稳定型心绞痛阳虚脉阻证的临床疗效观察[D].山西中医药大学,2018.
152. 刘伟伟. 黄芪桂枝五物汤治疗多支冠脉临界病变稳定型心绞痛（气虚血瘀证）的临床研究[D].上海中医药大学,2019.
153. 王于心. 黄芪建中汤合四逆汤加减方治疗冠心病不稳定型心绞痛脾肾阳虚证的临床研究[D].山东中医药大学,2020.
154. 沈洋洋. 豁痰通络饮治疗PCI术后不稳定性心绞痛（痰瘀互结证）的临床研究[D].长春中医药大学,2019.
155. 梁晓鹏,郭彩霞,马杰,陆培培,兰玥,马丽红,华琦,秦建黎,聂如琼,刘淑荣,黄源鹏,侯平,莫云秋,欧阳茂,姜丙华,宋颍民.活心丸(浓缩丸)治疗冠心病稳定性心绞痛的多中心、随机、双盲、安慰剂对照临床研究[J].中国中西医结合杂志,2018,38(03):289-294.
156. 梁晓鹏. 活心丸（浓缩丸）治疗冠心病稳定性心绞痛临床研究[D].北京协和医学院,2018.
157. 张言玉,李益萍,高俊杰,王肖龙.活血化痰方对痰瘀互结型冠心病心绞痛病人血脂及炎症反应的影响[J].中西医结合心脑血管病杂志,2018,16(21):3081-3084.
158. 倪淑宇,王晓丽.活血化痰宁心饮治疗急性冠脉综合征PCI术后心绞痛疗效观察[J].中国中医急症,2020,29(03):507-509.
159. 耿彬,王思洲,苗华为,封亚丽,王艳炜,赵天资.活血化瘀养心通络方辅助治疗冠心病PCI术后心绞痛患者疗效及对炎症因子水平的影响[J].中国医院药学杂志,2020,40(22):2341-2344.
160. 耿彬,苗华为,王思洲,封亚丽,王艳炜,赵天资.活血化瘀养心通络方联合替格瑞洛治疗冠心病经皮冠状动脉介入术术后心绞痛的临床疗效及对血管内皮功能及炎症因子的影响[J].中华中医药学刊,2020,38(11):33-37.
161. 陈倍佳,朱席政.活血化瘀养心通络方治疗PCI术后心绞痛的疗效及对血清炎性因子水平的影响[J].中西医结合心脑血管病杂志,2022,20(11):2057-2061.
162. 刘佳. 活血祛痰汤治疗冠心病稳定型心绞痛（痰瘀互结型）的疗效观察[D].黑龙江中医药大学,2020.
163. 刘小平. 活血通脉安神汤治疗冠心病不稳定型心绞痛伴失眠的临床研究[D].辽宁中医药大学,2019.
164. 张伟,陈蕊.基于“双心同调”理论自拟益气活血通脉方治疗气虚血瘀型稳定型心绞痛[J].中国临床研究,2022,35(03):372-376.
165. 孙靖. 基于“瘀毒”理论的中药虎杖配伍山楂治疗不稳定性心绞痛的临床研究[D].中国中医科学院,2019.
166. 林梅青. 基于“瘀毒”理论探讨桂枝茯苓丸合四妙勇安汤治疗不稳定型心绞痛的临床疗效及对LP-PLA2、hs-CRP的影响[D].福建中医药大学,2021.
167. 刘畅. 基于冠心病稳定性心绞痛（痰瘀互结证）的中医慢病管理服务模式研究[D].长春中医药大学,2020.
168. 孙丽丽. 基于化瘀祛痰、健脾解毒法干预不稳定型心绞痛的临床观察[D].辽宁中医药大学,2017.
169. 韩豪. 基于黄永生教授“瘀能化水”学术思想的稳心Ⅳ号方治疗冠心病痰瘀互结型临床疗效观察[D].长春中医药大学,2020.
170. 颜毓雪. 基于双心模式评价柴胡疏肝散加味对冠心病稳定型心绞痛患者临床疗效的影响[D].黑龙江中医药大学,2020.
171. 许关振. 基于体质辨识对冠心病稳定型心绞痛病人饮食干预方案的临床观察[D].长春中医药大学,2019.
172. 魏瑞丰,牛志健,马学芹,史乐辰,张晨璐,李永春.基于通补宗气法选穴针刺对慢性稳定型心绞痛患者血管活性因子的影响——附32例临床资料[J].江苏中医药,2020,52(07):57-60.
173. 刘洋. 基于子午流注理论观察血府逐瘀汤治疗不稳定型心绞痛（心血瘀阻型）的临床疗效[D].黑龙江中医药大学,2019.
174. 李诗琪. 加减瓜蒌薤白半夏汤治疗不稳定型心绞痛痰瘀互结证的疗效评价[D].辽宁中医药大学,2021.
175. 滕军. 加味八珍颗粒治疗冠状动脉粥样硬化性心脏病心绞痛气虚血瘀型的临床研究[D].青岛大学,2017.
176. 李倩. 加味补阳还五汤治疗气虚血瘀型糖尿病合并冠心病稳定型心绞痛的研究[D].山东中医药大学,2019.
177. 陈丽. 加味丹葛止痛方对冠心病PCI术后心绞痛的临床疗效评价[D].广西中医药大学,2017.
178. 杜红,雷锐,张鸿雁,牟红媛,游媛媛.加味当归四逆汤治疗冠心病不稳定型心绞痛（阴寒凝滞证）的临床观察[J].中国中医急症,2022,31(05):875-877.
179. 贾晓雅. 加味当归四逆汤治疗冠心病稳定型心绞痛阴寒凝滞证的临床观察[D].湖南中医药大学,2020.
180. 王萍. 加味瓜蒌薤白半夏汤治疗冠心病PCI术后心绞痛患者的临床研究[D].广西中医药大学,2021.
181. 龙臣. 加味瓜蒌薤白半夏汤治疗气虚痰瘀型胸痹（稳定型心绞痛）的临床观察[D].湖北中医药大学,2019.
182. 李伟. 加味瓜蒌薤白半夏汤治疗稳定性心绞痛的疗效观察及对hs-CRP的影响[D].华北理工大学,2018.
183. 王璐. 加味身痛逐瘀汤治疗稳定型心绞痛心血瘀阻证的临床观察[D]. 河北北方学院,2021.
184. 林露. 加味生脉散合丹参饮治疗冠心病PCI术后再发气阴两虚，心血瘀阻型心绞痛的临床观察[D].湖南中医药大学,2020.
185. 魏星,吴伟胜.加味温胆汤对稳定型心绞痛Hs-CRP和血脂的影响[J].陕西中医,2017,38(03):275-276+279.
186. 孙铭鸿. 加味温胆汤治疗痰瘀内阻型冠心病稳定型心绞痛伴焦虑状态临床观察[D].辽宁中医药大学,2018.
187. 吴逸姣. 加味枳实薤白桂枝汤治疗PCI术后心绞痛痰瘀互结证的临床观察[D].南京中医药大学,2020.
188. 杨霞,邹景霞,娄伦田,苏凯.健脾降浊护心汤在冠心病心绞痛的治疗中的应用价值[J].世界中医药,2022,17(08):1118-1121.
189. 冯玲云. 降脂通脉丸治疗稳定型心绞痛（痰瘀互结证）的疗效观察[D].山西中医药大学,2019.
190. 吴娟,程丑夫.降脂消斑片辅助西医常规治疗对冠心病心绞痛冠脉斑块和炎症因子的影响[J].中国中医药信息杂志,2018,25(03):25-29.
191. 高亚光. 解郁定痛汤治疗气滞血瘀型稳定型心绞痛的临床研究[D].山东中医药大学,2018.
192. 刘彬. 灸法对慢性稳定型心绞痛（气滞血瘀证）患者增效作用的临床观察[D]. 贵州:贵阳中医学院,2017.
193. 张阳. 橘枳姜汤治疗冠心病不稳定型心绞痛（气滞痰凝证）的临床观察[D].山西中医药大学,2018.
194. 冯孟华. 开胸通痹汤治疗痰阻血瘀型冠心病不稳定性心绞痛的临床研究[D].华北理工大学,2018.
195. 丁怀莹,王保和.开郁祛痰活血汤治疗冠心病心绞痛合并抑郁症[J].吉林中医药,2019,39(09):1169-1172.
196. 杨师午. 抗心绞痛方治疗冠心病稳定性心绞痛（气滞血瘀证）临床研究[D].湖北中医药大学,2021.
197. 熊璐. 抗胸痛合剂治疗冠心病稳定性心绞痛（气滞血瘀证）的临床研究[D].湖北中医药大学,2019.
198. 衣美霞. 宽胸化痰汤治疗老年稳定型心绞痛痰浊闭阻证的临床观察[D].云南中医药大学,2020.
199. 方金燕,冯月红,王佳薇,陈方慧,章浩,徐远胜,沈国英,王弋.宽胸气雾剂联合常规疗法治疗寒凝血瘀型冠心病稳定性心绞痛随机对照研究[J].中国中西医结合杂志,2022,42(02):172-175.
200. 樊曼璐. 宽胸通络饮治疗慢性稳定性心绞痛痰瘀阻络证的临床研究[D]. 山东:山东中医药大学,2018.
201. 徐娟. 蓝苓降脂方治疗稳定型心绞痛脾虚痰瘀阻滞证的临床观察[D].湖南中医药大学,2018.
202. 李惠莹.理气活血滴丸联合尼可地尔对心脏微血管型心绞痛运动平板试验、炎性反应、血管内皮功能的影响[J].现代中西医结合杂志,2019,28(10):1096-1099.
203. 李艳,赵洋,郑铁钢,张苗.理气活血滴丸治疗气虚血瘀型冠心病心绞痛疗效及对血液流变学和炎症因子的影响[J].现代中西医结合杂志,2020,29(25):2820-2823.
204. 娄扬. 理气活血方治疗冠心病不稳定型心绞痛的临床研究[D].河北医科大学,2018.
205. 王新冰. 理气解郁汤治疗冠心病稳定型心绞痛合并焦虑抑郁的疗效研究[D]. 陕西中医药大学,2018.
206. 李丹. 理气止痛汤治疗冠心病PCI术后气滞血瘀型心绞痛的临床研究[D]. 山西中医药大学,2021.
207. 马雪松. 莲子心煎剂对不稳定型心绞痛患者MMP-9、TIMP-1水平及生存质量影响[J]. 辽宁中医药大学学报,2018,20(8):5-8.
208. 苏明霞,刘宁,李雯. 苓桂术甘汤治疗不稳定型心绞痛临床研究[J]. 中医学报,2017,32(4):631-634.
209. 李彬世. 六君丹参颗粒治疗气虚痰瘀型稳定型心绞痛的临床研究[D]. 广东:广州中医药大学,2019.
210. 景国际,杨然,刘大伟,等. 络风宁1号方治疗冠心病不稳定性心绞痛临床疗效及其机制探讨[J]. 北京中医药,2017,36(1):82-84.
211. 邓杰. 埋针配合基础治疗对稳定型心绞痛的临床疗效分析[D]. 湖北:湖北中医药大学,2018.
212. 党林林. 蒙药当贡-3胶囊对稳定型心绞痛患者血脂及血小板活化因子影响的临床研究[D]. 内蒙古医科大学,2021.
213. 田同亮. 蒙药三味檀香散治疗不稳定型心绞痛的临床疗效观察[D]. 内蒙古:内蒙古民族大学,2018.
214. 李雪磊. 南芪肝着汤治疗冠脉支架术后气虚血瘀型稳定型心绞痛的临床观察[D]. 河北北方学院,2020.
215. 王禹,金丽,杨丹凤,等. 尼可地尔联合参芍胶囊治疗不稳定型心绞痛的临床研究[J]. 中国循证心血管医学杂志,2019,11(2):219-222.
216. 刘怡. 宁心和胃方佐治不稳定型心绞痛的疗效观察[D]. 山东:山东中医药大学,2021.
217. 何德英,张秋,孙文,邹演梅,赵磊,陈红梅,廖昕悦,杨红霞.宁心通痹汤联合穴位贴敷治疗冠心病不稳定型心绞痛临床观察[J].中国中医急症,2020,29(03):504-507.
218. 郑九操,姚勇,张明勇.蒲氏双和散联合西药应用于慢性稳定型心绞痛治疗的临床观察[J].世界中西医结合杂志,2021,16(01):176-180.
219. 徐燕,杨月东,王新东,沈建平.芪参护心方治疗冠心病经皮冠状动脉介入治疗术后气虚血瘀型心绞痛30例[J].安徽中医药大学学报,2018,37(02):25-29.
220. 张媛媛,周晓俊,康启.芪参通冠汤辅助治疗冠心病稳定性心绞痛对患者血脂及血清tPAI-1、sICAM-1、MMP-9水平的影响[J].四川中医,2019,37(12):62-65.
221. 侯凯峰. 芪参通冠汤治疗冠心病稳定性心绞痛（气阴两虚兼血瘀证）临床疗效观察[D].黑龙江中医药大学,2017.
222. 林晓慧. 芪参通络饮对稳定型心绞痛（气虚血瘀型）患者临床疗效的观察及血清MMP-9、sICAM-1水平的影响[D].黑龙江中医药大学,2019.
223. 牛蕊. 芪参通络饮对稳定型心绞痛（气虚血瘀型）患者临床疗效观察及HCY、脂联素水平的影响[D].黑龙江中医药大学,2020.
224. 李亭慧. 芪参通络饮对稳定型心绞痛患者（气虚血瘀型）运动耐量及血清SOD和MDA水平的影响[D].黑龙江中医药大学,2017.
225. 王洋. 芪参通络饮对稳定型心绞痛患者（气虚血瘀型）临床疗效观察及对血清GSH-Px、LPO的影响[D].黑龙江中医药大学,2017.
226. 王昆. 芪参益气滴丸对冠心病PCI术后患者（气虚血瘀型）心功能及心绞痛疗效观察[D].安徽中医药大学,2019.
227. 许祖建,戴小华,汤丽芬,黄宏烨,毛冬梅,周鹏.芪参益气滴丸对冠心病PCI术后患者心功能及心绞痛疗效的影响[J].时珍国医国药,2019,30(09):2208-2209.
228. 许吉. 芪参益气滴丸对经皮冠脉介入治疗的老年不稳定性心绞痛患者预后的影响[D].苏州大学,2019.
229. 王越. 芪参益气滴丸干预急性冠脉综合征PCI术后心绞痛患者（气虚血瘀型）的临床观察[D].山东中医药大学,2020.
230. 刘敏. 芪参益气滴丸联合心脉通贴散对不稳定型心绞痛PCI术后心绞痛的临床研究及对炎症因子的影响[D].南京中医药大学,2017.
231. 李欣欣. 芪归通心汤治疗PCI术后心绞痛的临床研究[D].长春中医药大学,2019.
232. 赵刚峡,王水平,王长城,许宝兴.芪苈强心胶囊联合尼可地尔治疗冠心病心绞痛的临床研究[J].现代药物与临床,2018,33(07):1583-1587.
233. 牛平平,陈小光,李松,吴焕林,陈佳.芪苈强心胶囊治疗气虚血瘀型冠心病心绞痛的临床疗效及机制[J].现代生物医学进展,2018,18(14):2701-2704+2695.
234. 梁文华,周炜,石纠纠,肖莎莎,赵洋.芪蛭三七汤治疗气虚痰瘀阻络型不稳定型心绞痛患者的疗效及作用机制研究[J].现代生物医学进展,2020,20(12):2396-2400.
235. 谢慧敏. 杞地养阴止痛颗粒对阴虚气滞型冠心病不稳定性心绞痛的临床疗效研究[D].长春中医药大学,2019.
236. 李弘毅. 清胆泻火汤治疗冠心病不稳定型心绞痛胆热扰心证的临床研究[D].山东中医药大学,2021.
237. 陈胤峰,何庆勇,刘超,王阶.清热活血方剂干预冠心病心绞痛瘀热互结证临床观察[J].中华中医药杂志,2017,32(09):4288-4291.
238. 邵英强. 清热解毒、活血祛瘀法治疗不稳定型心绞痛热毒血瘀型临床疗效观察[D].山东中医药大学,2017.
239. 王雨婷. 清热解毒化瘀法治疗热毒瘀阻型不稳定型心绞痛的临床研究[D].山东中医药大学,2020.
240. 张克清,戴珍,贾海莲.祛瘀化痰宁心汤联合西药治疗急性冠脉综合征经皮冠状动脉介入术后心绞痛45例临床观察[J].中医杂志,2018,59(22):1943-1947.
241. 李祎楠,刘欣艳.祛瘀化痰汤联合针刺治疗痰浊痹阻型冠心病心绞痛发作期的临床观察[J].河北中医,2019,41(11):1712-1716.
242. 冯浩丽.人参合桃红四物汤联合瑞舒伐他汀对冠心病心绞痛病人心功能及血脂的影响[J].中西医结合心脑血管病杂志,2018,16(21):3177-3179.
243. 薛思杨. 人参四物汤治疗心脏微血管性心绞痛的临床研究[D].山东中医药大学,2020.
244. 任毅,沈怡,祝海毅,何德英,李勇,曹晋,柏哲.三黄稳心汤改善冠心病心绞痛冠脉介入术后患者生存质量的临床研究[J].中国中医急症,2019,28(12):2160-2162.
245. 万思琦. 三七红参粉治疗稳定型心绞痛（心血瘀阻证）的疗效观察[D].黑龙江中医药大学,2020.
246. 于伟宏. 散结通脉方对痰瘀互结型冠心病不稳定性心绞痛的临床疗效研究[D].长春中医药大学,2018.
247. 贺明清,陈小钦,谢秩芬.麝香保心丸联合单硝酸异山梨酯缓释片治疗冠心病心绞痛的疗效观察[J].现代药物与临床,2018,33(07):1608-1612.
248. 罗洪民,王湛贤,江杏娟,陆品刚.麝香保心丸联合热敏灸治疗冠心病心绞痛临床研究[J].南京中医药大学学报,2019,35(04):391-394.
249. 戴雅琴,童欢,程冰洁.麝香保心丸联合辛伐他汀对冠心病心绞痛患者血清IL-18、VEGF、T-SOD水平的影响[J].中华中医药学刊,2022,40(01):188-192.
250. 刘士福,张文勇.麝香保心丸联合异山梨酯治疗不稳定型心绞痛的疗效及安全性分析[J].药物评价研究,2017,40(10):1445-1448.
251. 陈德智. 理气活血滴丸治疗冠心病慢性稳定型心绞痛（气滞血瘀、胸阳痹阻证）的临床疗效观察[D].山东中医药大学,2018.
252. 李焕. 升陷汤加味治疗气虚血瘀型冠心病稳定型心绞痛的临床研究[D].长春中医药大学,2019.
253. 牟园园. 生柴汤治疗肝郁气滞型稳定型心绞痛的临床研究[D].山东中医药大学,2019.
254. 余海英. 生脉冠心方治疗气虚血瘀型慢性稳定性心绞痛的临床观察[D].云南中医药大学,2019.
255. 刘兴林. 生脉活血方治疗冠心病稳定型心绞痛（气阴两虚兼血瘀证）的临床观察[D]. 陕西中医药大学,2020.
256. 张伟杰,贺卫超,张旭杰. 生脉活血汤加减对不稳定型心绞痛患者血清碱性成纤维细胞生长因子和血小板α-颗粒膜蛋白140水平的影响[J]. 中国医药,2021,16(3):331-335.
257. 任盼宁. 生脉养血保心汤治疗冠心病稳定型心绞痛（气阴两虚兼瘀型）的临床研究[D]. 山东:山东中医药大学,2019.
258. 崔嘉妍. 史载祥教授升陷祛瘀法治疗难治性心绞痛的临床研究[D]. 广东:广州中医药大学,2018.
259. 何美娟. 疏肝健脾调脂颗粒治疗稳定性心绞痛合并焦虑状态的临床研究[D].江西中医药大学,2019.
260. 卓志芳. 疏肝理气活血法治疗稳定型心绞痛气滞血瘀证患者的临床研究[D].广州中医药大学,2019.
261. 卓志芳,靳利利,史振羽,袁丁,苏慧,祁钰涵,王丽莹.疏肝理气活血方治疗冠心病心绞痛患者临床疗效观察[J].辽宁中医杂志,2020,47(07):90-94.
262. 陈丹丹. 疏肝宁心方治疗肝经瘀热型冠心病稳定型心绞痛的临床观察[D]. 河北北方学院,2021.
263. 温伟. 舒血宁注射液治疗心血瘀阻型不稳定型心绞痛患者的临床观察及对hs-CRP的影响[D]. 黑龙江:黑龙江中医药大学,2021.
264. 董竞方,马晓昌. 双参宁心颗粒治疗气虚血瘀型稳定型心绞痛疗效观察[J]. 西部中医药,2019,32(1):70-73.
265. 姜浩. 双丹颗粒治疗冠心病PCI术后心绞痛（心血瘀阻证）的临床研究[D]. 陕西中医药大学,2017.
266. 张富国. 水蛭通络胶囊治疗冠心病PCI术后再发不稳定型心绞痛（气虚血瘀型）的临床研究[D]. 河北北方学院,2021.
267. 程圣哲. 四参护心汤提高PCI后气虚血瘀型心绞痛患者生存质量的临床观察[D].北京中医药大学,2019.
268. 金莲玉. 四红止痛汤治疗不稳定型心绞痛（气阴两虚夹瘀型）的临床研究[D].长春中医药大学,2018.
269. 姜红岩. 太极拳对稳定性心绞痛患者心肺功能及生活质量的影响[D].中国中医科学院,2018.
270. 刘梦雪. 太极拳干预对慢性稳定性心绞痛临床疗效研究[D].成都中医药大学,2018.
271. 杨旭. 痰浊闭阻型不稳定型心绞痛源流及临床研究[D]. 天津中医药大学,2017.
272. 袁利梅,范立华,张志国,李庆海.桃红四物汤合柴胡疏肝散加减治疗不稳定型心绞痛A型行为的临床观察[J].中国实验方剂学杂志,2019,25(18):89-94.
273. 罗洋,庞建中.桃仁红花煎治疗劳力性心绞痛心血瘀阻证疗效观察[J].现代中西医结合杂志,2020,29(07):749-752.
274. 钱坤. 体外反搏技术联合中西医治疗不稳定型心绞痛及对NO、MCP-1的影响[D].辽宁中医药大学,2020.
275. 尹金秀.体外反搏联合益气化瘀方治疗冠心病稳定型心绞痛效果观察[J].现代中西医结合杂志,2021,30(25):2803-2807.
276. 宫淑颖. 体外反搏联合中西医治疗PCI术后心绞痛的临床研究及其机制探讨[D].辽宁中医药大学,2018.
277. 马常媛. 体外反搏治疗冠心病不稳定型心绞痛（气虚挟痰瘀证）的临床研究[D].长春中医药大学,2018.
278. 邹驰. 天麻素抗缺氧功效研究及其缓释剂治疗老年冠心病心绞痛的临床观察[D].湖北中医药大学,2018.
279. 周婷. 天香丹治疗冠心病稳定型心绞痛的临床研究[D].新疆医科大学,2017.
280. 周飞,徐海丽,任伟,陶亚丽.通魂逆心汤治疗血瘀型冠心病稳定型心绞痛[J].中医学报,2020,35(08):1779-1783.
281. 马丽君. 通脉活络膏治疗冠心病稳定型心绞痛（痰瘀交阻证）临床疗效研究[D]. 陕西中医药大学,2017.
282. 刘红伟,刘艳梅. 通脉降浊方治疗冠心病心绞痛合并高脂血症[J]. 吉林中医药,2021,41(9):1184-1188.
283. 张璐瑶. 通脉解郁汤治疗冠心病稳定型心绞痛（心肝郁滞型）的临床疗效观察[D]. 华北理工大学,2021.
284. 吴永胜. 通脉汤治疗气虚血瘀型稳定型心绞痛的临床观察[D].湖南中医药大学,2020.
285. 李杨晓. 通脉益气汤治疗气虚痰瘀型冠心病稳定型心绞痛的临床研究[D].华北理工大学,2021.
286. 胡刚. 通心痹合剂治疗冠心病心绞痛气阴两虚、痰瘀热阻证的临床研究[D].扬州大学,2018.
287. 张磊,李会芳,刘红梅,杨海艳,任焱,冯永生,冯胜红,周维.通心颗粒治疗冠心病心绞痛(气虚血瘀证)临床观察[J].四川中医,2021,39(07):91-94.
288. 张铁. 通心络胶囊对冠心病稳定型心绞痛患者（气虚血瘀证）血清瘦素的影响[D]. 陕西中医药大学,2017.
289. 陈小卫,黄志文,田丽红,等. 通心络胶囊对冠心病PCI术后心绞痛患者的疗效及炎性反应和血管内皮功能的影响[J]. 中国新药杂志,2017,26(20):2459-2462.
290. 王芳,陈洁,刘宇涵,等. 通心络胶囊联合倍他乐克对冠心病稳定型心绞痛患者心功能的影响[J]. 世界中医药,2021,16(17):2605-2610.
291. 南明花,焦晓民,李爽,等. 通心络胶囊治疗气虚络瘀型不稳定型心绞痛有效性和安全性分析[J]. 辽宁中医药大学学报,2018,20(6):156-159.
292. 贲峰. 通心络联合曲美他嗪改善冠心病心绞痛患者心功能及病情效果研究[J]. 解放军预防医学杂志,2018,36(12):1532-1534.
293. 郑浩龙. 通阳宽胸颗粒对稳定型心绞痛患者临床疗效及血清VEGF的影响[D].广州中医药大学,2018.
294. 郑浩龙,靳利利. 通阳宽胸颗粒配合西医常规疗法治疗气滞痰瘀证稳定型心绞痛临床研究[J]. 国际中医中药杂志,2018,40(2):103-106.
295. 杜晓云. 通阳泄浊法治疗冠心病稳定型心绞痛痰浊闭阻证的临床疗效观察[D]. 山东:山东中医药大学,2019.
296. 林浩. 通阳宣痹汤治疗痰瘀互结型稳定型心绞痛的临床研究[D]. 广西中医药大学,2020.
297. JINGHUA WANG. 温胆汤合四物汤加味治疗冠心病稳定型心绞痛（痰瘀互结型）的临床研究[D]. 山东:山东中医药大学,2018.
298. 续自凤. 温经通络法治疗稳定型心绞痛（寒凝心脉型）的临床研究及对Lp-PLA_2的影响[D].山东中医药大学,2019.
299. 李培蕾,张富生. 温阳化痰疏肝汤配合瑞舒伐他汀对冠心病心绞痛患者血液流变学、血脂的影响[J]. 四川中医,2020,38(1):81-83.
300. 延秀敏,樊瑞红.温阳活血法内外同治阳虚血瘀型不稳定型心绞痛70例临床观察[J].中医杂志,2017,58(19):1666-1670.
301. 于长生,宫丽鸿,赵殿臣.稳斑汤联合体外反搏治疗冠心病PCI术后心绞痛临床疗效及对血清ET-1和NO影响[J].辽宁中医药大学学报,2020,22(02):150-153.
302. 杜思达. 五参养心汤治疗冠心病稳定型心绞痛（气虚血瘀型）的临床观察[D].黑龙江中医药大学,2020.
303. 刘勇,刘超峰.陷胸逐瘀汤治疗痰瘀毒互结型冠心病心绞痛疗效及对心肌耗氧量的影响[J].现代中西医结合杂志,2020,29(02):158-162.
304. 张凯泰. 香丹通络止痛方治疗不稳定型心绞痛（气滞血瘀证）的临床观察研究[D].长春中医药大学,2020.
305. 杨莉霞. 消溶稳斑方对不稳定型心绞痛的疗效观察及血清炎症因子MCP-1、TNF-α的影响[D].河南中医药大学,2018.
306. 布天瑞. 消溶稳斑方对不稳定型心绞痛斑块的作用及TNF-α、hs-CRP、AMPK的影响[D].河南中医药大学,2018.
307. 牛佳丽. 消溶稳斑方对不稳定型心绞痛斑块的作用及血清网膜素-1、脂联素、TNF-α水平的影响[D]. 河南中医药大学,2019.
308. 张华敏. 消溶稳斑方对不稳定型心绞痛易损斑块的作用及血清MIF、Vaspin、IL-10的影响[D]. 河南中医药大学,2019.
309. 杜雨楠. 消溶稳斑方治疗不稳定型心绞痛及对血清炎症因子IL-18、TGF-β1水平的影响[D]. 河南中医药大学,2017.
310. 乔培. 消溶稳斑方治疗不稳定性心绞痛及对血清炎症因子MMP-9、hs-CRP影响的研究[D]. 河南中医药大学,2017.
311. 张学新,刘艳军,李权. 小柴胡汤加减治疗经皮冠状动脉介入治疗后心绞痛气郁血瘀证的临床疗效及对血脂、血液流变学的影响[J]. 河北中医,2020,42(7):990-994.
312. 徐佳萌,郑刚,任耀龙,齐婧,杨聪,王艳琴.小陷胸汤合丹参饮加味联合西药治疗痰瘀互结型稳定型心绞痛合并H型高血压40例临床观察[J].中医杂志,2020,61(17):1541-1546.
313. 周华东. 心达康滴丸治疗冠心病稳定性心绞痛（痰瘀互结型）的临床疗效观察及对Hcy、hs-CPR、血脂的影响[D].安徽中医药大学,2021.
314. 刘玉金,李彩云,吴艳松,王志勇,李运超,孙永辉,贾振华.心肺同治干预慢性阻塞性肺疾病稳定期合并稳定性心绞痛24例临床研究[J].江苏中医药,2019,51(02):36-39.
315. 朱贺,周鹏飞,白倩,袁智宇.心复康胶囊治疗冠心病稳定性心绞痛气虚血瘀证临床研究[J].中医学报,2017,32(06):1021-1024.
316. 贾静涛. 心可舒片对稳定型心绞痛（气滞或血瘀型）临床症状及生活质量的短期干预研究[D].新乡医学院,2018.
317. 辛衍璞. 心脑舒通胶囊治疗稳定型心绞痛（心血瘀阻证）的临床观察[D].黑龙江中医药大学,2017.
318. 刘夏清. 心脑通片治疗冠心病稳定性心绞痛（瘀血阻滞证）临床研究[D].湖北中医药大学,2017.
319. 费鸿翔,徐瑛,韩天雄.心脾同治方治疗气虚血瘀型冠心病稳定型心绞痛临床观察[J].上海中医药大学学报,2017,31(04):20-23.
320. 黎炯彤. 心痛宁方对气虚血瘀型冠心病患者PCI术后心绞痛的疗效观察[D].广西中医药大学,2021.
321. 伍瑶. 心痛宁膏治疗冠心病PCI术后心绞痛气虚痰瘀证的临床观察[D].湖南中医药大学,2020.
322. 代璐. 心痛宁膏治疗心肌梗死后心绞痛气虚痰瘀互结证的临床观察[D].湖南中医药大学,2021.
323. 张超,关敬树,姚成增,李晓惠,周云,缪志静,高山钟,陈佳,陆伟,李悦.心痛宁颗粒联合常规治疗对气虚痰瘀型稳定性心绞痛患者的临床疗效[J].中成药,2021,43(11):3014-3018.
324. 李佳玲. 心痛泰治疗稳定性心绞痛气滞血瘀证的疗效及心肌能量代谢的临床研究[D].湖南中医药大学,2021.
325. 杨晓利. 心痛硬热膏穴位贴敷治疗冠心病稳定型心绞痛（气滞血瘀证）的疗效观察[D].河南中医药大学,2018.
326. 谢磊,李必泽,毕文明,张鹏,李明.心元胶囊对不稳定型心绞痛疗效及对中医证候评分、生存质量的影响[J].中国中医急症,2020,29(08):1464-1467.
327. 王天罡. 行气活血方治疗冠心病不稳定型心绞痛（气滞血瘀证）的临床观察[D].辽宁中医药大学,2017.
328. 邵婷婷. 行气活血汤治疗PCI术后稳定性心绞痛的临床疗效观察[D].辽宁中医药大学,2020.
329. 刘瑞. 胸痹汤对冠心病不稳定型心绞痛（气虚血瘀证）患者hs-CRP、NT-proBNP、纤维蛋白原的影响及临床研究[D].安徽中医药大学,2018.
330. 周小芳. 胸痹汤对冠心病不稳定型心绞痛气虚血瘀证患者Fractalkine、hs-CRP、血液流变学的影响及临床研究[D].安徽中医药大学,2019.
331. 谢晚亭. 胸痹汤对冠心病不稳定型心绞痛气虚血瘀型患者ox-LDL、HCY、hs-CRP、ECG的影响及临床研究[D].安徽中医药大学,2017.
332. 宗营. 胸痹汤治疗气虚痰瘀互结型冠心病不稳型心绞痛的临床疗效观察及对Hcy、hs-CRP、NT-pro BNP的影响[D].安徽中医药大学,2021.
333. 王珊. 胸痹心痛膏治疗冠心病稳定型心绞痛（痰阻血瘀证）的临床疗效观察[D].甘肃中医药大学,2018.
334. 魏群,魏晏,魏明.宣痹消痛汤治疗冠心病心绞痛临床研究[J].时珍国医国药,2018,29(08):1920-1922.
335. 郑庆扬,廖梅,韩钰畴,高淑铮.宣痹益气通阳方辅助西药治疗不稳定型心绞痛疗效观察[J].中国中医急症,2020,29(09):1631-1633.
336. 王远玲,胡清甫,高健敏.穴位按压联合硝酸甘油含服治疗冠心病急性心绞痛疗效观察[J].上海针灸杂志,2020,39(05):521-525.
337. 贯淼.穴位贴敷联合西药治疗气滞血瘀型冠心病心绞痛疗效观察[J].上海针灸杂志,2019,38(01):21-24.
338. 王贺,周亚滨.穴位贴敷疗法治疗冠心病不稳定性心绞痛气虚血瘀型临床观察[J].辽宁中医药大学学报,2017,19(04):109-111.
339. 任小娟,祝婕,张秀芬,帕丽旦,赵金娜,王卫.雪莲通脉丸对冠心病心绞痛患者血管内皮影响的临床研究[J].世界中医药,2017,12(09):1993-1996.
340. 胡坤. 血府逐瘀胶囊干预稳定型心绞痛气滞血瘀证患者的circRNA临床相关机制研究[D].中国中医科学院,2020.
341. 房美. 血府逐瘀汤加减方治疗稳定型心绞痛（气滞血瘀型）伴焦虑状态的临床研究[D].山东中医药大学,2019.
342. 赵佳. 血府逐瘀汤加酒大黄治疗气滞血瘀型不稳定型心绞痛的临床研究[D].山东中医药大学,2020.
343. 洪天一. 血府逐瘀汤联合西药治疗冠心病心绞痛的临床研究[D]. 吉林:延边大学,2017.
344. 王晨希,孙伯青. 血府逐瘀汤治疗冠心病稳定型心绞痛伴高同型半胱氨酸血症[J]. 吉林中医药,2017,37(7):689-692.
345. 何涛,易桂文.血脂康胶囊联合舒脉汤对伴有冠状动脉非左主干临界病变的稳定型心绞痛患者血脂指标、hs-CRP、FIB、TNF-α影响[J].现代中西医结合杂志,2019,28(18):1969-1974.
346. 费鸿翔,范俊飞,张翔宇,颜乾麟,韩天雄.颜氏益心汤改良方治疗冠心病稳定性心绞痛气虚血瘀证临床观察[J].河北中医,2022,44(04):554-558.
347. 谢皛,褚福永.养心定悸胶囊对不稳定型心绞痛冠脉介入术后病人室性早搏及心率变异性的影响[J].中西医结合心脑血管病杂志,2017,15(04):448-451.
348. 张倩倩. 养心化瘀汤治疗冠心病不稳定型心绞痛气虚血瘀证的临床观察[D].湖南中医药大学,2021.
349. 陈金锋,雷忠义,刘超峰,郭利平.养心活血汤治疗冠心病不稳定型心绞痛的临床疗效观察[J].实用心脑肺血管病杂志,2018,26(06):140-143.
350. 雷舒扬. 养心颗粒治疗冠心病心绞痛气阴两虚证的临床观察[D]. 陕西中医药大学,2019.
351. 郭东浩. 养心汤加减治疗稳定型心绞痛（气虚血瘀证）的临床疗效及对RDW和PDW的影响[D].黑龙江中医药大学,2020.
352. 姜悦. 养心汤加味治疗气血虚滞型微血管性心绞痛临床研究[D].长春中医药大学,2018.
353. 赵永法,周亚滨,韩其茂,王浩飏.养心汤联合穴位贴敷治疗冠心病心绞痛[J].长春中医药大学学报,2019,35(02):264-266.
354. 安静. 养心通络汤治疗气虚血瘀型不稳定性心绞痛患者的随机对照研究[D].新疆医科大学,2020.
355. 徐美慧. 养心止痛汤加减联合胸痹贴治疗不稳定型心绞痛（心气不足证）的临床观察[D].黑龙江中医药大学,2020.
356. 郝月姣,司丹丹,邵静.养阴安神健脾方治疗气阴两虚型冠心病稳定性心绞痛疗效及对中医证候与心功能的影响[J].时珍国医国药,2021,32(07):1676-1679.
357. 陈玉洁. 养阴天香丹对冠心病稳定型心绞痛阴虚证的临床疗效观察[D].新疆医科大学,2018.
358. 苑春凤. 益气化痰活血方治疗老年稳定型心绞痛伴高Hcy血症的临床研究[D].云南中医学院,2017.
359. 王磊. 益气化痰祛瘀中药对冠心病稳定型心绞痛患者运动耐量的影响[D].辽宁中医药大学,2019.
360. 马磊. 益气化痰通脉方治疗PCI术后心绞痛（气虚痰瘀型）的临床研究[D].云南中医药大学,2020.
361. 赵成凯. 益气化瘀汤治疗冠心病不稳定型心绞痛（气虚血瘀）的临床疗效观察[D].山西中医药大学,2019.
362. 田止学,钱百成,王宝亮.益气化瘀汤治疗冠状动脉粥样硬化性心脏病心绞痛痰瘀互阻证临床研究[J].中医学报,2017,32(07):1251-1253.
363. 周美娟,董春彦,张铁军,刘宁宁.益气化浊利水方治疗卧位型心绞痛气虚血瘀证疗效研究[J].河北中医药学报,2022,37(02):22-25.
364. 郭玉. 益气豁痰化瘀汤治疗气虚痰瘀型老年稳定型心绞痛的临床研究[D].山东中医药大学,2020.
365. 张文. 益气豁痰汤治疗稳定性心绞痛气虚痰瘀证的临床疗效与运动耐量的研究[D]. 山东:山东中医药大学,2018.
366. 张悦. 益气活血、祛风止痛法治疗气虚血瘀型不稳定型心绞痛临床疗效观察[D]. 山东:山东中医药大学,2018.
367. 赵宗磊,杜松,罗萍,等. 益气活血法治疗不稳定型心绞痛PCI术后支架内再狭窄临床研究[J]. 中医学报,2017,32(11):2200-2203.
368. 陈红影. 益气活血法治疗冠心病稳定型心绞痛患者的临床疗效观察及对 MPV的影响[D]. 黑龙江:黑龙江中医药大学,2020.
369. 刘婧. 益气活血法治疗冠心病稳定型心绞痛的临床疗效观察[D].辽宁中医药大学,2021.
370. 丛敬,王柏山.益气活血方联合西药治疗冠心病心绞痛的临床观察[J].中华中医药学刊,2018,36(05):1048-1050.DOI:
371. 刘健,加拉力丁·买买提,董新玲,冷静,王晓峰.益气活血方治疗冠心病不稳定型心绞痛临床研究[J].陕西中医,2021,42(08):1037-1040.
372. 杨男. 益气活血复方联合西药治疗冠心病稳定型心绞痛的临床研究[D].延边大学,2018.
373. 白虹. 益气活血化瘀法干预PCI术后心绞痛患者（气虚血瘀证）临床观察[D].辽宁中医药大学,2020.
374. 王奇. 益气活血豁痰法对不稳定型心绞痛PCI术后的疗效观察[D].安徽中医药大学,2017.
375. 杨金龙,丁书文,姚晓东,王燕,于潇华,李晓.益气活血解毒方联合西药治疗经皮冠状动脉介入治疗术后心绞痛患者46例临床观察[J].中医杂志,2017,58(10):850-853.
376. 宋强,褚耀南,张海龙,张国恒,王小龙,孙宁波,苗小龙,杨春艳,杜鑫.益气活血清热解毒汤治疗冠心病血运重建术后心绞痛(卒心痛)的效果[J].中华中医药学刊,2019,37(07):1706-1709.
377. 万坤镇. 益气活血散治疗冠心病稳定型心绞痛（气虚血瘀型）的临床研究[D].成都中医药大学,2021.
378. 王国蕾,胡国恒,李亮,王瑾茜.益气活血汤治疗冠心病稳定型心绞痛的临床观察[J].现代中西医结合杂志,2017,26(26):2893-2895.
379. 王会哲. 益气活血汤治疗气虚血瘀型不稳定型心绞痛的临床研究[D].山东中医药大学,2018.
380. 李红英,陈少军,马友合,张宁,陈艳俏.益气活血汤治疗气虚血瘀型不稳定性心绞痛疗效观察[J].北京中医药,2017,36(07):633-636.
381. 柴宇燕. 益气活血通络膏方治疗经皮冠状动脉介入术后心绞痛气虚血瘀证临床疗效观察[D].新疆医科大学,2020.
382. 邓婷. 益气活血通脉方治疗气虚血瘀型老年冠心病SAP的临床观察[D].云南中医药大学,2020.
383. 马艳梅,李琳.益气通脉方联合美托洛尔治疗冠心病心绞痛临床疗效观察[J].中华中医药学刊,2021,39(09):173-176.
384. 杜国良,王泽荣,刁邵敏.益气通脉宁心饮辅助治疗急性冠脉综合征介入术后心绞痛的疗效观察[J].中西医结合心脑血管病杂志,2021,19(11):1857-1860.
385. 杨翠,郑思道,陈少军,陈艳俏.益气温阳活血方治疗不稳定心绞痛的临床研究[J].中西医结合心脑血管病杂志,2020,18(01):102-105.
386. 胡根胜. 益气养阴、化痰通络方干预冠心病（不稳定型心绞痛）合并2型糖尿病PCI患者临床疗效观察[D].安徽中医药大学,2018.
387. 倪子婷,邵正斌.益气养阴、化痰通络方治疗冠心病合并2型糖尿病PCI术后心绞痛临床研究[J].海南医学院学报,2020,26(06):439-443.
388. 李莲. 益气养阴定痛汤治疗冠心病稳定型心绞痛气阴两虚证的临床疗效观察[D].湖南中医药大学,2017.
389. 曹丽娟. 益气逐瘀通络汤治疗气虚血瘀型稳定性心绞痛的临床疗效观察及对运动耐量的影响[D].黑龙江中医药大学,2017.
390. 姜积军,陈静,徐永康,史凤磊.益肾活血汤对老年冠心病不稳定型心绞痛患者血管内皮功能及和肽素的影响[J].中国中医急症,2021,30(12):2189-2191.
391. 王宁,邢书成,任明芬.益心舒片联合地尔硫卓治疗稳定型劳力性心绞痛疗效观察[J].现代药物与临床,2019,34(03):652-656.
392. 郑雅琳. 益心通痹汤对冠心病稳定型心绞痛患者QT离散度影响的临床观察[D]. 四川:成都中医药大学,2017.
393. 凡永杰. 益心通脉膏方治疗冠心病稳定型心绞痛（痰瘀互结证）的临床观察[D]. 广西中医药大学,2019.
394. 郭平. 益心止痛方治疗稳定型心绞痛（气阴两虚，心脉瘀阻证）的临床观察[D].云南中医药大学,2021.
395. 周玉红,段卉娣,白云. 银丹心脑通软胶囊治疗老年冠心病心绞痛120例临床观察[J]. 中西医结合心脑血管病杂志,2017,15(4):457-460.
396. 郑爽. 银杏酮酯分散片对冠心病稳定型心绞痛（心血瘀阻证）心绞痛症状及中医证候的疗效观察[D].黑龙江中医药大学,2017.
397. 张静. 银杏酮酯分散片治疗冠心病稳定型心绞痛（心血瘀阻证）运动平板试验分析及中医证候的疗效观察[D].黑龙江中医药大学,2017.
398. 杨光. 玉竹牡蛎汤治疗心肾阴虚型稳定型心绞痛的临床研究[D].河北北方学院,2020.
399. 李倩. 育阴养心方治疗冠心病稳定型心绞痛（心肾阴虚证）的临床研究[D]. 陕西中医药大学,2020.
400. 张雪. 运用中医“性理疗病”心理干预法对冠心病不稳定性心绞痛患者生活质量的影响研究[D]. 吉林:长春中医药大学,2019.
401. 林牧荑. 增强型体外反搏联合荷丹胶囊对痰阻心脉型不稳定性心绞痛的临床观察[D].福建中医药大学,2020.
402. 陆忠,孙舟红.针刺结合活血方治疗不稳定型心绞痛临床疗效及对血管内皮功能的影响[J].中华中医药学刊,2018,36(05):1177-1180.
403. 巩倩惠,祁鹏,石立鹏,刘明怀,黄路梅,郭凯.针刺联合补阳还五汤治疗冠心病稳定型心绞痛的临床观察[J].中国中医急症,2018,27(11):1952-1955.
404. 石立鹏,杜旭勤,赵凤林,杨德钱,刘明怀,谭燕.针刺联合补阳还五汤治疗气虚血瘀证冠心病稳定型心绞痛疗效及对血脂水平的影响[J].现代中西医结合杂志,2017,26(35):3880-3882+3966.
405. 刘莉,张丹丹,邹国良,隋艳波,韩宇博,孙碧鸿.针刺联合温胆汤治疗痰瘀互结型稳定型心绞痛的疗效观察[J].上海针灸杂志,2021,40(04):379-384.
406. 严萍.针刺内关穴辅助温阳活血法联合前列地尔治疗不稳定性心绞痛临床观察[J].四川中医,2017,35(02):191-194.
407. 杨天虹. 针刺治疗脑梗死合并稳定型心绞痛的临床疗效观察[D].天津中医药大学,2020.
408. 孙艳,李博慧,王泽林.针刺治疗痰浊闭阻证稳定型心绞痛患者的效果[J].中国医药导报,2021,18(29):68-72.
409. 吴滨,刘亮,杨琳.针灸联合血府逐瘀汤对不稳定型心绞痛患者临床症状、血清过氧化氢酶水平及心率变异性影响[J].辽宁中医药大学学报,2020,22(03):180-184.
410. 潘立江.针灸联合盐酸曲美他嗪片治疗不稳定型心绞痛临床观察[J].上海针灸杂志,2019,38(10):1103-1108.
411. 岳胜南. 针药并用治疗痰瘀互结型冠心病不稳定性心绞痛的临床疗效观察[D].长春中医药大学,2020.
412. 王军媛,张军,刘颖,高阎满,孙佳佳,刘晓曼.针药结合治疗2型糖尿病合并冠心病心绞痛：随机对照研究[J].中国针灸,2021,41(04):371-375.
413. 魏星. 脂必泰对气虚痰瘀型稳定型心绞痛患者hs-CRP的影响[D].广州中医药大学,2017.
414. 王宇婷. 脂必泰胶囊对冠心病不稳定型心绞痛（痰瘀互结证）患者脂联素的影响[D]. 陕西中医药大学,2017.
415. 王伟. 枳壳煮散合正心泰方治疗稳定型心绞痛气滞血瘀证的临床研究[D]. 山东:山东中医药大学,2019.
416. 朱德建,陆曙. 枳实薤白桂枝汤对不稳定型心绞痛患者Hcy及hs-CRP影响[J]. 辽宁中医药大学学报,2018,20(6):209-212.
417. 朱德建,朱志仑,吴溧兴,陆曙.枳实薤白桂枝汤对不稳定性心绞痛患者血清基质金属蛋白酶-9水平及其基因表达的影响[J].河北中医,2021,43(12):2003-2007+2012.
418. 袁泉英,甘翔,鲍火庚,黎梓旺,刘希茜,李林.枳实薤白桂枝汤对冠心病不稳定型心绞痛痰浊痹阻型患者血清炎性因子及血管内皮功能的影响[J].中华中医药杂志,2021,36(11):6893-6897.
419. 方慧敏. 枳实薤白桂枝汤合半夏厚朴汤加减治疗稳定型心绞痛（痰阻心脉证）的临床观察[D]. 黑龙江:黑龙江中医药大学,2020.
420. 郑亿梁. 枳实薤白桂枝汤合丹参饮治疗痰气瘀结型冠心病不稳定型心绞痛的临床研究[D]. 山东:山东中医药大学,2019.
421. 闫红,余洪,潘小丹,范良,刘利涛.枳实薤白桂枝汤合理中汤加减辅助治疗稳定型心绞痛及对炎症因子和血管内皮功能的影响[J].中国实验方剂学杂志,2020,26(17):83-88.
422. 徐宁. 蛭曲化瘀汤治疗冠脉支架植入术后不稳定型心绞痛患者（气虚血瘀型）的临床疗效观察及对PAPP-A水平的影响[D].黑龙江中医药大学,2020.
423. 刘淑萍.中西医结合护理干预对老年不稳定性心绞痛临床疗效影响观察与分析[J].辽宁中医药大学学报,2017,19(07):213-215.
424. 刘洪. 中西医结合联合体外反搏治疗冠心病PCI术后心绞痛的临床研究[D].辽宁中医药大学,2018.
425. 田雨. 中西医结合联合增强型体外反搏治疗不稳定型心绞痛患者的临床研究[D].辽宁中医药大学,2018.
426. 董辉,王凤荣.中西医结合疗法对胸痹心痛寒凝血瘀证失眠患者的疗效观察[J].世界中西医结合杂志,2020,15(06):1076-1079.
427. 杨悦,滕涛,王健,张征.中西医结合治疗不稳定型心绞痛疗效观察[J].山东中医杂志,2017,36(06):478-480+484.
428. 刘云,董剑廷.中西医结合治疗急性心肌梗死心绞痛临床观察[J].西部中医药,2019,32(01):83-86.
429. 曹磊.中药对冠心病不稳定型心绞痛患者血管内皮的保护作用[J].世界中医药,2017,12(02):306-309.
430. 惠菊. 中药联合中医运动疗法干预稳定型心绞痛痰瘀互结证的临床研究[D].长春中医药大学,2020.
431. 南山,王学岭.中药汤剂辅助治疗气滞血瘀型心绞痛[J].吉林中医药,2017,37(08):784-787.
432. 于慧媛. 中药穴位贴治疗冠心病稳定型心绞痛（寒凝心脉证）的疗效观察[D].黑龙江中医药大学,2017.
433. 戴红红,姚福梅,纪焕春.中药药膳调理联合经络推按操缓解冠心病心绞痛的临床研究[J].河北医学,2019,25(02):269-272.
434. 黄传惠,樊瑞红,刘洪斌.中医辨证辅助治疗经皮冠状动脉介入术后心绞痛31例临床观察[J].中医杂志,2019,60(24):2118-2122.
435. 林琳. 中医五音疗法干预冠心病不稳定性心绞痛（气滞血瘀证）的临床研究[D].长春中医药大学,2020.
436. 孙彦琴.中医药联合体外反搏治疗难治性心绞痛临床研究[J].中医学报,2017,32(08):1495-1498.
437. 赵晓迪. 中医药治疗气虚痰瘀型冠心病稳定型心绞痛的临床研究[D].辽宁中医药大学,2018.
438. 常彩莲.中医综合疗法结合曲美他嗪治疗心绞痛疗效观察[J].西部中医药,2020,33(05):113-115.
439. 郭兴邦,胡美琴,高锐婷,李思.注射用益气复脉(冻干)联合尼可地尔治疗气阴两虚型微血管性心绞痛的临床评价[J].药物评价研究,2020,43(08):1597-1601.
440. 杭亮,田慧娟.自拟丹蒌合剂治疗冠心病心绞痛患者100例[J].环球中医药,2019,12(03):374-378.
441. 樊伟军.自拟宁心活血祛痰汤辅助西医规范方案治疗急性冠脉综合征PCI术后心绞痛疗效及对相关指标水平的影响[J].现代中西医结合杂志,2020,29(02):174-177.
442. 王海涛,刘艳军,黄春莉,赵鑫.自拟养心饮治疗冠状动脉粥样硬化性心脏病心绞痛患者70例[J].环球中医药,2018,11(12):2032-2035.
443. 许鸿燕,张在勇.自拟益气化瘀汤加味治疗冠心病稳定型心绞痛(气虚血瘀证)临床观察[J].中国中医急症,2017,26(08):1479-1481.
444. 张贺,张健,杜艳君,郑妍,胡晓美.子午流注纳子法穴位贴敷干预稳定性劳力型心绞痛的临床研究[J].中西医结合心脑血管病杂志,2021,19(09):1476-1479.
445. 范增光,周亚滨.足浴联合养心汤治疗冠心病稳定性心绞痛的临床观察[J].辽宁中医杂志,2017,44(09):1898-1900.
446. 雷程. 左归饮加减治疗冠心病稳定型心绞痛（心肾阴虚型）的疗效观察及对血清SOD和MDA含量的影响[D].黑龙江中医药大学,2019.
447. 骆霖. DQ方治疗冠心病稳定型心绞痛（痰瘀热毒互结证）的临床观察[D]. 陕西中医药大学,2017.
448. 罗建文. DQ胶囊对冠心病稳定型心绞痛临床疗效及Lp-PLA2的影响[D]. 陕西中医药大学,2019.
449. 吕林英,罗艺琼.自拟祛瘀通络方治疗气滞血瘀型心绞痛临床观察[J].辽宁中医杂志,2020,47(09):69-71.
450. 黄爱玲. “益气活血法”治疗冠脉慢血流心绞痛（气虚血瘀型）的临床研究[D].成都中医药大学,2019.
451. 顾迎春,李征艳,孙漾丽,等. 心脏康复运动联合中药气雾剂对冠心病患者心肺运动耐量及生活质量的影响[J]. 中国老年学杂志,2022,42(6):1284-1287.

# Supplementary file 5 List of excluded articles (only in the step of full-text screening) in this study

***Note****: In this section, 569 were excluded as not eligible for the journal requirements in this study; 32 were excluded as quasi-RCT and not RCT; 24 were excluded as not identified as ITCWM interventions; 4 were excluded as study protocol; 1 was excluded as duplicate records both published in Chinese and English; 1 was excluded as similar symptoms but not be diagnosed as angina pectoris.*

*For example, 1 study stated that "the general observation group was only treated with conventional angina treatment plan", it was difficult to judge whether the "conventional angina treatment plan" belonged to Western medicine treatment or not, although it described the intervention of TCM, so the article was excluded on the grounds of "non-ITCWM".*

1. 马中霞,赵森林.“硝酸甘油”加味“栝蒌薤白半夏汤”治疗不稳定型心绞痛患者的临床观察与疗效[J].中西医结合心血管病电子杂志,2019,7(05):156-157.
2. 杨琴,石磊,赵延雷. 42例稳定型心绞痛患者的中西药结合治疗分析[J]. 东方药膳,2021(4):113.
3. 崔靖娴. 阿替洛尔联合益心舒片治疗急诊冠心病心绞痛患者的临床疗效观察[J]. 现代诊断与治疗,2021,32(14):2207-2208.
4. 潘春兰. 阿托伐他汀联合复方丹参片治疗高血压不稳定型心绞痛的临床研究[J]. 医学食疗与健康,2021,19(25):102-103.
5. 石玉萍. 爱维心口服液联合美托洛尔治疗双心疾病疗效观察[J]. 世界最新医学信息文摘（连续型电子期刊）,2020,20(45):140-141,143.
6. 张倩,张焱.白玉参景脉通汤对冠心病心绞痛IL-1β水平的影响[J].光明中医,2021,36(04):551-553.
7. 张渊博,吉锋.百合舒心汤对冠心病PCI术后心绞痛患者血清CK、CK-MB、LDH水平的影响[J].现代医学与健康研究电子杂志,2020,4(09):76-77.
8. 曲畅,孙久林,陈艳俏.保元桃红四物汤和阿司匹林治疗冠心病心绞痛的效果观察[J].当代医药论丛,2019,17(02):199-200.
9. 袁智宇,周鹏飞,白倩,朱贺.保元养心方治疗冠心病稳定性心绞痛气阴两虚兼血瘀型35例[J].中医研究,2017,30(07):12-16.
10. 方鹏军,李洁. 倍他乐克联合通心络治疗冠心病心绞痛的临床疗效研究[J]. 饮食保健,2021(17):74.
11. 郜晓露. 辨证分型联合西药治疗心绞痛(气阴两虚/瘀血痹阻)随机平行对照研究[J]. 实用中医内科杂志,2019,33(1):33-35.
12. 肖红刚. 补气固脱中药联合阿托伐他汀治疗不稳定型心绞痛虚寒证的临床效果[J]. 内蒙古中医药,2021,40(10):26-28.
13. 杨帆. 补气通络方外敷对冠心病心绞痛患者病情及安全性分析[J]. 首都食品与医药,2021,28(11):131-132.
14. 杨钟堡. 补肾活血法治疗老年冠心病心绞痛疗效观察[J]. 心理医生,2018,24(18):5-6.
15. 李世鹏.补肾活血针法联合太极拳运动对冠心病稳定性心绞痛患者症状及生命质量的影响[J].中华心脏与心律电子杂志,2017,5(03):138-140.
16. 郝蕊. 补阳还五汤加减联合常规西医对冠心病心绞痛的疗效分析[J]. 家庭医药,2019(8):202-203.
17. 彭青. 补阳还五汤加减治疗冠心病心绞痛的临床观察[J]. 光明中医,2018,33(14):2062-2064.
18. 张红丽. 补阳还五汤加味治疗老年冠心病稳定型心绞痛气虚血瘀证的临床疗效[J]. 内蒙古中医药,2017,36(2):33.
19. 邓君进. 补阳还五汤联合针刺内关穴治疗冠心病不稳定型心绞痛(气虚血瘀型胸痹)的疗效研究[J]. 中国社区医师,2019,35(24):116.
20. 姜启峰. 补阳还五汤治疗老年冠心病稳定型心绞痛的临床疗效研究[J]. 中国实用医药,2020,15(15):141-142.
21. 郑伟. 不稳定型心绞痛(气滞血瘀证)患者运用中西医结合治疗治疗的临床疗效观察[J]. 母婴世界,2018(10):61.
22. 罗磊,杨娜,梁晗,等. 不稳定型心绞痛并室性心律失常中联合应用胺碘酮和中成药稳心颗粒的临床疗效观察[J]. 中国生化药物杂志,2017,37(11):229-230.
23. 欧济东. 不稳定型心绞痛采用加减瓜蒌薤白半夏汤治疗的效果探究[J]. 健康管理,2020(16):95.
24. 徐爱敏. 不稳定型心绞痛患者应用曲美他嗪联合复方丹参滴丸对射血分数和心电图变化的影响[J]. 数理医药学杂志,2021,34(3):419-421.
25. 林谦,张东耀,魏俊平,翟理黄,杨明,罗清菊,宋颖民,孙艳玲.参桂胶囊治疗冠心病稳定型心绞痛(心阳不振、气虚血瘀证)随机、双盲双模拟、阳性药平行对照、多中心临床研究[J].中医临床研究,2018,10(23):7-10.
26. 李江文.参桂胶囊治疗冠心病心绞痛58例临床观察[J].光明中医,2017,32(20):2904-2905.
27. 杜海波,齐锋,王义强,张兆志,李双娣,李雪.参红化浊通络颗粒治疗微血管性心绞痛的临床观察[J].中国卫生标准管理,2017,8(16):107-109.
28. 何玉清.参苓白术散联合瓜蒌薤白半夏汤对胸痹的治疗作用研究[J].心理月刊,2020,15(09):162.
29. 张国华. 参苓活血胶囊结合西医救治不稳定心绞痛的临床探讨[J]. 医学美学美容,2020,29(9):112.
30. 敖玉涵,张明雪,王秭萱. 参芪瓜蒌半夏薤白汤加减治疗经皮冠状动脉介入术后患者心绞痛再发临床疗效观察[J]. 中医药临床杂志,2018,30(5):912-916.
31. 郭普玉. 参芪益气活血方辅助常规西药治疗冠心病心绞痛的效果评价[J]. 临床研究,2018,26(8):122-124.
32. 张敏. 参芎葡萄糖注射液治疗冠心病心绞痛的临床疗效及药理学分析[J]. 中国医药指南,2020,18(9):14-15.
33. 袁艺,马方霞,詹云. 参松养心胶囊辅助治疗稳定型心绞痛心血瘀阻证的临床研究[J]. 按摩与康复医学,2021,12(15):35-38.
34. 刘红旭,李享,尚菊菊,等. 参元益气活血胶囊对气虚血瘀证不稳定性心绞痛患者PCI前后中医证候积分和血清TNF-α水平影响的临床研究[C]. //第十四届南方中医心血管病研讨会论文集. 2018:63-69.
35. 刘丽伟. 藏红花红景天与中药组方联合治疗冠心病心绞痛的疗效分析[J]. 内蒙古中医药,2017,36(2):24.
36. 杜晓雪,刘莉. 柴胡加龙骨牡蛎汤加减治疗冠心病心绞痛合并焦虑状态临床疗效观察[J]. 世界最新医学信息文摘（连续型电子期刊）,2018,18(A2):295-296,299.
37. 高原. 柴胡加龙骨牡蛎汤治疗稳定型心绞痛伴焦虑症状患者的临床效果分析[J]. 中国现代药物应用,2020,14(4):191-193.
38. 吴永刚,蒋守涛,徐玉莲. 柴胡疏肝散合血府逐瘀汤加减治疗气滞血瘀型稳定型心绞痛的临床疗效观察[J]. 医药前沿,2020,10(21):211-213.
39. 刘文书. 柴胡疏肝散加结治疗冠心病和病焦虑症[J]. 养生保健指南,2020(9):261.
40. 王丽,边振.柴胡疏肝散联合单硝酸异山梨酯片治疗冠心病心绞痛伴抑郁障碍40例[J].河南中医,2018,38(08):1178-1181.
41. 刘明顺.菖远护心汤联合西药治疗PCI术后心绞痛痰瘀阻络证患者的效果[J].实用临床医学,2020,21(11):5-7+10.
42. 王淑洁. 常规西药配合自拟中药益气活血汤治疗冠心病心绞痛88例分析[J]. 首都食品与医药,2017,24(12):84-85.
43. 马金浩. 川连枳桔汤治疗冠心病心绞痛临床观察[J]. 光明中医,2022,37(4):621-623.
44. 居秧琴,王道成,董娟,等. 醋调心衰方穴位贴敷联合通心痹合剂佐治胸痹心痛病临床效果观察[J]. 实用临床护理学电子杂志,2018,3(48):1-2,24.
45. 顾刚强.丹参川芎嗪注射液治疗冠心病不稳定型心绞痛临床研究[J].新中医,2019,51(06):114-116.
46. 刘岩.丹参川芎汤对冠心病伴心绞痛患者血清hs-CRP及IL-18水平的影响[J].中国中医药现代远程教育,2018,16(05):101-102.
47. 郭惠芳.丹参多酚酸盐(注射用)治疗老年冠心病心绞痛患者有效性和安全性分析[J].临床医药文献电子杂志,2018,5(93):36-37.
48. 辛文豪,冯文化.丹参多酚酸盐注射治疗冠心病心绞痛的疗效观察[J].中国药物滥用防治杂志,2021,27(05):767-770.
49. 常笑超.丹参联合辛伐他汀对冠心病患者心绞痛效果分析[J].中国城乡企业卫生,2018,33(04):129-130.
50. 苏静,童远义,肖玉洪.丹参酮ⅡA磺酸钠注射液联合曲美他嗪治疗冠心病的疗效及其对患者生活质量的影响[J].海南医学,2018,29(17):2383-2386.
51. 赵贺. 丹参饮合温胆汤治疗痰瘀阻滞型稳定型心绞痛的效果及对患者心肌缺血标志物水平的影响[J]. 当代医药论丛,2021,19(13):190-191.
52. 陈惠,钱春红. 丹红注射液治疗血瘀型心绞痛200例[J]. 河南中医,2017,37(7):1207-1209.
53. 杨广元. 当归四逆汤加减联合麝香保心丸治疗老年冠心病心绞痛疗效分析[J]. 家有孕宝,2020,2(15):192.
54. 林静欢,詹万慧,钟清珍. 地奥心血康胶囊和复方丹参滴丸对冠心病心绞痛患者的治疗效果及安全性分析[J]. 心血管病防治知识,2020,10(8):26-28.
55. 钟宇锋. 灯盏生脉胶囊辅助治疗高龄气虚血瘀型冠心病稳定性心绞痛的效果分析[J]. 云南中医中药杂志,2020,41(1):44-46.
56. 徐成军. 涤痰通脉汤联合氯吡格雷治疗冠心病心绞痛的疗效探讨[J]. 特别健康,2020(20):34-35.
57. 蔡媛珍,王蓉,蔡康霓,等. 颠倒木金散治疗气滞血瘀型冠心病稳定型心绞痛的临床疗效研究[J]. 健康之家,2021(9):70-71.
58. 聊俊婷. 调脂通脉汤联合拜阿司匹灵与阿托伐他汀钙治疗冠心病不稳定型心绞痛的疗效分析[J]. 当代医学,2020,26(10):136-137.
59. 李卫萍,袁震,安小莲,等. 丁酉年运气方对痰阻血瘀型胸痹心痛病的疗效评价[J]. 中西医结合心血管病电子杂志,2019,7(5):140-142.
60. 梅希玲,范小会.定心针法针刺联合定冠汤口服治疗冠心病心绞痛的临床疗效及对血清缺血修饰白蛋白、P选择素水平的影响[J].中医研究,2022,35(02):42-45.
61. 邓鹏,胡丹,刘中勇,等. 冬病夏治“强心贴”防治冠心病心绞痛的随机对照研究[J]. 中医药临床杂志,2017,29(4):498-501.
62. 支颢. 对接受介入治疗后出现心绞痛的冠心病患者用通阳泄浊活血颗粒剂进行治疗的效果[J]. 当代医药论丛,2018,16(23):181-183.
63. 梁爱江,吴乐文,罗斐莉,杨帆,郑凯特,苏峰祥.耳穴埋豆联合穴位贴敷治疗冠心病心绞痛的疗效观察[J].中医临床研究,2018,10(17):19-21.
64. 钱富生.分析瓜蒌薤白白酒汤联合心俞穴位注射丹参注射液治疗冠心病心绞痛的临床疗效[J].中国农村卫生,2020,12(07):39+41.
65. 白雪. 分析血府逐瘀汤加减治疗冠心病心绞痛的临床疗效[J]. 世界最新医学信息文摘（连续型电子期刊）,2018,18(29):149.
66. 王娇,闫曦,崔玉荣,等. 分析血液透析并发心绞痛患者应用护理干预联合复方丹参滴丸与硝酸甘油的疗效[J]. 东方药膳,2020(18):143.
67. 彭仲祥,张保国,温淑端. 附辛汤治疗稳定型心绞痛阳气亏虚证40例疗效观察[J]. 湖南中医杂志,2018,34(7):58-60.
68. 张立春,田成海. 复方川芎胶囊联合阿托伐他汀治疗冠心病心绞痛伴血脂异常的临床观察[J]. 临床医药文献电子杂志,2018,5(A4):202,211.
69. 张晓慧.复方川芎胶囊治疗冠心病心绞痛的疗效及对C-反应蛋白与脑钠肽的影响[J].中国民间疗法,2021,29(21):83-85.
70. 卫保林,曾引翠,程伟.复方丹参滴丸干预治疗慢性肺心病高凝状态的临床观察[J].山西职工医学院学报,2017,27(02):50-51.
71. 郑月敏. 复方丹参滴丸结合阿司匹林治疗稳定型心绞痛的疗效观察[J]. 内蒙古中医药,2020,39(5):57-58.
72. 杨得贵.复方丹参滴丸联合阿司匹林治疗30例冠心病心绞痛患者的临床观察[J].吉林医学,2017,38(9):1726-1727
73. 李卉.复方丹参滴丸联合阿司匹林治疗冠心病的临床观察[J].航空航天医学杂志,2018,29(07):873-874.
74. 杨文聪,尹建蓝,林祥煌,等. 复方丹参滴丸联合阿司匹林治疗老年不稳定型心绞痛的效果[J]. 中国保健营养,2018,28(23):19-20.
75. 蔡婷,兰艳丽. 复方丹参滴丸联合阿托伐他汀治疗不稳定性心绞痛疗效观察[J]. 延安大学学报（医学科学版）,2018,16(2):49-51.
76. 刘东振. 复方丹参滴丸联合氨氯地平治疗高血压性冠心病心绞痛疗效分析[J]. 医学信息,2017,30(13):100-101.
77. 韩鹰,李青. 复方丹参滴丸联合苯磺酸左旋氨氯地平治疗冠心病心绞痛的临床探讨[J]. 临床医药文献电子杂志,2019,6(21):160-161.
78. 李金娥.复方丹参滴丸联合单硝酸异山梨酯治疗冠心病不稳定型心绞痛临床研究[J].新中医,2021,53(20):45-49.
79. 张黄健. 复方丹参滴丸联合酒石酸美托洛尔缓释片治疗冠心病心绞痛的效果及对心电图变化和血液流变学的影响[J]. 交通医学,2020,34(5):472-474.
80. 郝志强. 复方丹参滴丸联合酒石酸美托洛尔对冠心病心绞痛患者症状改善及心功能的影响[J]. 临床医药文献电子杂志,2019,6(82):147,150.
81. 李曼,程晋芳,杜海萍,等. 复方丹参滴丸联合氯吡格雷治疗不稳定型心绞痛的效果及对血清一氧化氮与内皮素-1水平的影响[J]. 中国临床实用医学,2019,10(3):16-20.
82. 刘香. 复方丹参滴丸联合尼可地尔治疗冠心病心绞痛的疗效观察[J]. 内蒙古中医药,2021,40(5):55-56.
83. 余玛丽. 复方丹参滴丸联合曲美他嗪治疗冠心病心绞痛的临床效果观察[J]. 白求恩医学杂志,2017,15(3):347-349.
84. 张书军. 复方丹参滴丸联合曲美他嗪治疗不稳定心绞痛临床疗效观察[J]. 临床研究,2017,25(1):96-97.
85. 刘友萍,荣惠,沈相福. 复方丹参滴丸联合曲美他嗪治疗不稳定型心绞痛临床观察[J]. 中国中医药现代远程教育,2020,18(16):121-123.
86. 李晓斌,朱晓萍. 复方丹参滴丸联合曲美他嗪治疗不稳定型心绞痛临床效果[J]. 临床医学研究与实践,2017,2(28):11-12.
87. 张玉辉,宋颖. 复方丹参滴丸联合曲美他嗪治疗不稳定性心绞痛的临床疗效[J]. 内蒙古中医药,2020,39(6):78-79.
88. 赵家宁. 复方丹参滴丸联合曲美他嗪治疗不稳定性心绞痛的临床研究[J]. 养生保健指南,2018(16):238,190.
89. 邢铁艳,朱佳. 复方丹参滴丸联合通脉养心丸治疗慢性稳定性心绞痛临床疗效观察[J]. 亚太传统医药,2017,13(13):131-133.
90. 蔚华. 复方丹参滴丸配合替格瑞洛在冠心病PCI术后心绞痛患者中的应用[J]. 健康女性,2022(7):80-83.
91. 张艳华. 复方丹参滴丸与曲美他嗪联合应用治疗不稳定型心绞痛的疗效观察[J]. 中西医结合心血管病电子杂志,2018,6(36):155.
92. 江芸,严瑜,李海燕. 复方丹参滴丸与曲美他嗪联合治疗老年冠心病伴心绞痛的临床疗效分析[J]. 中国初级卫生保健,2017,31(6):72-73.
93. 崔玉荣. 复方丹参滴丸与硝酸甘油联合护理干预对血液透析并发心绞痛发作疗效分析[J]. 健康必读,2020(20):16,28.
94. 张居东,党志毅,温小玲. 复方丹参滴丸与辛伐他丁治疗不稳定型心绞痛临床疗效观察[J]. 饮食保健,2018,5(6):59.
95. 姜君. 复方丹参滴丸治疗冠心病心绞痛的疗效分析[J]. 中西医结合心血管病电子杂志,2016,4(21):42.
96. 彭鹏. 复方丹参滴丸治疗冠心病心绞痛的临床疗效观察[J]. 医学美学美容,2020,29(24):106.
97. 梁承树. 复方丹参滴丸治疗冠心病心绞痛的有效性和安全性研究[J]. 特别健康,2017(24):282.
98. 曾其辉. 复方丹参滴丸治疗冠心病心绞痛观察研究[J]. 健康忠告,2021,15(2):84,87.
99. 龙显强. 复方丹参滴丸治疗冠心病心绞痛临床研究[J]. 中西医结合心血管病电子杂志,2018,6(26):55.
100. 何丽芬,耿向东,姚红艳. 复方丹参滴丸治疗冠状动脉粥样硬化性心脏病心绞痛临床观察[J]. 中西医结合心血管病电子杂志,2018,6(11):122,124.
101. 李婧. 复方丹参滴丸治疗冠状动脉粥样硬化性心脏病心绞痛临床观察[J]. 健康之友,2021(12):264.
102. 王更磊,王清照. 复方丹参滴丸治疗急性心绞痛的临床观察[J]. 医学新知杂志,2018,28(z1):130-131.
103. 王小勇. 复方丹参片联合曲美他嗪对冠心病不稳定型心绞痛的疗效分析[J]. 中西医结合心血管病电子杂志,2018,6(30):57,60.
104. 张春伟. 复方丹参片联合曲美他嗪片治疗冠心病不稳定型心绞痛临床效果观察[J]. 健康必读,2020(12):84-85.
105. 张春伟. 复方丹参片联合曲美他嗪片治疗冠心病不稳定型心绞痛临床效果观察[J]. 特别健康,2019(24):28-29.
106. 张滢,林方芬,潘娅洁. 复方丹参注射液治疗冠心病稳定型心绞痛的临床研究[J]. 浙江中医杂志,2020,55(12):926-927.
107. 张秀丽. 复方丹参注射液联合西药治疗冠心病心绞痛患者临床疗效观察及对患者血脂水平的影响观察[J]. 贵州医药,2021,45(7):1127-1128.
108. 骆新莹. 复方三七护脉汤治疗冠心病心绞痛的临床疗效观察[J]. 现代诊断与治疗,2020,31(12):1874-1875,1972.
109. 孙鲁军. 复方血栓通胶囊辅治冠心病心绞痛临床观察[J]. 实用中医药杂志,2018,34(5):579-580.
110. 雷修华. 钙通道阻滞剂联合地奥心血康胶囊治疗冠心病心绞痛临床疗效的比较研究[J]. 临床合理用药杂志,2021,14(9):90-91.
111. 杨学奇,许滔. 甘露通脉超微粉水丸治疗冠心病心绞痛血瘀脉浊型临床疗效观察[J]. 世界最新医学信息文摘,2017(94).
112. 文国顺,王艳妮. 葛根素注射液治疗冠心病心绞痛的有效性及安全性[J]. 临床医学研究与实践,2017,2(27):114-115.
113. 张金兰,张晓艳. 隔姜灸疗法缓解稳定性心绞痛的临床研究[J]. 中国保健营养,2017,27(29):90-91.
114. 颜根姬. 隔物灸联合中医食疗干预在冠心病心绞痛患者中的应用[J]. 心血管病防治知识,2022,12(9):83-85.
115. 郑莹芊,彭廷云. 隔药饼灸干预阳虚水泛型冠心病心绞痛40例效果观察[J]. 湖南中医杂志,2021,37(4):98-100.
116. 郑莹芊,何继承,彭廷云. 隔药灸合针刺干预慢性稳定性心绞痛60例效果观察[J]. 湖南中医杂志,2021,37(8):102-104.
117. 王强. 瓜蒌川芎饮治疗冠心病经皮冠状动脉介入治疗术后痰阻血瘀型心绞痛的疗效分析[J]. 大医生,2020,5(23):84-86.
118. 叶彪,沈国君,额日登巴音. 瓜蒌薤白白酒汤对胸痹心痛的疗效观察[J]. 糖尿病天地,2018,15(7):35.
119. 张艳召. 瓜蒌薤白白酒汤治疗冠心病心绞痛临床观察[J]. 光明中医,2019,34(10):1540-1542.
120. 朱德建,王强,缪世荣,等. 瓜蒌薤白半夏汤对不稳定型心绞痛患者MMP-9及TIMP-1的影响[J]. 河南中医,2021,41(3):357-360.
121. 黄洪军. 瓜蒌薤白半夏汤改善痰浊闭阻型胸痹心痛患者胸闷、心悸等中医证候的临床研究[J]. 东方药膳,2020(8):205.
122. 周统菊. 瓜蒌薤白半夏汤合涤痰汤加减疗法对痰浊闭阻型胸痹心痛患者的疗效[J]. 家庭医药.就医选药,2020(12):146.
123. 信焕阳. 瓜蒌薤白半夏汤合温胆汤联合硝酸甘油治疗冠心病心绞痛的临床观察[J]. 中国民间疗法,2019,27(13):41-42.
124. 张明妍,白发臣,张燕. 瓜蒌薤白半夏汤合温胆汤治疗不稳定性心绞痛的疗效[J]. 深圳中西医结合杂志,2021,31(23):56-58.
125. 侯明亮. 瓜蒌薤白半夏汤合血府逐瘀汤治疗不稳定型心绞痛痰瘀互结证临床观察[J]. 中国中医药现代远程教育,2022,20(7):80-83.
126. 牛扬威. 瓜蒌薤白半夏汤加减辅治冠心病心绞痛痰浊痹阻型疗效观察[J]. 实用中医药杂志,2022,38(4):599-601.
127. 姜庆廷. 瓜蒌薤白半夏汤加减治疗冠心病不稳定型心绞痛临床疗效及应用价值[J]. 健康必读,2021(1):70-71.
128. 刘钰书. 瓜蒌薤白半夏汤加减治疗冠心病心绞痛患者的临床疗效研究[J]. 中国民康医学,2018,30(22):79-80.
129. 王礼. 瓜蒌薤白半夏汤加减治疗冠心病心绞痛痰浊痹阻证 对于心肌血运重建的影响[J]. 心理医生,2018,24(10):71-72.
130. 黄培培,赵燕峰,梁钰芩. 瓜蒌薤白半夏汤加减治疗冠状动脉粥样硬化性心脏病心绞痛40例[J]. 湖南中医杂志,2020,36(3):41-43.
131. 刘碧波. 瓜蒌薤白半夏汤加减治疗痰浊闭阻型胸痹的临床分析[J]. 系统医学,2022,7(8):39-42.
132. 高化强,田相同. 瓜蒌薤白半夏汤加味联合阿司匹林片治疗痰浊痹阻型稳定型心绞痛的临床疗效观察[J]. 健康必读,2020(32):23.
133. 柴松波. 瓜蒌薤白半夏汤结合血府逐瘀汤治疗冠心病心绞痛临床观察[J]. 光明中医,2018,33(24):3673-3675.
134. 仇卫锋,雒军强. 瓜蒌薤白半夏汤联合阿托伐他汀治疗冠心病心绞痛的疗效及对患者微炎性反应的影响[J]. 海南医学,2021,32(17):2197-2200.
135. 沈文学. 瓜蒌薤白半夏汤联合涤痰汤加减在痰浊闭阻型胸痹心痛中的影响[J]. 健康大视野,2018(13):101.
136. 曹培镇. 瓜蒌薤白半夏汤联合美托洛尔治疗痰浊内阻型冠心病心绞痛的临床观察[J]. 云南中医中药杂志,2021,42(9):55-57.
137. 郑小波. 瓜蒌薤白半夏汤联合西药治疗稳定型心绞痛痰浊闭阻证临床观察[J]. 中国中医药现代远程教育,2020,18(24):134-137.
138. 孙锦江. 瓜蒌薤白半夏汤联合西药治疗冠心病心绞痛75例[J]. 光明中医,2022,37(3):496-499.
139. 康玉顺. 瓜蒌薤白半夏汤联合硝酸甘油治疗痰浊壅塞型不稳定型心绞痛临床观察[J]. 中国中医药现代远程教育,2021,19(17):95-97.
140. 张元丽. 瓜蒌薤白半夏汤联合硝酸甘油治疗冠心病心绞痛临床疗效观察[J]. 东方药膳,2020(4):81-82.
141. 杨仲秋. 瓜蒌薤白半夏汤治疗对痰盛瘀阻型心绞痛患者症状改善及临床治疗总有效率的影响[J]. 实用中西医结合临床,2021,21(1):17-18.
142. 寇培军. 瓜蒌薤白半夏汤治疗冠心病心绞痛(痰浊痹阻)随机平行对照研究[J]. 实用中医内科杂志,2018,32(8):32-34.
143. 齐帅. 瓜蒌薤白丹参饮联合西药治疗心阳不振型冠心病心绞痛55例[J]. 中医研究,2021,34(5):25-28.
144. 杨晶. 观察常规西医基础上加用黄芪桂枝五物汤加味对阳虚脉阻证冠心病稳定型心绞痛的临床治疗价值[J]. 康颐,2020(8):210.
145. 胡宗艳. 观察冠心病心绞痛采用中医治疗的临床效果[J]. 养生保健指南,2019(23):243.
146. 丁岩. 观察黄芪桂枝五物汤加味应用于阳虚脉阻证冠心病稳定型心绞痛患者的临床治疗价值[J]. 东方药膳,2019(24):58.
147. 余绍清,陆虎,李路迢,等. 冠脉疏通汤治疗稳定型心绞痛型冠心病(气虚痰瘀痹阻证)60例临床观察[J]. 中国农村卫生,2020,12(20):31.
148. 吴轩. 冠舒汤合六味地黄治疗稳定型劳力性心绞痛41例临床观察[J]. 内蒙古中医药,2017,36(20):35-36.
149. 张俊涛. 冠心病不稳定型心绞痛应用中西医结合治疗的临床疗效研究[J]. 中西医结合心血管病电子杂志,2018,6(10):139-140.
150. 罗振阳. 冠心病常规治疗基础上加用曲美他嗪联合复方丹参滴丸治疗不稳定型心绞痛的疗效评价[J]. 全科口腔医学杂志（电子版）,2019,6(2):154.
151. 冯翠贤.冠心病心绞痛患者采用四逆汤加减联合氯吡格雷治疗的效果观察[J].中西医结合心血管病电子杂志,2017,5(09):103-104.
152. 张洁. 冠心病心绞痛患者使用中医护理干预的效果研究[J]. 光明中医,2018,33(21):3254-3256.
153. 张建民,龚志科. 冠心病心绞痛患者心血管内科治疗的临床效果分析与探讨[J]. 临床医学工程,2020,27(12):1609-1610.
154. 刘璇,孔明茹,王珺. 冠心病心绞痛患者行中医辨证护理的临床观察[J]. 光明中医,2018,33(13):1965-1967.
155. 冯燕妮. 冠心病心绞痛患者应用苦碟子注射液辅助治疗的临床效果探析[J]. 承德医学院学报,2022,39(2):129-132.
156. 张鑫,王静. 冠心病心绞痛临床诊断及治疗观察分析[J]. 饮食保健,2018,5(29):57-58.
157. 张志霞. 冠心病心绞痛心内科规范治疗的临床效果[J]. 国际全科医学,2022,3(2).
158. 王静. 冠心病心绞痛心内科规范治疗的临床效果[J]. 东方药膳,2020(7):118.
159. 张建. 冠心病心绞痛心内科规范治疗的临床效果[J]. 中西医结合心血管病电子杂志,2020,8(14):190-191.
160. 余丽云. 冠心病心绞痛心内科规范治疗的临床效果[J]. 家庭医药.就医选药,2020(12):54.
161. 李京. 冠心病心绞痛心血管内科的临床治疗[J]. 健康管理,2021(13):27.
162. 韦天煦. 冠心病心绞痛心血管内科治疗的临床分析[J]. 中西医结合心血管病电子杂志,2018,6(34):40.
163. 王梓豪,权菡. 冠心病心绞痛心血管内科治疗临床分析[J]. 健康前沿,2018,27(12):159.
164. 李德宏. 探讨中药联合西药治疗炎症性肠病的临床效果及不良反应[J]. 健康必读,2020(9):176.
165. 刘世福. 冠心病心绞痛中医辨证治疗分析[J]. 心理医生,2018,24(25):125-126.
166. 王海兵,张天豪,张学斌. 冠心静胶囊治疗冠心病心绞痛的临床疗效及经济学评价[J]. 中国药物评价,2017,34(1):62-66.
167. 戴玉兰,白慧荣. 冠心静胶囊治疗气虚血瘀型心绞痛的临床研究[J]. 当代医学,2021,27(22):28-30.
168. 孙慧敏,鲁文涛,张军鹏,等. 冠状动脉再通膏对不稳定型心绞痛患者斑块稳定性、细胞凋亡因子及凝血指标变化的影响[J]. 河南中医,2018,38(6):867-870.
169. 李洁,曹胜兰,缪翠. 红花黄色素结合个体化护理干预在不稳定型心绞痛患者中的临床研究[J]. 现代医学与健康研究（电子版）,2021,5(14):130-133.
170. 凌果元,鲍世涛. 红花黄色素联合硝酸甘油治疗冠状动脉粥样硬化性心脏病心绞痛患者的临床效果[J]. 中国药物经济学,2022,17(2):57-60.
171. 闫曦,韩朋丽,崔玉荣,等. 护理干预联合复方丹参滴丸与硝酸甘油对血液透析并发心绞痛发作的效果[J]. 医学美学美容,2021,30(2):175.
172. 曹辉,石立鹏,杜旭勤,等. 化痰祛瘀汤治疗冠心病心绞痛30例疗效观察[J]. 湖南中医杂志,2017,33(11):5-7.
173. 段菊花. 化痰祛瘀通脉汤联合琥珀酸美托洛尔缓释片对不稳定型心绞痛的治疗效果[J]. 河南医学研究,2021,30(2):321-323.
174. 张春芝. 化痰通络治疗胸痹心痛病的临床效果评价[J]. 大家健康（中旬版）,2018,12(5):44-45.
175. 费凯,单晓晶,单金平. 化痰逐瘀方联合体外反搏治疗对经皮冠状动脉介入治疗术后心绞痛患者疼痛、血管内皮因子及不良情绪的影响[J]. 中国药物经济学,2022,17(4):42-46,58.
176. 蒋德军,燕学波,余忠志. 化瘀通冠汤联合曲美他嗪治疗冠心病稳定型心绞痛的效果[J]. 临床医学,2020,40(7):115-117.
177. 赵海东. 化瘀通脉汤剂剂治疗冠心病心绞痛的预后效果观察[J]. 中西医结合心血管病电子杂志,2019,7(29):157,159.
178. 刘美兰,王清华. 化瘀通脉汤剂联合西药治疗冠心病心绞痛55例临床疗效分析[J]. 中国保健营养,2021,31(11):98.
179. 李妍. 化瘀通脉汤剂治疗冠心病心绞痛的疗效分析[J]. 中国医药指南,2017,15(16):203-204.
180. 马桂云. 化瘀通脉汤剂治疗冠心病心绞痛的效果观察[J]. 养生保健指南,2018(7):307.
181. 陈雍慧. 化瘀通脉汤剂治疗冠心病心绞痛分析[J]. 中国卫生标准管理,2020,11(1):109-111.
182. 郭江水. 化瘀通脉汤联合西药治疗冠心病心绞痛临床观察[J]. 光明中医,2021,36(7):1142-1144.
183. 童倩,黄建强. 皇龙益气胶囊治疗气虚血瘀型冠心病心绞痛的双盲试验研究[J]. 中国现代药物应用,2020,14(24):229-231.
184. 杨丽. 黄连温胆汤加减治疗冠心病心绞痛的临床效果[J]. 河南医学研究,2019,28(20):3755-3757.
185. 齐帅,贺小武,王晓飞. 黄连温胆汤联合西药治疗不稳定型心绞痛53例临床观察[J]. 中国民族民间医药,2018,27(17):117-119.
186. 吴怡,马晓旭. 黄连温胆汤治疗冠心病稳定型心绞痛的疗效分析[J]. 现代医学与健康研究（电子版）,2021,5(22):74-76.
187. 何跃东. 黄芪桂枝五物汤加味联合常规西医疗法治疗冠心病稳定型心绞痛的疗效观察[J]. 航空航天医学杂志,2020,31(7):829-830.
188. 廖郁文,沈智理. 黄芪养阴通脉汤治疗气阴两虚型稳定性心绞痛临床观察[J]. 山西中医,2020,36(12):20-21.
189. 张正,邹仁妹. 黄芪益参汤加减治疗冠心病心绞痛(气虚血瘀)随机平行对照研究[J]. 实用中医内科杂志,2019,33(2):10-12.
190. 赵艺,崔会营,孙亚茹. 豁痰化瘀汤对痰浊阻痹证冠心病心绞痛患者症状改善及血脂水平的影响[J]. 广西中医药,2021,44(6):20-22.
191. 衡狮静. 豁痰化瘀汤联合硝酸异山梨酯片治疗不稳定性心绞痛的疗效分析[J]. 医师在线,2022,12(5):31-32.
192. 吴小强. 活络消痛汤联合美托洛尔对不稳定性心绞痛的治疗效果[J]. 河南医学研究,2020,29(20):3789-3791.
193. 杨大勇. 活血涤痰法治疗冠心病心绞痛的临床观察[J]. 光明中医,2018,33(4):530-531,534.
194. 李国诗,黄淼鑫,潘枚霞,等. 活血解毒中药对PCI患者围手术期心肌损伤的影响研究[J]. 中国医药科学,2020,10(5):11-14,31.
195. 刘小平,陈苏宁. 活血通脉安神汤治疗不稳定心绞痛伴失眠(气滞血瘀)随机平行对照研究[J]. 实用中医内科杂志,2018,32(10):18-20,71.
196. 何兆辉,王志谦,王国良,等. 活血逐瘀汤联合尼可地尔治疗微血管性心绞痛临床研究[J]. 新中医,2020,52(18):60-63.
197. 惠菊,孙燕,王兴,等. 基于心脏康复单元的中药联合中医运动疗法干预冠心病稳定型心绞痛痰瘀互结证的临床研究[J]. 世界最新医学信息文摘（连续型电子期刊）,2020,20(27):152-153.
198. 韩豪,牟宗毅.基于瘀能化水理论的稳心Ⅳ号治疗冠心病心绞痛痰瘀互结型的临床探讨[J].世界复合医学,2019,5(12):19-21.
199. 曹军,李峰,鲁锦国,孙跃玲,肖赢.加服加味小陷胸汤治疗PCI术后难治性心绞痛临床观察[J].广西中医药大学学报,2018,21(03):14-17.
200. 陈任跃,谢海波.加减柴陷汤治疗冠心病心绞痛伴失眠30例临床观察[J].湖南中医杂志,2019,35(05):4-7.
201. 王小霞. 加减血府逐瘀汤联合曲美他嗪治疗冠心病不稳定型心绞痛临床疗效分析[J]. 心理月刊,2020,15(10):205.
202. 邓海文. 加减血府逐瘀汤联合曲美他嗪治疗冠心病不稳定型 心绞痛临床效果分析[J]. 中外医疗,2019,38(13):163-165.
203. 刘绍峰. 加减血府逐瘀汤联合曲美他嗪治疗冠心病不稳定型心绞痛临床分析[J]. 临床医药文献电子杂志,2018,5(18):150-151.
204. 李素梅,鲁国志. 加减血府逐瘀汤联合曲美他嗪治疗冠心病不稳定型心绞痛临床研究[J]. 临床医药文献电子杂志,2018,5(66):159-160.
205. 杨为亚,单建芳. 加减血府逐瘀汤联合曲美他嗪治疗冠心病不稳定型心绞痛价值体会[J]. 心血管外科杂志（电子版）,2018,7(2):214-215.
206. 何川. 加减血府逐瘀汤联合曲美他嗪治疗冠心病不稳定型心绞痛临床疗效分析[J]. 东方药膳,2020(5):214.
207. 连拴巧,王伟红. 加减血府逐瘀汤联合曲美他嗪治疗冠心病不稳定型心绞痛临床效果观察[J]. 母婴世界,2020(8):115.
208. 李桂莹. 加减血府逐瘀汤联合曲美他嗪治疗冠心病不稳定型心绞痛临床效果及对患者生活质量的影响[J]. 健康大视野,2018(13):89,87.
209. 王楠,徐文峰,赵军香. 加减血府逐瘀汤联合曲美他嗪治疗冠心病不稳定型心绞痛临床效果评价[J]. 心理医生,2018,24(16):181-182.
210. 丁萌. 加减血府逐瘀汤联合曲美他嗪 治疗冠心病不稳定型心绞痛效果评价[J]. 中西医结合心血管病电子杂志,2018,6(23):182-183.
211. 魏忠光. 加减血府逐瘀汤与曲美他嗪对冠心病心绞痛的应用效果及价值体会[J]. 心血管病防治知识,2021,11(25):26-28.
212. 万玲,石闯,孙彦波. 加味丹参饮联合西药治疗气滞血瘀型稳定型心绞痛的临床疗效[J]. 实用中医内科杂志,2020,34(11):78-81.
213. 王鑫,王宇航,褚成文. 加味瓜蒌薤白半夏汤对冠心病心绞痛患者血脂水平及血液流变学的影响[J]. 黑龙江医学,2019,43(10):1219-1220,1222.
214. 高培峰. 加味瓜蒌薤白半夏汤治疗冠心病心绞痛痰浊痹阻证的临床疗效分析[J]. 当代医药论丛,2020,18(15):205-207.
215. 姚斌,刘瑞,胡秀娟,等. 加味栝楼薤白汤治疗痰瘀互结型慢性稳定型心绞痛30例[J]. 湖南中医杂志,2019,35(9):5-7,21.
216. 王建安,刘中勇. 加味逍遥散治疗胸痹心痛临床观察[J]. 饮食保健,2018,5(2):89.
217. 马金凤. 加味血府逐瘀汤联合针刺治疗冠心病心绞痛的临床疗效观察[J]. 中国医药指南,2019,17(27):225.
218. 刘永光,杨柳,丁小妹,等. 加味真武汤联合热敏灸治疗稳定型心绞痛心肾阳虚证25例临床观察[J]. 湖南中医杂志,2017,33(10):44-45.
219. 蓝登科. 健脾益心方联合西药治疗冠心病稳定型心绞痛(心脾两虚)随机平行对照研究[J]. 实用中医内科杂志,2018,32(10):21-24.
220. 解冰,赵凤凤,陈亮. 解郁安神颗粒联合帕罗西汀治疗老年冠心病稳定型心绞痛伴抑郁的临床研究[J]. 临床荟萃,2021,36(2):121-124.
221. 王云龙,黄健. 开心散治疗冠心病心绞痛痰浊瘀阻证46例效果分析[J]. 中西医结合心血管病电子杂志,2017,5(1):86.
222. 张士海. 开胸通痹汤治疗痰阻血瘀型冠心病不稳定性心绞痛的临床疗效[J]. 世界最新医学信息文摘（连续型电子期刊）,2019,19(3):179-180.
223. 韩婉青,王永生,张宪芬,等. 苦碟子注射液联合曲美他嗪治疗冠心病心绞痛的疗效观察[J]. 现代药物与临床,2021,36(9):1830-1833.
224. 徐汉国. 老年高血压合并心绞痛患者接受综合治疗的疗效分析[J]. 大家健康（中旬版）,2017,11(9):106-107.
225. 李晶,张小飞. 老年冠心病心绞痛中西医结合治疗探究[J]. 科学养生,2021 (3):167.
226. 陈金锋,雷忠义,刘超峰,等. 雷氏养心活血汤治疗冠心病气阴两虚痰瘀互结证临床研究[J]. 陕西中医,2018,39(12):1691-1693.
227. 宣惠清,陈丽艳,苏飞. 离子导入加味丹参饮联合常规医护措施治疗稳定型心绞痛临床研究[J]. 新中医,2021,53(3):187-190.
228. 樊瑞红,吴宗贵,毕育学,等. 理气活血滴丸治疗冠心病慢性稳定性心绞痛的临床研究[J]. 世界最新医学信息文摘（连续型电子期刊）,2020,20(58):241-245,249.
229. 施志超,李付远,王华富,等. 联合应用活血化瘀中成药与阿司匹林治疗慢性稳定性心绞痛的临床效果及可行性探讨[J]. 海峡药学,2020,32(7):80-83.
230. 江秀琴,陈淑芹. 苓桂术甘汤加味治疗冠心病心绞痛的临床研究[J]. 心血管病防治知识,2019,9(30):31-33.
231. 王利红. 马酸比索洛尔片联合瑞舒伐他汀钙片治疗老年冠心病心绞痛患者的疗效观察[J]. 药品评价,2020,17(7):36-37.
232. 郝森. 脉血康胶囊联合尼可地尔对不稳定型心绞痛患者血管活性物质及细胞因子的影响[J]. 哈尔滨医药,2022,42(1):122-123.
233. 周锦良. 脉血康胶囊治疗冠心病不稳定型心绞痛的疗效[J]. 深圳中西医结合杂志,2019,29(12):59-60.
234. 张丽丽,李雁. 脉血康胶囊治疗冠心病心绞痛心血瘀阻型的临床研究[J]. 中国中医药现代远程教育,2018,16(8):96-98.
235. 刘一,齐文娟. 美托洛尔联合通心络治疗冠心病心绞痛的临床疗效[J]. 大医生,2021,6(8):5-7.
236. 段宏宇.尼可地尔联合复方丹参滴丸治疗微血管性心绞痛疗效比较[J].中西医结合心血管病电子杂志,2017,5(17):15+18.
237. 张霞. 评价复方丹参滴丸治疗冠心病心绞痛的有效性和安全性[J]. 饮食保健,2018,5(43):62-63.
238. 杜学航. 芪参胶囊治疗稳定型劳累性心绞痛240例临床疗效观察探究[J]. 江苏科技信息,2021,38(20):64-70.
239. 孙显中. 芪参益气滴丸联合比索洛尔对冠心病心绞痛的疗效[J]. 深圳中西医结合杂志,2020,30(5):41-43.
240. 王姣. 芪参益气滴丸联合单硝酸异山梨酯治疗心绞痛临床观察[J]. 光明中医,2020,35(2):267-269.
241. 蓝秋文,陈少文. 前列地尔联合红花黄色素治疗冠心病心绞痛患者的疗效[J]. 深圳中西医结合杂志,2019,29(23):39-41.
242. 陈睿. 曲美他嗪联合复方丹参滴丸治疗冠心病伴心绞痛患者的临床效果[J]. 中国社区医师,2018,34(18):86-87.
243. 周玉能. 曲美他嗪联合复方丹参滴丸治疗不稳定心绞痛疗效探讨[J]. 特别健康,2021(20):46-47.
244. 褚怡雯. 曲美他嗪联合炙甘草汤治疗不稳定性心绞痛临床疗效观察[J]. 现代养生（下半月版）,2018(6):136-137.
245. 杨晓艳,冯辉. 祛风稳斑汤治疗不稳定型心绞痛36例临床观察[J]. 湖南中医杂志,2017,33(7):3-5.
246. 薛艳丽,任琳琳. 祛瘀化痰汤联合常规西药治疗痰瘀互结型冠心病心绞痛50例[J]. 中医研究,2021,34(6):36-39.
247. 杨东. 瑞舒伐他汀联合单硝酸异山梨酯治疗心绞痛的临床有效性研究[J]. 北方药学,2018,15(8):17-18.
248. 崔琳琳. 三参救心汤治疗冠心病心绞痛临床观察[J]. 中国中医药现代远程教育,2022,20(8):81-82.
249. 周宏健,姜钧文. 散寒祛瘀方联合西药治疗寒凝血瘀证稳定型心绞痛临床观察[J]. 云南中医中药杂志,2021,42(8):50-53.
250. 景丽英,郑俊华. 麝香保心丸联合倍他乐克对冠心病心绞痛患者心电图及心功能指标的影响[J]. 中国医药科学,2021,11(14):71-74.
251. 郭萌薇. 麝香保心丸联合琥珀酸美托洛尔缓释片治疗冠心病稳定型心绞痛的临床研究[J]. 实用中西医结合临床,2022,22(4):14-16,20.
252. 孙彧. 麝香保心丸联合尼可地尔对不稳定型心绞痛患者经皮冠状动脉介入术术后的临床研究[J]. 实用医技杂志,2020,27(12):1664-1666.
253. 苏江波,马雅丽. 麝香保心丸联合瑞舒伐他汀治疗冠心病心绞痛的疗效分析[J]. 现代医学与健康研究（电子版）,2021,5(13):38-41.
254. 王晶晶,张红霞. 麝香保心丸联合硝酸异山梨酯与阿司匹林对冠心病心绞痛的效果[J]. 河南医学研究,2021,30(10):1867-1869.
255. 曹金凤. 麝香保心丸治疗冠心病心绞痛的临床观察[J]. 光明中医,2017,32(23):3420-3422.
256. 陈永忠,陈慧,林秀明,等. 麝香通心滴丸干预老年慢性稳定性心绞痛氯吡格雷抵抗40例[J]. 福建中医药,2017,48(5):57-59.
257. 安桐志. 升陷汤治疗冠心病心绞痛临床观察[J]. 中国中医药现代远程教育,2020,18(5):324-326.
258. 牟园园,马文欣,冯晓敬. 生柴汤治疗稳定型心绞痛(肝郁气滞证)32例临床观察[J]. 湖南中医杂志,2018,34(12):43-45.
259. 张文魁. 生脉散合丹参饮加减联合常规疗法治疗气阴两虚型稳定型心绞痛临床研究[J]. 新中医,2019,51(7):103-105.
260. 冉龙华. 生脉散汤剂联合阿托伐他汀治疗冠心病心绞痛的临床效果[J]. 中外医学研究,2020,18(5):149-151.
261. 徐宝年. 生脉散汤剂联合阿托伐他汀治疗冠心病心绞痛的效果[J]. 健康女性,2021(46):127.
262. 张向红,王正平. 生脉散汤剂联合阿托伐他汀治疗冠心病心绞痛的效果[J]. 特别健康,2021(30):58.
263. 杨秋雪. 生脉散汤剂联合阿托伐他汀治疗冠心病心绞痛的效果观察[J]. 养生大世界,2021(11):85.
264. 苏荣华. 生脉散汤剂联合阿托伐他汀治疗冠心病心绞痛的有效性分析[J]. 北方药学,2019,16(12):148-149.
265. 闫永波. 试析中西医疗法在冠心病心绞痛临床治疗疗效[J]. 母婴世界,2020(20):71.
266. 冯枫,柳晨. 疏肝解郁止痛汤治疗冠心病心绞痛的临床疗效研究[J]. 实用中西医结合临床,2020,20(13):60-62.
267. 辛龙光. 舒心散加减治疗老年冠心病心绞痛的82例效果观察[J]. 中国继续医学教育,2017,9(20):177-178.
268. 徐青怡. 舒心饮治疗稳定性心绞痛的疗效观察及护理[J]. 光明中医,2017,32(15):2268-2270.
269. 乔可明. 四逆汤加减联合氯吡格雷治疗冠心病心绞痛的疗效观察[J]. 大家健康（下旬版）,2017,11(5):20-21.
270. 赵受伟,余佳,潘英侃,等. 苏合香穴位贴敷治疗不稳定性心绞痛疗效观察[J]. 深圳中西医结合杂志,2020,30(2):55-56.
271. 丁慧,李嵩岩,仇年芳. 速效救心丸与盐酸地尔硫卓片联合用于不稳定型心绞痛患者的临床疗效及对心功能指标的影响[J]. 当代医学,2022,28(13):55-58.
272. 周玉凤. 痰浊内阻型胸痹心痛病患者的中西医辩证治疗[J]. 临床心身疾病杂志,2018,24(1):106-108.
273. 金燕. 探究生脉散汤剂联合阿托伐他汀治疗冠心病心绞痛的效果[J]. 东方药膳,2020(13):32.
274. 段春艳. 探究生脉散汤剂联合阿托伐他汀治疗冠心病心绞痛的效果[J]. 健康之友,2020(20):145.
275. 崔继红,于翠. 探讨低分子肝素钙与复方丹参联用治疗不稳定型心绞痛的效果[J]. 科学咨询,2020(43):38.
276. 尤玉玲. 探讨生脉散汤剂联合阿托伐他汀治疗冠心病心绞痛的有效性[J]. 健康之友,2020(15):9.
277. 石贺. 探讨中西医结合治疗冠心病冠脉支架植入术后再发心绞痛临床效果[J]. 中国保健营养,2020,30(3):331.
278. 牛光明. 探讨中西医结合治疗冠心病心绞痛临床疗效观察分析[J]. 健康女性,2021(51):44.
279. 刘磊,王雪莲,张静芳. 探讨中西医结合治疗冠心病心绞痛临床疗效观察分析[J]. 健康大视野,2022(9):144-145.
280. 黄丽娟.探析复方丹参滴丸与氯吡格雷片单用或联合应用对心绞痛患者血小板功能的影响研究[J].中国社区医师,2018,34(28):94-95.
281. 付贵. 探析血府逐瘀汤治疗冠心病心绞痛的临床效果及安全可靠性[J]. 健康忠告,2022,16(7):157-160.
282. 郝丽丽,路瑞华,唐文欣. 桃红四物汤加味联合常规西药治疗心血瘀阻型稳定型心绞痛的临床疗效及对血清超敏C反应蛋白、白细胞介素-6水平的影响[J]. 中国民间疗法,2022,30(11):64-67.
283. 张玉杰. 通脉化浊汤辅助西药对冠心病心绞痛患者血管内皮功能和血脂的影响[J]. 河南医学研究,2021,30(4):735-737.
284. 王楠,王凤荣,王帅,等. 通脉降浊汤治疗冠状动脉粥样硬化性心脏病稳定型心绞痛痰浊血瘀证临床观察[J]. 河南中医,2018,38(2):233-237.
285. 侯喆,刘瑞霞. 通脉养心丸治疗气阴两虚兼血瘀型冠心病心绞痛60例临床效果观察[J]. 世界最新医学信息文摘（连续型电子期刊）,2019,19(26):132,134.
286. 金雪玉,金永旭. 通脉止痛贴治疗不稳定型心绞痛(气虚血瘀型)的 临床疗效观察[J]. 中外医疗,2017,36(7):168-169,172.
287. 张丽春. 通心络胶囊对不稳定型心绞痛患者心电图及血清MMP-9、P选择素水平的影响[J]. 大医生,2020,5(13):83-85.
288. 左可可. 通心络胶囊对冠心病心绞痛(气虚血瘀型)患者的临床疗效观察[J]. 饮食保健,2020(40):92.
289. 苑立博. 通心络胶囊联合阿托伐他汀治疗冠心病心绞痛的临床疗效分析[J]. 临床研究,2018,26(4):58-60.
290. 潘启民. 通心络胶囊联合美托洛尔治疗冠心病心绞痛的疗效探讨[J]. 中国中医药现代远程教育,2018,16(24):120-121.
291. 李国境,程天太. 通心络胶囊治疗冠心病不稳定型心绞痛临床疗效观察[C]. //第十三届国际络病学大会论文集. 2017:243-245.
292. 蔡少杭,吴瑞华. 通心络胶囊治疗心肌梗死后心绞痛45例临床疗效观察[C]. //2018年医学前沿论坛暨第十四届国际络病学大会论文集. 2018:310-312.
293. 袁婧玮,高占群,李霜,等. 通心络联合阿司匹林治疗不稳定型心绞痛疗效分析[J]. 继续医学教育,2019,33(1):148-150.
294. 张敬,张连涛,董文娟. 通心贴穴位外治气滞血瘀型冠心病心绞痛临床研究[J]. 世界最新医学信息文摘（连续型电子期刊）,2019,19(73):172,174.
295. 苏慧,郑浩龙. 通阳宽胸颗粒辅助治疗气滞痰瘀证稳定型心绞痛临床效果分析[J]. 白求恩医学杂志,2020,18(5):455-457.
296. 马继华. 为痰阻血瘀型稳定型心绞痛患者采用中西医结合疗法进行治疗的效果观察[J]. 当代医药论丛,2019,17(18):178-179.
297. 叶剑,唐勇. 温脾通络开窍汤联合尼可地尔治疗微血管病变性心绞痛及对血管内皮功能与炎性因子水平的影响[J]. 延安大学学报（医学科学版）,2021,19(2):54-57.
298. 贾壮壮,王志刚. 温肾法治疗心肾阳虚型冠心病心绞痛43例疗效观察[J]. 湖南中医杂志,2017,33(9):56-58.
299. 尚晓萌,李永生,周朝伟. 温阳活血法治疗不稳定型心绞痛的疗效及对血清IL-6、TNF-α、hs-CRP的影响[J]. 哈尔滨医药,2020,40(6):582-583.
300. 刘超. 温阳宽胸活血法治疗冠心病心绞痛60例临床观察[J]. 中国城乡企业卫生,2017,32(7):105-106.
301. 张志军,蔡少杭,陈晖. 温阳益气活血汤联合西药治疗冠心病心绞痛43例[J]. 中医研究,2021,34(8):13-16.
302. 李婷,钟超伶. 温针灸与血府逐瘀汤联合常规疗法治疗冠心病心绞痛临床研究[J]. 新中医,2020,52(6):124-128.
303. 孙慧琳. 温针灸与血府逐瘀汤联合常规疗法治疗冠心病心绞痛临床研究[J]. 医学食疗与健康,2021,19(5):38-39.
304. 沈冬菊. 稳心颗粒联合胺碘酮治疗不稳定型心绞痛并室性心律失常临床观察[J]. 光明中医,2021,36(15):2600-2602.
305. 张忠华. 稳心颗粒联合苯磺酸氨氯地平对冠心病心绞痛患者血液流变学指标的影响[J]. 基层医学论坛,2021,25(34):4991-4993.
306. 孙立平,唐可清,李飞,等. 稳心汤治疗痰瘀互结型冠心病永久性房颤的临床观察[J]. 世界最新医学信息文摘（连续型电子期刊）,2018,18(72):213-214.
307. 唐晓雯,马桂妍. 我院冠心病联合用药使用分析[J]. 特别健康,2018(15):90-91.
308. 赵云. 吴茱萸穴位贴敷联合双足中药熏洗对胸痹心痛病气虚血瘀证患者疗效观察[J]. 中西医结合心血管病电子杂志,2020,8(6):16-18.
309. 张华涛,陈婕,李杰,等. 夏橘化瘀胶囊联合西医常规治疗气虚血瘀型冠心病稳定型心绞痛的疗效观察[J]. 河北中医,2017,39(9):1348-1351.
310. 郭兆友. 陷胸逐瘀汤治疗痰瘀毒互结型冠心病心绞痛的效果及对心肌耗氧量的影响分析[J]. 中国实用医药,2020,15(20):165-167.
311. 曹秀荣. 硝酸甘油片和复方丹参滴丸缓解急性心绞痛的临床疗效比较[J]. 中国实用医药,2018,13(35):1-3.
312. 林绍城,杨健兰,叶盛清. 硝酸异山梨酯、酒石酸美托洛尔联合益气化瘀方治疗不稳定型心绞痛气虚血瘀证的疗效观察[J]. 心血管病防治知识,2021,11(30):26-29.
313. 王慧禹,焦晓民. 小陷胸汤合温胆汤治疗冠心病心绞痛60例观察[J]. 中医药临床杂志,2017,29(2):219-221.
314. 侯宝松,李星红,刘霞,等. 小陷胸汤治疗痰热瘀阻型冠心病心绞痛临床研究[J]. 河南中医,2018,38(2):201-205.
315. 黄轲. 心达康胶囊联合尼可地尔治疗不稳定型心绞痛的效果[J]. 中国医学创新,2022,19(17):22-26.
316. 王秀峰. 心绞痛患者联用曲美他嗪及复方丹参滴丸的临床研究[J]. 黑龙江医药,2020,33(6):1288-1290.
317. 高涛. 心绞痛中西医结合治疗疗效分析[J]. 健康必读,2018(15):104,103.
318. 王明明,赵会珍. 心可舒片联合曲美他嗪治疗冠心病心绞痛的疗效[J]. 内蒙古中医药,2017,36(16):57.
319. 石丰富. 心脑欣丸对冠心病心绞痛患者心功能及血流动力学的影响[J]. 河南医学研究,2020,29(2):321-323.
320. 谢帆慈,郭志华. 心痛泰颗粒治疗不稳定型心绞痛30例临床观察[J]. 湖南中医杂志,2017,33(5):50-52.
321. 王瑞华. 心元胶囊联合尼可地尔治疗冠心病不稳定型心绞痛患者的效果[J]. 中国民康医学,2021,33(18):88-90.
322. 金英玲. 辛伐他汀片联合硫酸氢氯吡格雷片治疗不稳定型心绞痛的临床疗效[J]. 大医生,2019,4(14):118-119.
323. 杨雪,易学凤,刘露,等. 胸痹患者的中西医结合护理干预体会[J]. 中国医药科学,2018,8(18):131-133,253.
324. 姚鹏飞. 胸痹协定方联合西药治疗不稳定型心绞痛患者的临床观察[J]. 中国民间疗法,2021,29(19):82-85.
325. 曾成霞,谭大洲,朱元宵,等. 胸痹心痛病中医护理方案效果观察[J]. 世界最新医学信息文摘,2021,21(76):357-358.
326. 王芳,金昌洙. 胸痹心痛方配合耳穴压豆治疗气滞血瘀型稳定型心绞痛的临床疗效研究[J]. 反射疗法与康复医学,2021,2(3):45-48.
327. 马玮莉. 宣痹祛痰方治疗冠心病合并肥胖症痰瘀互结证疗效评价[J]. 陕西中医,2018,39(2):226-228.
328. 陈斌,姚斌. 萱草忘忧汤加减治疗冠心病心绞痛并焦虑24例临床观察[J]. 湖南中医杂志,2018,34(9):52-53.
329. 王珍,张志国,张志军. 玄红胸痛贴穴位贴敷联合西药治疗冠心病心绞痛45例[J]. 中医研究,2018,31(6):27-28.
330. 张娴,尹晓薇,谭英斌. 穴位敷贴治疗心肌桥患者心绞痛的疗效观察[J]. 心血管病防治知识,2018(18):36-37.
331. 孙碧鸿,隋艳波,张丹丹,等. 穴位贴敷联合柴胡疏肝散治疗冠心病稳定型心绞痛的临床观察[J]. 世界最新医学信息文摘（连续型电子期刊）,2019,19(16):140-141.
332. 袁玲玲,张丹. 穴位贴敷联合常规西药治疗冠心病稳定性心绞痛临床研究[J]. 新中医,2019,51(6):294-297.
333. 陆姝姝. 穴位贴敷联合耳穴贴压对冠心病心绞痛患者的影响[J]. 河南中医,2018,38(6):871-873.
334. 幸珍珍. 穴位贴敷联合分级运动图像训练对冠心病心绞痛患者预后生存质量的影响[J]. 黑龙江医药,2021,34(6):1309-1311.
335. 葛凤艳. 穴位贴敷治疗便秘合并胸痹心痛的临床护理价值分析[J]. 中国保健营养,2018,28(34):294-295.
336. 高雪娇. 穴位注射辅助治疗对冠心病患者心绞痛疗效的分析[J]. 中国疗养医学,2021,30(9):947-949.
337. 汤继海. 血府逐瘀汤对不稳定性心绞痛的疗效[J]. 深圳中西医结合杂志,2019,29(2):58-59.
338. 赵小麟,黄锦翘,刘兵阳. 血府逐瘀汤辅助治疗老年冠心病心绞痛对患者血浆同型半胱氨酸和血脂水平的影响[J]. 现代医学与健康研究（电子版）,2022,6(1):93-95.
339. 吴长玉. 血府逐瘀汤合六君子汤治疗冠心病心绞痛临床观察[J]. 中国中医药现代远程教育,2018,16(8):94-96.
340. 刘璐瑶. 血府逐瘀汤加减对血瘀阻滞型稳定型冠心病心绞痛的临床意义以及药理研究[J]. 光明中医,2021,36(1):62-64.
341. 李艳阳,李沫帆. 血府逐瘀汤加减辅助曲美他嗪治疗冠心病心绞痛的临床效果及对患者心绞痛症状、心功能及血清学指标的影响[J]. 临床医学研究与实践,2022,7(5):123-126.
342. 穆连财. 血府逐瘀汤加减联合曲美他嗪治疗冠心病不稳定型心绞痛的疗效分析[J]. 青海医药杂志,2020,50(11):40-42.
343. 银梓杉. 血府逐瘀汤加减联合曲美他嗪治疗冠心病不稳定型心绞痛的临床观察[J]. 中国医药指南,2021,19(3):123-124.
344. 王奎. 血府逐瘀汤加减联合曲美他嗪治疗冠心病不稳定型心绞痛[J]. 深圳中西医结合杂志,2017,27(22):26-27.
345. 魏秀敏,贾建峰,孙会芳,等. 血府逐瘀汤加减联合曲美他嗪治疗冠心病不稳定型心绞痛的疗效分析[J]. 中国保健营养,2020,30(25):350-351.
346. 黄文华,徐英妹,蒋凌飞,等. 血府逐瘀汤加减联合西药治疗冠心病心绞痛36例临床观察[J]. 湖南中医杂志,2018,34(9):7-9.
347. 代洪绪,翟文姬,项聿华. 血府逐瘀汤加减联合西医常规疗法治疗气滞血瘀型稳定型心绞痛的临床观察[J]. 中国民间疗法,2020,28(24):85-88.
348. 潘英华. 血府逐瘀汤加减用于冠心病心绞痛治疗的效果及可行性分析[J]. 养生大世界,2021(12):90-91.
349. 徐伟珍. 血府逐瘀汤加减治疗冠心病不稳定型心绞痛的方法及其预后价值[J]. 当代医学,2020,26(33):167-168.
350. 李鑫莹. 血府逐瘀汤加减治疗冠心病不稳定型心绞痛的效果[J]. 家庭医药·就医选药,2021(2):21-22.
351. 郝若妤. 乳腺钼靶X线检查在乳腺浸润性小叶癌与浸润性导管癌鉴别诊断中的影像学特征[J]. 中国民康医学,2020,32(16):104-106.
352. 姜志坤. 血府逐瘀汤加减治疗冠心病介入术后心绞痛临床研究[J]. 健康必读,2021(14):31.
353. 赵红松. 血府逐瘀汤加减治疗冠心病稳定型心绞痛对患者心功能及血液流变学的影响[J]. 现代医学与健康研究（电子版）,2020,4(9):72-73.
354. 刘永胜. 血府逐瘀汤加减治疗冠心病心绞痛的效果[J]. 医学信息,2020,33(z1):143-144.
355. 汤涵宇. 血府逐瘀汤加减治疗心血瘀阻型冠心病心绞痛患者及对心绞痛发作次数影响分析[J]. 健康之家,2021(14):34-35.
356. 梁子盟. 血府逐瘀汤加减治疗胸痹心痛的临床效果观察[J]. 中国医学创新,2022,19(8):76-79.
357. 孟宪磊,安炎霞,刘俊飞. 血府逐瘀汤联合阿司匹林对冠心病心绞痛的疗效[J]. 深圳中西医结合杂志,2020,30(14):25-26.
358. 程建生. 血府逐瘀汤联合阿司匹林对冠心病心绞痛患者的疗效观察[J]. 河南医学研究,2018,27(16):2968-2969.
359. 张巧宏. 血府逐瘀汤联合瓜蒌薤白半夏汤治疗冠心病心绞痛的效果探究[J]. 自我保健,2022,26(13):275-277.
360. 徐宝石. 血府逐瘀汤联合硫酸氢氯吡格雷治疗冠心病心绞痛的临床研究[J]. 家庭医药,2020(1):194.
361. 王飞. 血府逐瘀汤联合美托洛尔缓释片治疗老年人冠心病心绞痛的疗效评价[J]. 饮食保健,2019,6(43):59-60.
362. 郭新合. 血府逐瘀汤联合西药治疗冠心病心绞痛心血瘀阻证患者的临床观察[J]. 光明中医,2018,33(5):710-712.
363. 王自贵. 血府逐瘀汤联合西药治疗冠心病心绞痛(瘀血阻滞)随机平行对照研究[J]. 实用中医内科杂志,2018,32(12):18-20.
364. 周易,姜钧文. 血府逐瘀汤联合针灸治疗冠心病不稳定性心绞痛(心血瘀阻型)临床观察[J]. 中西医结合心血管病电子杂志,2019,7(16):147-148.
365. 樊亚崑,陶斯琪. 血府逐瘀汤配合穴位贴敷治疗不稳定型心绞痛43例[J]. 江西中医药大学学报,2018,30(6):63-65.
366. 吕红伟. 血府逐瘀汤与丹七软胶囊联合西药治疗心绞痛(瘀血内阻)随机平行对照研究[J]. 实用中医内科杂志,2019,33(4):49-52.
367. 杨金星. 血府逐瘀汤治疗冠心病心绞痛的疗效及患者用药不良反应分析[J]. 特别健康,2020(31):79.
368. 李淑静. 血府逐瘀汤治疗心脉瘀阻型冠心病心绞痛的疗效及对心功能、炎症因子的影响[J]. 黑龙江中医药,2021,50(5):120-121.
369. 秘相征,曲静,李峰. 血行气汤联合曲美他嗪与美托洛尔对冠心病心绞痛疗效及血小板功能指标的相关研究[J]. 家庭医药.就医选药,2020(4):207.
370. 王厚晋,杨威英. 延玉合剂治疗老年稳定劳力型心绞痛40例临床观察[J]. 湖南中医杂志,2017,33(9):9-11.
371. 宫照敏,张海瑞. 研究和分析冠心病合并心绞痛采用中医辨证治疗的临床效果[J]. 医药前沿,2018,8(3):353.
372. 连文涛. 研究生脉散汤剂联合阿托伐他汀治疗冠心病心绞痛的效果[J]. 健康之友,2021(16):46-47.
373. 尹玉花,刘会敏. 研究探讨冠心病心绞痛心内科规范治疗的临床体会[J]. 糖尿病天地,2021,18(9):187.
374. 张帆,丁碧云. 养心活血汤治疗冠心病PCI术后心绞痛疗效及对血脂的影响[J]. 中医药临床杂志,2018,30(9):1705-1708.
375. 周易,姜钧文. 养心散瘀方治疗冠心病PCI术后心绞痛临床观察[J]. 云南中医中药杂志,2020,41(3):39-42.
376. 柳小春,刘丽仙,林能明. 养心汤联合常规西药治疗心气虚弱型冠心病不稳定性心绞痛临床研究[J]. 新中医,2019,51(3):80-83.
377. 万亚琼,钟文强. 养心通络方辅助治疗胸痹气虚血瘀证(心绞痛)疗效观察[J]. 中国保健营养,2018,28(31):118.
378. 杨兴隆,安桐志. 养阴益气汤对冠状动脉粥样硬化性心脏病心绞痛患者血管内皮因子及血脂的影响[J]. 中国药物经济学,2022,17(4):59-63.
379. 孙郁松. 益气补血化瘀汤治疗冠心病不稳定心绞痛的临床效果及对临床指标的影响[J]. 临床合理用药杂志,2021,14(11):6-8.
380. 贾建军. 益气化痰消脂汤对稳定型心绞痛伴高脂血症患者血脂血小板聚集率及脂蛋白相关磷脂酶A2的影响[J]. 中国中医药科技,2017,24(5):616-617,621.
381. 万科,田锋平,李文进,等. 益气化瘀豁痰方治疗冠心病心绞痛临床研究[J]. 新中医,2019,51(7):113-116.
382. 徐富武,吴燕华. 益气活血补肾汤联合阿司匹林肠溶胶囊对冠心病不稳定型心绞痛患者NT-proBNP、MPO、hs-CRP的影响[J]. 中国医学创新,2022,19(16):66-70.
383. 李成,姜贵民,刘亚娟,等. 益气活血法在冠心病心绞痛治疗中的应用[J]. 饮食保健,2021(31):123-124.
384. 李郁春,陶永. 益气活血方对冠心病(稳定型心绞痛)PCI术后的影响[J]. 中医药临床杂志,2017,29(4):506-509.
385. 孟莉. 益气活血方治疗冠心病介入术后心绞痛的临床效果[J]. 内蒙古中医药,2022,41(2):61-63.
386. 周淑平. 益气活血化痰配方颗粒治疗微血管性心绞痛的疗效观察[J]. 中国医学创新,2018,15(26):65-69.
387. 张进. 益气活血化痰通络方治疗冠心病稳定型心绞痛的疗效观察[J]. 首都食品与医药,2021,28(19):159-161.
388. 杨超. 益气活血化浊汤联合富马酸比索洛尔对冠心病心绞痛患者症状及生活质量的影响[J]. 河南医学研究,2019,28(7):1284-1286.
389. 董峰,吕艳斐,丁书文,等. 益气活血解毒法治疗不稳定型心绞痛52例疗效分析[J]. 中国中医药现代远程教育,2018,16(18):44-45.
390. 梁文赵. 益气活血类中药配伍治疗冠心病心绞痛的疗效[J]. 内蒙古中医药,2017,36(18):59-60.
391. 马天阳,曹家铭,李成,等. 益气活血汤联合西药治疗冠心病稳定型心绞痛(气虚血瘀证)的临床观察[J]. 中国民间疗法,2021,29(13):87-89.
392. 苏文秀. 益气活血汤联合西药治疗气虚血瘀型冠心病心绞痛的临床观察[J]. 光明中医,2018,33(22):3386-3388.
393. 张永敏. 益气活血汤治疗冠心病稳定型心绞痛疗效及对炎症因子的影响[J]. 饮食保健,2020,7(5):120.
394. 曹元龙. 益气活血中药方辅助治疗不稳定性心绞痛的临床疗效观察[J]. 中西医结合心血管病电子杂志,2018,6(12):136,138.
395. 付玉娜,李武卫. 益气升降汤治疗冠心病心绞痛宗气亏虚证的临床研究[J]. 西北国防医学杂志,2019,40(9):557-561.
396. 李晓娜,丁立辉. 益气通痹膏治疗气虚血瘀型冠心病心绞痛临床观察[J]. 光明中医,2019,34(8):1187-1190.
397. 王贵勇. 益气通脉汤结合西药在冠心病心绞痛中的治疗观察[J]. 科学养生,2022 (8): 63-64.
398. 李俊亮. 益气通脉汤治疗冠心病合并心绞痛的临床疗效[J]. 内蒙古中医药,2021,40(3):76-77.
399. 芮浩淼. 益气通阳化瘀汤联合西药治疗冠心病心绞痛76例[J]. 中医研究,2018,31(3):21-23.
400. 张志国,范立华,孙静,等. 益气泄浊祛瘀方联合西药对冠心病心绞痛气虚浊瘀互结证 血清ET、NO的影响[J]. 中医研究,2018,31(8):13-15.
401. 傅祖伟,杨海燕. 益气宣痹方联合西药治疗不稳定型心绞痛的疗效分析[J]. 现代实用医学,2017,29(4):446-448.
402. 翟颖,沙树伟,刘淑荣. 益气养阴化痰活血法治疗糖尿病合并冠心病心绞痛的临床研究[J]. 临床医药文献电子杂志,2019,6(93):60,79.
403. 李明子,蔡群,宋宇新,等. 益气养阴化瘀汤治疗气阴两虚兼瘀型胸痹30例临床观察[J]. 湖南中医杂志,2020,36(12):5-7.
404. 陈丽. 益气养阴活血化瘀法治疗冠心病心绞痛临床观察[J]. 健康大视野,2018(18):94-95.
405. 张敏,吴柳容. 益气滋阴活血方治疗冠心病稳定型 心绞痛（气阴两虚兼瘀证）的临床观察[J]. 大健康,2022(9):113-115.
406. 曹杰,王卫星. 益心康泰胶囊治疗冠心病心绞痛(气虚血瘀证)合并血脂异常临床研究[J]. 亚太传统医药,2018,14(1):195-196.
407. 孟宪亮. 益心通络胶囊治疗不稳定型心绞痛患者PCI术后再发42例临床观察[J]. 湖南中医杂志,2020,36(6):1-5.
408. 董治华. 银丹心脑通软胶囊治疗糖尿病合并冠心病心绞痛临床观察[J]. 养生保健指南,2018(27):95.
409. 黄永翔,曾伟雄.银丹心泰滴丸联合倍他乐克治疗对冠心病心绞痛患者IL-18及hs-CRP水平的影响分析[J].心血管病防治知识,2021,11(26):25-27.
410. 丁弘莘,张竞锋. 银杏蜜环口服溶液治疗高血压合并冠心病心绞痛的临床疗效研究[J]. 黑龙江医学,2022,46(11):1349-1351,1354.
411. 冯木生. 银杏酮酯滴丸辅助治疗冠心病心绞痛疗效观察[J]. 深圳中西医结合杂志,2018,28(18):25-27.
412. 黄民. 银杏叶胶囊联合阿司匹林在稳定性心绞痛治疗中的应用价值[J]. 自我保健,2021(5):217.
413. 杨丽君. 用宽心安神配方颗粒治疗冠心病心绞痛合并失眠的疗效观察[J]. 当代医药论丛,2017,15(24):173-175.
414. 莫婷. 用生脉散汤剂治疗糖尿病并发冠心病心绞痛的效果分析[J]. 糖尿病天地,2019,16(2):37.
415. 邹晓明. 越鞠丸加味从"郁"论治冠心病心绞痛临床研究[J]. 光明中医,2021,36(3):375-379.
416. 杨琴. 针刺联合硝酸异山梨酯片与琥珀酸美托洛尔缓释片治疗冠心病心绞痛患者的临床价值分析[J]. 科学养生,2020 (7):203-204.
417. 宋柏奇,齐锋. 针刺治疗气虚血瘀型微血管性心绞痛的临床观察[J]. 临床医药文献电子杂志,2017,4(31):5958-5959.
418. 张金来. 针灸配合药物治疗胸痹心痛临床观察[J]. 光明中医,2022,37(11):1976-1979.
419. 余婧. 针灸治疗对冠心病心绞痛患者血流变及LDL-C、HDL-C水平的影响[J]. 现代医学与健康研究（电子版）,2020,4(1):137-138.
420. 梁吉,雷志芹,田文雯,等. 针灸治疗冠心病的疗效观察[J]. 养生保健指南,2021(3):104.
421. 邓林林,范军铭,毕巧莲. 针药结合治疗不稳定型心绞痛的临床疗效及对髓过氧化物酶、超敏C反应蛋白、血脂的影响[J]. 中医研究,2021,34(10):21-23.
422. 毕金阳. 针药结合治疗冠心病心绞痛的价值分析与研究[J]. 中国保健营养,2020,30(17):35.
423. 王丁仓,路彩霞. 枳实薤白桂枝汤合小陷胸汤治疗痰热内阻型稳定型心绞痛临床研究[J]. 新中医,2022,54(12):56-59.
424. 彭思思. 中西结合治疗冠心病心绞痛临床研究[J]. 饮食保健,2017,4(13):76.
425. 张永胜. 中西结合治疗心绞痛的优势及效果[J]. 医学美学美容,2020,29(10):90.
426. 王艳红. 中西药合用治疗不稳定型心绞痛临床观察[J]. 实用中医药杂志,2019,35(2):172-174.
427. 王银娜,黄培培,郝秀梅,等. 中西药合用治疗不稳定性心绞痛疗效分析[J]. 实用中医药杂志,2017,33(6):642-643.
428. 杨精华. 中西药合用治疗冠心病不稳定型心绞痛疗效观察[J]. 实用中医药杂志,2020,36(3):311-312.
429. 张月如. 中西药合用治疗冠心病临床分析[J]. 实用中医药杂志,2018,34(5):589-590.
430. 谈晓东. 中西药合用治疗冠心病心绞痛疗效观察[J]. 实用中医药杂志,2019,35(1):47-48.
431. 王冰珂,都万卿. 中西药合用治疗冠心病心绞痛气虚瘀阻型效果观察[J]. 实用中医药杂志,2018,34(10):1225-1226.
432. 姜捷. 中西药合用治疗冠心病心绞痛痰瘀闭阻证疗效观察[J]. 实用中医药杂志,2022,38(6):976-978.
433. 任琼. 中西药联合治疗冠心病心绞痛临床疗效和安全性[J]. 家庭医药.就医选药,2020(4):134.
434. 刘颖,张军,王军媛,等. 中西药治疗2型糖尿病合并稳定性心绞痛的临床研究[J]. 临床医药文献电子杂志,2017,4(50):9703-9704,9706.
435. 张琪. 中西医辨证治疗痰浊内阻型胸痹心痛病患者的临床疗效[J]. 中西医结合心血管病电子杂志,2020,8(24):176,179.
436. 吴怡. 中西医护理对冠心病心绞痛的效果观察[J]. 内蒙古中医药,2017,36(21):241-242.
437. 李建云. 中西医结合对冠心病心绞痛的治疗体会[J]. 北方药学,2017,14(1):185.
438. 刘洪,宫丽鸿. 中西医结合联合体外反搏治疗冠心病PCI术后患者的疗效及对血清VEGF的影响[J]. 云南中医中药杂志,2018,39(1):20-22.
439. 冯臻谛,冯胜春,梁静华,等. 中西医结合穴位埋线治疗不稳定型心绞痛临床研究[J]. 新中医,2022,54(14):170-175.
440. 包正波. 中西医结合在冠心病心绞痛中的疗效分析[J]. 饮食保健,2020,7(27):77.
441. 周元涓. 中西医结合在治疗冠心病心绞痛上的临床应用效果观察[J]. 饮食保健,2020,7(23):91.
442. 于佳卓. 中西医结合治疗52例冠心病心绞痛的临床效果观察[J]. 健康大视野,2020(8):114-115.
443. 徐乐. 中西医结合治疗不稳定型心绞痛36例临床观察[J]. 中国保健营养,2020,30(19):380-381.
444. 敬满芳. 中西医结合治疗不稳定型心绞痛48例疗效观察[J]. 大家健康（中旬版）,2017,11(9):69.
445. 吴启宏. 中西医结合治疗冠心病不稳定型心绞痛的临床疗效分析[J]. 饮食保健,2018,5(33):86-87.
446. 鲁克难. 中西医结合治疗冠心病不稳定型心绞痛的临床研究[J]. 养生大世界,2021(11):97.
447. 田荣静. 中西医结合治疗冠心病不稳定型心绞痛的效果[J]. 大医生,2019,4(13):93-94.
448. 邱代伟. 中西医结合治疗冠心病不稳定型心绞痛的效果研究[J]. 当代医药论丛,2018,16(12):46-47.
449. 牛根良. 中西医结合治疗冠心病不稳定型心绞痛疗效观察[J]. 实用中医药杂志,2017,33(6):639-640.
450. 蒋长胜. 中西医结合治疗冠心病不稳定型心绞痛临床观察[J]. 实用中医药杂志,2017,33(6):640-642.
451. 林建设. 中西医结合治疗冠心病不稳定型心绞痛临床观察[J]. 实用中医药杂志,2018,34(5):559-560.
452. 牛红,李岩. 中西医结合治疗冠心病不稳定型心绞痛临床观察[J]. 中西医结合心血管病电子杂志,2018,6(10):143-144.
453. 王跃蕊. 中西医结合治疗冠心病不稳定型心绞痛临床观察[J]. 实用中医药杂志,2018,34(8):972-973.
454. 李伟. 中西医结合治疗冠心病不稳定型心绞痛痰浊痹阻型疗效观察[J]. 实用中医药杂志,2018,34(5):587-588.
455. 关小甫. 中西医结合治疗冠心病疗效分析[J]. 深圳中西医结合杂志,2020,30(9):43-44.
456. 周敬法. 中西医结合治疗冠心病微血管心绞痛临床观察[J]. 光明中医,2017,32(7):1032-1034. DOI:10.3969/j.issn.1003-8914.2017.07.053.
457. 吴芳,宋榜林. 中西医结合治疗冠心病稳定型心绞痛临床疗效观察[J]. 血栓与止血学,2021,27(4):566-568.
458. 宋建辉,刘熙浩,乔亚光,等. 中西医结合治疗冠心病稳定型心绞痛气虚痰瘀证临床研究[J]. 医药前沿,2017,7(22):115-116.
459. 张占锋. 中西医结合治疗冠心病稳定型心绞痛气虚血瘀证临床观察[J]. 云南中医中药杂志,2018,39(2):29-30.
460. 张天鹏. 中西医结合治疗冠心病稳定性心绞痛的疗效观察[J]. 健康大视野,2021(13):97.
461. 高琦. 中西医结合治疗冠心病稳定性心绞痛的疗效评价研究[J]. 母婴世界,2021(26):113-114.
462. 王碧柏. 中西医结合治疗冠心病稳定性心绞痛痰阻血瘀证的效果评价[J]. 医药前沿,2020,10(5):127.
463. 董二飞. 中西医结合治疗冠心病稳定性心绞痛痰阻血瘀证的效果评价[J]. 现代养生（下半月版）,2017(5):176.
464. 李成芳. 中西医结合治疗冠心病心绞痛合并焦虑抑郁症40例临床观察[J]. 中国民族民间医药,2018,27(12):118-120.
465. 张保珠. 中西医结合治疗冠心病心绞痛43例临床观察[J]. 中国民族民间医药,2018,27(24):122-124.
466. 鲁燕. 中西医结合治疗冠心病心绞痛51例临床观察[J]. 中国民族民间医药,2018,27(18):92-94.
467. 朱明. 中西医结合治疗冠心病心绞痛60例临床疗效观察[J]. 中外女性健康研究,2017(6):25,36.
468. 欧霞. 中西医结合治疗冠心病心绞痛65例临床疗效分析[J]. 名医,2018(6):69.
469. 刘晓霞,刘怀斌,薛彦艳,等. 中西医结合治疗冠心病心绞痛伴发抑郁症的疗效分析[J]. 饮食保健,2020,7(7):99-100.
470. 袁志达. 中西医结合治疗冠心病心绞痛的疗效观察[J]. 内蒙古中医药,2020,39(11):50-51.
471. 吴春静. 中西医结合治疗冠心病心绞痛的疗效观察[J]. 中西医结合心血管病电子杂志,2017,5(30):181,183.
472. 任剑军. 中西医结合治疗冠心病心绞痛的疗效及应用优势探析[J]. 世界最新医学信息文摘（连续型电子期刊）,2018,18(A1):182.
473. 宗述正. 中西医结合治疗冠心病心绞痛的临床分析[J]. 中西医结合心血管病电子杂志,2018,6(3):162.
474. 张海昀. 中西医结合治疗冠心病心绞痛的临床观察[J]. 光明中医,2018,33(12):1781-1783.
475. 刘湛一. 中西医结合治疗冠心病心绞痛的临床疗效观察[J]. 世界最新医学信息文摘,2021,21(54):233-234.
476. 陈波. 中西医结合治疗冠心病心绞痛的临床疗效观察[J]. 糖尿病天地,2019,16(6):33-34.
477. 刘伟,秦美乐,焦秋芬. 中西医结合治疗冠心病心绞痛的临床疗效观察[J]. 特别健康,2021(18):135.
478. 王丽哲,刘延东,王萌嘉,等. 中西医结合治疗冠心病心绞痛的临床疗效观察[J]. 中国卫生标准管理,2021,12(3):116-118.
479. 姜志阳,刘克岩. 中西医结合治疗冠心病心绞痛的临床疗效观察[J]. 世界最新医学信息文摘（连续型电子期刊）,2017,17(10):148,151.
480. 俞琦. 中西医结合治疗冠心病心绞痛的临床效果[J]. 糖尿病天地,2020,17(9):18.
481. 张志强,范立华,张志国. 中西医结合治疗冠心病心绞痛的临床效果分析[J]. 实用中西医结合临床,2018,18(2):59-60.
482. 樊丽. 中西医结合治疗冠心病心绞痛的临床研究[J]. 中国保健营养,2018,28(23):72-73.
483. 胡黎文,方元娥. 中西医结合治疗冠心病心绞痛观察与护理[J]. 实用中医药杂志,2018,34(4):446-447.
484. 陈博. 中西医结合治疗冠心病心绞痛患者的临床疗效[J]. 临床研究,2017,25(1):28-29.
485. 李朝虹,薛春香,郑玉春,李凤霞,张海军,赵磊. 中西医结合治疗冠心病心绞痛疗效观察[J]. 科学养生,2021 (3):211.
486. 章琪.中西医结合治疗冠心病心绞痛疗效观察[J].新中医,2017,49(12):24-26.
487. 刘媛琴. 中西医结合治疗冠心病心绞痛临床观察[J]. 中国中医药现代远程教育,2021,19(13):134-136.
488. 甘一立. 中西医结合治疗冠心病心绞痛临床观察[J]. 实用中医药杂志,2020,36(5):638-639.
489. 卞水朋. 中西医结合治疗冠心病心绞痛心血瘀阻证68例疗效观察[J]. 临床研究,2017,25(1):27-28.
490. 王生娟. 中西医结合治疗冠心病PCI术后胸痛的影响分析[J]. 青海医药杂志,2018,48(4):62-63.
491. 蔡昀思,胡珊,陶璐. 中西医结合治疗缓解期冠心病心绞痛[J]. 养生保健指南,2017(10):318.
492. 周冰. 中西医结合治疗急性冠脉综合征PCI术后心绞痛53例临床观察[J]. 中国民族民间医药,2019,28(11):71-73.
493. 丁金明. 中西医结合治疗老年不稳定型心绞痛63例的临床观察[J]. 世界最新医学信息文摘（连续型电子期刊）,2019,19(62):210-211.
494. 廖思晓,黄艳. 中西医结合治疗老年冠心病不稳定型心绞痛的疗效观察[J]. 内蒙古中医药,2017,36(23):72-73.
495. 王艳丽,关金玲,黄芳. 中西医结合治疗老年冠心病心绞痛82例疗效观察研究[J]. 世界最新医学信息文摘,2021,21(28):224-225.
496. 赵传辉. 中西医结合治疗老年冠心病心绞痛疗效分析[J]. 中西医结合心血管病电子杂志,2018,6(22):175,179.
497. 金欢亮. 中西医结合治疗弥漫性冠脉病变心绞痛的疗效观察[J]. 健康必读,2021(22):119.
498. 屈历涛. 中西医结合治疗气虚痰瘀型稳定型心绞痛疗效观察[J]. 山西中医,2017,33(9):19-20.
499. 李雪姣. 中西医结合治疗气虚血瘀型心绞痛的临床观察[J]. 中国城乡企业卫生,2021,36(7):138-140.
500. 潘玉霞. 中西医结合治疗气滞血瘀型不稳定心绞痛的效果[J]. 中国城乡企业卫生,2022,37(2):162-163.
501. 李国诗,潘枚霞,朱东杰. 中西医结合治疗气滞血瘀型冠心病不稳定性心绞痛33例临床观察[J]. 中国民族民间医药,2019,28(17):109-110,118.
502. 马峥尧. 中西医结合治疗痰浊闭阻型胸痹心痛患者的临床疗效[J]. 中国药物经济学,2020,15(10):67-69.
503. 金惠玲. 中西医结合治疗微血管心绞痛(心脉瘀阻型)的临床观察[J]. 中国民间疗法,2019,27(17):68-69.
504. 秦玲,王利红,周振华. 中西医结合治疗稳定型心绞痛的临床研究[J]. 中国继续医学教育,2017,9(30):123-125.
505. 吴旻,苏伟. 中西医结合治疗稳定型心绞痛气虚血瘀证临床研究[J]. 医学食疗与健康,2020,18(10):26-27.
506. 冯广超. 中西医结合治疗稳定性心绞痛并焦虑症的临床疗效分析[J]. 智慧健康,2020,6(2):105-107.
507. 李永浩. 中西医结合治疗稳定性心绞痛的临床效果观察[J]. 疾病监测与控制,2017,11(6):471-473.
508. 甄耀辉. 中西医结合治疗心绞痛40例临床观察[J]. 中国民族民间医药,2017,26(4):92-94.
509. 唐郡. 中西医结合治疗心绞痛伴植物神经功能紊乱的疗效观察[J]. 世界最新医学信息文摘（连续型电子期刊）,2020,20(13):20-21.
510. 尹金花. 中西医结合治疗心绞痛的临床研究[J]. 饮食保健,2019,6(9):86-87.
511. 毛美安. 中西医结合治疗胸痹心痛心血瘀阻证103例疗效观察[J]. 湖南中医杂志,2017,33(5):52-54.
512. 赵海峰. 中西医结合治疗血瘀型心绞痛患者的效果[J]. 中国民康医学,2021,33(18):91-93.
513. 李晓云,崔国方. 中西医结合治疗与单纯西药治疗冠心病心绞痛的效果差异分析[J]. 中西医结合心血管病电子杂志,2018,6(34):175-176.
514. 赵鑫. 中西医联合治疗对心绞痛合并糖尿病患者生活质量的影响[J]. 中国初级卫生保健,2020,34(7):90-92.
515. 王刚华. 中西医联合治疗冠心病心绞痛的临床效果[J]. 养生保健指南,2017(25):36.
516. 陈仙萍. 中西医联合治疗冠心病心绞痛患者的临床观察[J]. 光明中医,2018,33(16):2425-2427.
517. 贾晶,钟君华. 中西医联合治疗冠心病心绞痛患者的临床效果分析[J]. 右江医学,2019,47(2):95-97.
518. 李青霞. 中西医联合治疗冠心病心绞痛患者对心功能及睡眠指标的改善效果分析[J]. 世界睡眠医学杂志,2020,7(4):600-602.
519. 王卫国,王慧玲. 中西医联合治疗气虚血瘀型心绞痛的临床效果分析[J]. 河南医学研究,2017,26(20):3756-3757.
520. 张宝楠,刘春愉,李雁,等. 中西医临床护理路径在胸痹瘀血痹阻患者中的应用效果[J]. 光明中医,2020,35(14):2234-2236.
521. 薛刚,闻婷,胡刚,等. 中药复方穴位贴敷治疗冠心病心绞痛24例临床观察[J]. 湖南中医杂志,2018,34(10):49-51.
522. 杨丽霞. 中药复方制剂治疗冠心病心绞痛(热瘀痰阻证)的临床疗效分析[J]. 中国实用医药,2018,13(30):126-127.
523. 刘波. 中药复方治疗气虚血瘀型冠心病心绞痛的效果分析[J]. 健康大视野,2020(24):106-107.
524. 邵长信,房艳玲. 中药降脂通脉汤联合瑞舒伐他汀对冠心病心绞痛合并高血脂症患者疗效分析[J]. 医药前沿,2019,9(16):111.
525. 李玉梅,赵菲,李俊毅. 中药热奄包外敷至阳、内关穴治疗冠心病心绞痛的临床观察与研究[J]. 饮食保健,2020(49):106-107.
526. 陈兰,张小英,刘燕华,等. 中药热奄包在冠心病心绞痛治疗中的应用[J]. 光明中医,2021,36(18):3139-3141.
527. 李达,闫建玲,袁长玲,等. 中药热奄包治疗稳定型心绞痛(阳虚脉阻证)临床疗效观察[J]. 山西中医学院学报,2019,20(6):458-459.
528. 于游. 中药胸痹汤1号联合中医辨证护理干预冠心病心绞痛58例[J]. 中国中医药现代远程教育,2020,18(19):139-141.
529. 蒙雅群. 中药穴位贴敷对胸痹心痛病(心血瘀阻)症状缓解的效果探析[J]. 临床医学研究与实践,2017,2(34):105-106.
530. 余翠琴,马芳. 中药益气通脉汤结合西药在冠心病合并心绞痛患者中进行治疗的临床疗效观察[J]. 东方药膳,2020(4):247.
531. 郑斯玉,王宇,王娟,等. 中药益气养阴活血解毒汤对糖尿病合并冠心病心绞痛病人的影响观察[J]. 饮食保健,2018,5(9):104-105.
532. 李譞. 中药自拟方对不稳定型心绞痛疗效分析[J]. 大家健康（中旬版）,2017,11(10):32-33.
533. 张成弛. 中医辨证治疗痰瘀互结证之胸痹患者的临床疗效[J]. 中国药物经济学,2021,16(10):79-82,86.
534. 曾博斯,李亚轩,李健,等. 中医定向透药联合耳穴压豆治疗冠心病心绞痛的效果观察[J]. 内科,2020,15(3):348-350.
535. 蔺隽婧,刘晴晴. 中医护理对冠心病心绞痛患者的影响研究[J]. 饮食保健,2020,7(6):182.
536. 张广珍. 中医护理方案在急诊科气滞血瘀胸痹心痛病中的应用效果[J]. 青海医药杂志,2018,48(11):49-51.
537. 程冰. 中医护理干预对变异型心绞痛患者的临床观察[J]. 中国民间疗法,2019,27(5):88-89.
538. 黄小春. 中医护理模式促进冠心病介入治疗患者康复的影响分析[J]. 中国实用医药,2018,13(2):151-152.
539. 汪永琴,秦琬玲,邓萍,等. 中医活血化淤论在治疗老年心血管疾病方面的应用研究[J]. 饮食保健,2018,5(2):114-115.
540. 夏禹. 中医活血化瘀法联合西药治疗稳定型心绞痛患者35例临床观察[J]. 药品评价,2022,19(4):238-241.
541. 刘长鑫. 中医活血化瘀汤与倍他乐克治疗冠心病心绞痛的价值分析[J]. 首都食品与医药,2019,26(4):38.
542. 李世阁. 中医汤药联合硝酸脂类药物治疗冠心病心绞痛的临床效果观察[J]. 中国卫生标准管理,2017,8(16):109-111.
543. 张道香,冯小燕,黄瑞聪,等. 中医体质辨识和干预对冠心病预后的临床研究[J]. 中国实用医药,2020,15(8):156-158.
544. 武俊英,邬永军. 中医药防治冠心病临床优势及有关疗效评价[J]. 中国保健营养,2017,27(9):379-380.
545. 刘涛. 中医药治疗冠状动脉粥样硬化性心脏病心绞痛的临床研究[J]. 健康大视野,2020(16):28.
546. 李文彬. 中医治疗冠心病心绞痛的临床分析[J]. 养生保健指南,2018(31):260.
547. 夏芮. 中医综合护理措施联合西药干预稳定性心绞痛发作期临床研究[J]. 新中医,2019,51(12):296-299.
548. 毛建芳,宋彦洁. 中医综合治疗对冠心病稳定性心绞痛患者的预后效果观察[J]. 中国保健营养,2018,28(29):93.
549. 徐进. 注射用丹参多酚酸盐治疗冠心病心绞痛的效果分析[J]. 医学理论与实践,2017,30(9):1292-1293.
550. 李晓微,石瑞君,杨欣欣. 自拟参术汤对气虚型冠心病心绞痛患者运动耐量的影响及药理学研究[J]. 光明中医,2020,35(19):3052-3054.
551. 边友锋,孙俊芝. 自拟化痰祛瘀通脉汤治疗冠心病不稳定型心绞痛临床观察[J]. 双足与保健,2018,27(10):187,189.
552. 马冲. 自拟化痰祛瘀通脉汤治疗冠心病不稳定型心绞痛临床价值分析[J]. 婚育与健康,2022,28(12):85-86.
553. 任海军. 自拟活血通络方治疗冠心病经皮冠状动脉 介入术术后心绞痛研究[J]. 健康大视野,2018(7):88,90.
554. 王桥福. 自拟通络舒心汤辨证治疗冠心病心绞痛 39 例讨论[J]. 自我保健,2020(24):251.
555. 陈宇. 自拟通心活血汤结合西药治疗冠心病心绞痛的临床疗效观察[J]. 中国疗养医学,2017,26(1):80-81.
556. 金鑫,马晓妍,王若楠. 自拟温阳通痹汤联合尼可地尔治疗寒凝心脉型胸痹临床观察[J]. 光明中医,2020,35(11):1704-1705.
557. 张永魁. 自拟益气通脉汤联合曲美他嗪治疗冠心病心绞痛[J]. 牡丹江医学院学报,2021,42(1):116-118.
558. 张永魁. 自拟益气通脉汤联合曲美他嗪治疗心绞痛的疗效评价[J]. 青岛医药卫生,2020,52(4):288-290.
559. 李春英,宗华. 自拟中草药汤剂辅助治疗冠心病心绞痛效果观察[J]. 中国乡村医药,2019,26(10):39-40.
560. 费凯,单晓晶,单金平,等. 子午流注联合穴位贴敷治疗冠心病心绞痛的临床研究[J]. 中国初级卫生保健,2020,34(3):91-94.
561. 王红涛. 子午流注纳甲法结合辨证取穴针刺治疗冠心病心绞痛的临床效果观察[J]. 现代诊断与治疗,2017,28(21):3945-3947.
562. 陈冬梅,魏明,张小彩,等. 子午流注音乐疗法治疗不稳定型心绞痛的临床疗效观察[J]. 世界最新医学信息文摘（连续型电子期刊）,2018,18(39):159,162.
563. 付进红.冠脉通片联合氯吡格雷治疗冠心病心绞痛的临床研究[J].现代药物与临床,2021,36(01):147-151.
564. 张方元.活血化瘀通脉丸治疗心脉瘀阻型胸痹临床研究[J].中西医结合心血管病电子杂志,2018,6(02):159-161.
565. 梁贵杰,李茂林,刘兰丽,等. 养心汤加减治疗冠心病不稳定型心绞痛(气虚血瘀型)患者的临床疗效观察[J]. 康颐,2022(7):128-130.
566. 李俊,郭亚萍. 参术冠心方对稳定型心绞痛患者血脂干预的效果及对心绞痛发作的影响[J]. 陕西中医,2018,39(2):181-183.
567. 李瑶. 复方丹参滴丸联合综合护理干预治疗冠状动脉粥样硬化性心脏病心绞痛临床评价[J]. 中国药业,2017,26(13):61-62.
568. 严萍. 隔附子饼灸配合药物治疗冠心病稳定型心绞痛疗效观察[J]. 上海针灸杂志,2017,36(3):276-278.
569. 王睿,胡海燕,庄艺. 院前急救揿针内关穴对不稳定性心绞痛硝酸甘油用量的影响[J]. 云南中医学院学报,2017,40(1):60-62.
570. 陈修文,陈军,张书富,徐金美,何立人.“清消通益方”联合西药治疗PCI术后痰瘀互结型胸痹30例临床研究[J].江苏中医药,2019,51(08):33-35.
571. 王美环. “益气活血，理气化痰”法治疗冠心病气虚痰瘀兼气滞证的临床观察[D].辽宁中医药大学,2021.
572. 夏青. 参丹饮对稳定性心绞痛（气虚血瘀证）患者的疲乏量表影响的临床观察[D].黑龙江中医药大学,2019.
573. 李爱勇,李享,尚菊菊,刘红旭,华春萱,戴梅,张大炜,周琦.参元益气活血胶囊对不稳定型心绞痛血瘀证病人择期PCI围术期心肌损伤的保护作用[J].中西医结合心脑血管病杂志,2017,15(05):519-522.
574. 吴文松. 盾叶冠心宁片治疗冠心病稳定型心绞痛的临床研究[D].南京中医药大学,2018.
575. 韩宁馨. 化痰活血宽胸方联合中药足浴治疗不稳定型心绞痛的临床回顾性研究[D].河北中医学院,2020.
576. 魏飞,柏茂树.芪参胶囊联合美托洛尔治疗冠心病心绞痛(气虚血瘀证)的疗效观察[J].中国中医急症,2019,28(07):1258-1261.
577. 金锦玉. 祛痰宣痹通络饮治疗稳定型心绞痛（痰瘀互阻证）的临床疗效观察[D].黑龙江中医药大学,2020.
578. 赵伟征. 散结通络汤治疗冠心病稳定型心绞痛（痰浊闭阻证）的临床疗效观察[D].黑龙江中医药大学,2020.
579. 奚玉鑫. 升清降浊法治疗冠心病稳定型心绞痛（痰阻心脉证）的临床疗效观察[D].黑龙江中医药大学,2018.
580. 郭悦婷. 四逆散合补阳还五汤加减治疗冠心病心绞痛（气滞血瘀证）的临床观察[D].黑龙江中医药大学,2020.
581. 赵亮. 稳斑汤联合体外反搏治疗不稳定型心绞痛临床疗效及对内皮功能影响[D].辽宁中医药大学,2018.
582. 王殿明. 香附旋复花汤加减治疗冠心病稳定型心绞痛（痰浊闭阻证）的临床观察[D].黑龙江中医药大学,2021.
583. 曹燕娣,车瑾.薤白宣痹通络方联合他汀类药物治疗不稳定性心绞痛对患者Th1/Th2细胞因子平衡的影响[J].四川中医,2021,39(04):96-99.
584. 李洪伟. 心元胶囊对稳定型心绞痛（心血瘀阻型）患者血脂影响的临床研究[D].黑龙江中医药大学,2018.
585. 陈斌. 养心汤加减对不稳定型心绞痛（气虚血瘀）患者中医证候疗效及LTB4的影响[D].黑龙江中医药大学,2019.
586. 陈瑶. 养心汤加减治疗冠心病稳定型心绞痛（气虚血瘀型）的临床观察[D].黑龙江中医药大学,2020.
587. 李拯宇. 益气活血方治疗不稳定型心绞痛（气虚血瘀型）PCI术后回顾性分析[D].北京中医药大学,2020.
588. 黄恺悦. 益心通痹汤对气虚血瘀、痰浊阻滞型非阻塞性冠状动脉微血管疾病患者疗效的临床研究[D].成都中医药大学,2018.
589. 王秀玲. 薏苡附子散加减治疗冠心病不稳定型心绞痛（阳气虚衰型）的临床观察[D].黑龙江中医药大学,2018.
590. 吴萍. 枳实薤白桂枝汤合小陷胸汤治疗痰热内阻型稳定型心绞痛的临床观察[D].黑龙江中医药大学,2020.
591. 李静,宋莉丽.中西医结合治疗糖尿病合并冠心病心绞痛的临床观察[J].中华中医药学刊,2019,37(02):505-508.
592. 王毅然,于玲,周亚滨.中药穴位贴敷联合养心汤治疗慢性稳定型心绞痛[J].吉林中医药,2019,39(10):1377-1380.
593. 谢先余,花继平,任印新,丁丽,孙慧,蒋志坤.注射用益气复脉（冻干）对不稳定型心绞痛（气阴两虚证）患者中医症候的疗效观察[J].药物评价研究,2021,44(11):2481-2486.
594. 李旭东,苏志强,黄德城,杨云,黄有胜,代珊荣.CYP2C19基因检测在活血化瘀中成药联合抗血小板西药治疗稳定型心绞痛中的临床意义[J].中西医结合心脑血管病杂志,2020,18(17):2825-2828.
595. Cai X, Du J, Li L, Zhang P, Zhou H, Tan X, Li Y, Yu C. Clinical metabolomics analysis of therapeutic mechanism of Tongmai Yangxin Pill on stable angina. J Chromatogr B Analyt Technol Biomed Life Sci. 2018 Nov 15;1100-1101:106-112.
596. 张学会. 温胆汤合丹参饮联合西药治疗痰瘀互结型稳定性心绞痛的临床观察[D].华北理工大学,2021.
597. 倪晖君,李威,任建素.心胃同治法治疗痰阻心脉型冠心病心绞痛临床研究[J].中国医药,2022,17(07):1065-1069.
598. 唐婉斯. 熊氏十味温胆汤治疗冠心病不稳定性心绞痛气虚痰瘀证的临床疗效观察[D].湖南中医药大学,2020.
599. 卢西. 血脂康联合依折麦布对他汀不耐受稳定型冠心病（痰瘀证）疗效观察[D].华北理工大学,2020.
600. 陈瑶,王艳,薛强,等. 基于"心肝同治法"探讨心痛舒治疗老年冠心病心绞痛的效果[J]. 中国老年学杂志,2022,42(3):519-522.
601. 迟伟峰,宋彦洁,迟京秀,卢英红.补肾活血汤治疗肾虚血瘀型劳累性心绞痛患者的临床观察[J].中药药理与临床,2019,35(03):168-171.
602. Sun MY, Miao Y, Jin M, Dong YR, Liu SR, Wang ML, Gao R. Effect and Safety of Guanxinning Tablet () for Stable Angina Pectoris Patients with Xin (Heart)-Blood Stagnation Syndrome: A Randomized, Multicenter, Placebo-Controlled Trial. Chin J Integr Med. 2019 Sep;25(9):684-690.
603. 穆丽婷,赵英强,张清.参芍胶囊为对照加用活血药对稳定型劳累性心绞痛的证候疗效评价[J].中国中医基础医学杂志,2018,24(06):789-791+827.
604. 朱岩,陈民.基于中医辨证论治的心脏康复联合参芪五味子汤对老年冠心病心绞痛患者生存质量的影响[J].辽宁中医杂志,2021,48(08):148-151.
605. 高改地,何庆勇,陈新宇,钱海凌,杨蕊琳,刘超峰,孙旭,李军.理气活血滴丸治疗冠心病心绞痛随机双盲Ⅱ期临床研究[J].中国循证心血管医学杂志,2018,10(09):1074-1078.
606. 高改地,李军,何庆勇,陈新宇,钱海凌,杨蕊琳,王莎萍,孙旭.理气活血滴丸治疗冠心病稳定型心绞痛的有效性和安全性研究[J].中国循证心血管医学杂志,2020,12(01):62-66.
607. 王振兴. 麝香通心滴丸治疗痰热瘀阻型冠心病稳定型心绞痛的临床疗效评价[D].天津中医药大学,2021.
608. 姜广. 十味温胆汤加减治疗气虚痰瘀型冠心病SAP的临床研究[D].云南中医学院,2018.
609. 戴玉,李艳,张磊.四花穴、心俞隔药饼灸治疗气虚血瘀型冠心病心绞痛随机对照研究[J].吉林中医药,2018,38(02):227-229.
610. 崔文硕. 太阴调胃汤合熊胆散治疗冠心病心绞痛的实验与临床研究[D].延边大学,2018.
611. 李同达,李冬梅,段文慧,等. 温心方颗粒治疗慢性稳定性心绞痛阳虚痰瘀证临床研究[J]. 国际中医中药杂志,2017,39(5):397-401.
612. 刘汶阳. 稳心4号方加减治疗冠心病稳定性心绞痛痰瘀互结证的临床研究[D]. 吉林:长春中医药大学,2018.
613. 张恒. 血府逐瘀合剂治疗稳定型心绞痛（气滞血瘀证）的临床研究[D]. 湖北:湖北中医药大学,2018.
614. 高建伟,高学敏,邹婷,赵天蒙,王东华,吴宗贵,任长杰,王兴,耿乃志,赵明君,梁秋明,冯星,杨柏松,史俊玲,华琦.心灵丸治疗稳定性劳力性心绞痛:随机、双盲、安慰剂平行对照、多中心临床试验[J].中国中药杂志,2018,43(06):1268-1275.
615. 李永春,郭丽. 针刺从宗气论治稳定型心绞痛气虚血瘀证的临床观察[J]. 中国中医急症,2017,26(2):303-305.
616. 崔杰,曹征,沈娟,刘洋,匡武,李岩,逯金金,张婧倩,吴旸.注射用血塞通治疗冠心病稳定性心绞痛临床观察[J].中华中医药学刊,2020,38(02):47-49.
617. 王兴. 孙光荣学术思想和临床经验总结及孙氏胸痹汤治疗稳定性心绞痛气虚痰瘀证的临床研究[D].北京中医药大学,2017.
618. 秦鹏. 复方丹参滴丸对胸痹（冠心病心绞痛）气滞血瘀证患者中心动脉压的影响[D].南京中医药大学,2017.
619. 许国猛,施钰琳.“冬病夏治”温阳活血膏治疗心肾阳虚证冠心病心绞痛的疗效和安全性[J].世界中医药,2018,13(07):1617-1620.
620. 刘蕾. 麝香保心丸治疗气滞血瘀型稳定性心绞痛的临床研究[D].北京中医药大学,2020.
621. Huang S, Li L, Liu J, Li X, Shi Q, Li Y, Liu Y, Li M, Ma L, Ning L, Liao X, Ying X, Cai W, Yang F, Wang T, Guo R, Ma W, Chen W, Chen J, Sun X. The Preventive Value of Acupoint Sensitization for Patients with Stable Angina Pectoris: A Randomized, Double-Blind, Positive-Controlled, Multicentre Trial. Evid Based Complement Alternat Med. 2021 Nov 2;2021:7228033.
622. Yang QN, Bai RN, Dong GJ, Ge CJ, Zhou JM, Huang L, He Y, Wang J, Ren AH, Huang ZQ, Zhu GL, Lu S, Xiong SQ, Xian SX, Zhu ZJ, Shi DZ, Lu SZ, Li LZ, Chen KJ. Effect of Kuanxiong Aerosol () on Patients with Angina Pectoris: A Non-inferiority Multi-center Randomized Controlled Trial. Chin J Integr Med. 2018 May;24(5):336-342.
623. Dastani M, Bigdelu L, Hoseinzadeh M, Rahimi HR, Karimani A, Hooshang Mohammadpour A, Salari M. The effects of curcumin on the prevention of atrial and ventricular arrhythmias and heart failure in patients with unstable angina: A randomized clinical trial. Avicenna J Phytomed. 2019 Jan-Feb;9(1):1-9.
624. Ma Y, Lv W, Gu Y, Yu S. 1-Deoxynojirimycin in Mulberry (Morus indica L.) Leaves Ameliorates Stable Angina Pectoris in Patients With Coronary Heart Disease by Improving Antioxidant and Anti-inflammatory Capacities. Front Pharmacol. 2019 May 21;10:569.
625. DeVon HA, Uwizeye G, Cai HY, Shroff AR, Briller JE, Ardati A, Hoppensteadt D, Rountree L, Schlaeger JM. Feasibility and preliminary efficacy of acupuncture for angina in an underserved diverse population. Acupunct Med. 2022 Apr;40(2):152-159.
626. Guo J, Dai S, Ding Y, He H, Zhang H, Dan W, Qin K, Wang H, Li A, Meng P, Li S, He Q. A randomized controlled trial for gualou danshen granules in the treatment of unstable angina pectoris patients with phlegm-blood stasis syndrome. Medicine (Baltimore). 2020 Aug 14;99(33):e21593.
627. Wang K, Cai JJ, Wu Y, Wang Y, Liu LL, Shi L, Wang X. Prospective randomized controlled trial study of Luofengning granule in the treatment of unstable angina. Medicine (Baltimore). 2020 May;99(20):e20025.
628. Zhao JN, Zhang Y, Lan X, Chen Y, Li J, Zhang P, Wu LQ, Jia ST, Liu Y, Xu FQ. Efficacy and safety of Xinnaoning capsule in treating chronic stable angina (qi stagnation and blood stasis syndrome): Study protocol for a multicenter, randomized, double-blind, placebo-controlled trial. Medicine (Baltimore). 2019 Aug;98(31):e16539.
629. Wang Y, Xu Y, Zhang L, Huang S, Dou L, Yang J, Fu W, Zhou P, Wan H. Comparison of Buyang Huanwu granules and Naoxintong capsules in the treatment of stable angina pectoris: rationale and design of a randomized, blinded, multicentre clinical trial. Trials. 2022 Jan 21;23(1):65.
630. Gao JW, Gao XM, Zou T, Zhao TM, Wang DH, Wu ZG, Ren CJ, Wang X, Geng NZ, Zhao MJ, Liang QM, Feng X, Yang BS, Shi JL, Hua Q. [Effect of Xinling Wan in treatment of stable angina pectoris: a randomized, double-blinded, placebo parallel-controlled, multicenter trial]. Zhongguo Zhong Yao Za Zhi. 2018 Mar;43(6):1268-1275.
631. 徐亦男. 心肌桥患者发生心绞痛与肌桥指数的关系及天香丹的干预研究[D].新疆医科大学,2019.

# Supplementary file 6 Specific information of Specific information of Table 2 and Figure 3

## 1. Specific information of “Others” in TCM patterns of AP in Figure 3 (n=62)

| **Pattern** | **n (%)** |
| --- | --- |
| Excess cold pattern | 7 (11.29) |
| Qi stagnation and intertwined phlegm and blood stasis pattern | 6 (9.68) |
| Blood stasis transformed heat pattern | 6 (9.68) |
| Yang deficiency of the heart and kidney pattern | 5 (8.06) |
| Cold induced blood stasis pattern | 5 (8.06) |
| Kidney deficiency and blood stasis pattern | 5 (8.06) |
| Disharmony between the heart and kidney pattern | 5 (8.06) |
| Yang deficiency pattern | 4 (6.45) |
| Spleen deficiency with phlegm dampness pattern | 3 (4.84) |
| Body constitution | 2 (3.23) |
| Phlegm qi stagnation pattern | 2 (3.23) |
| Deficiency of the spleen and kidney pattern | 1 (1.61) |
| Liver hyperactivity with spleen deficiency and heart yang deficiency pattern | 1 (1.61) |
| Damp heat pattern | 1 (1.61) |
| Yang deficiency of the spleen and kidney pattern | 1 (1.61) |
| Upward flaming of heart fire pattern | 1 (1.61) |
| Luofeng Neidong pattern | 1 (1.61) |
| Yin deficiency and qi stagnation pattern | 1 (1.61) |
| Excess heat in the gallbladder pattern | 1 (1.61) |
| Liver hyperactivity with spleen deficiency and intertwined phlegm and blood stasis pattern | 1 (1.61) |
| Liver qi stagnation transforming into heat pattern | 1 (1.61) |
| Heart qi deficiency pattern | 1 (1.61) |
| Yin deficiency pattern | 1 (1.61) |

**2.** **Specific information of “Others” in classification of AP in Table 2 (n=111)**

| **Types of AP** | **n (%)** |
| --- | --- |
| Not specified | 51 (45.95) |
| Post-PCI | 40 (36.04) |
| Stable and Unstable | 8 (7.21) |
| Microvascular | 6 (5.41) |
| Refractory | 3 (2.70) |
| After myocardial infarction | 2 (1.80) |
| Stable and Microvascular | 1 (0.90) |

**3. Specific information of 3 assigned groups** **among included articles**

| **Western Medicine** | **Single Chinese Herbal Medicine** | **Integrated Traditional Chinese and Western Medicine** |
| --- | --- | --- |
| Aspirin enteric-coated tablets | Salvia Miltiorrhiza Depside Salt (SMDS) | SMDS combined with Aspirin |
| Nitroglycerin and other conventional WM pharmacological therapy for AP (including β-blockers, aspirin, statins, and angiotensin-converting enzyme inhibitors) | Self-made Yiqi Huoxue decoction | Self-made Yiqi Huoxue decoction combined with Nitroglycerin and other conventional WM pharmacological therapy |
| **Western Medicine** | **Western Medicine combined with TCM placebo** | **Integrated Traditional Chinese and Western Medicine** |
| Conventional WM pharmacological therapy for AP (including β-blockers, aspirin, statins, and angiotensin-converting enzyme inhibitors) | Placebo of acupoint application medicine combined with Conventional WM pharmacological therapy for AP | Wenyang Huoxue formula for acupoint application combined with Conventional WM pharmacological therapy for AP |
| **Western Medicine** | **Integrated Traditional Chinese and Western Medicine** | **Integrated Traditional Chinese and Western Medicine (different treatment method of TCM)** |
| Conventional WM pharmacological therapy for AP (including β-blockers, aspirin, statins, and angiotensin-converting enzyme inhibitors) | Conventional WM pharmacological therapy for AP combined with Xuefu Zhuyu decoction (twice a day, no restriction to time) | Conventional WM pharmacological therapy for AP combined with Xuefu Zhuyu decoction (twice a day, respectively in 11 am and 7 pm) |
| Conventional WM pharmacological therapy for AP (including β-blockers, aspirin, statins, and angiotensin-converting enzyme inhibitors) | Conventional WM pharmacological therapy for AP combined with Shuangshen Ningxin formula (Medicinal granules) | Conventional WM pharmacological therapy for AP combined with Shuangshen Ningxin decoction |
| Conventional WM pharmacological therapy for AP (including β-blockers, aspirin, statins, and angiotensin-converting enzyme inhibitors) | Conventional WM pharmacological therapy for AP combined with Xuefu Zhuyu decoction | Conventional WM pharmacological therapy for AP combined with Xuefu Zhuyu decoction plus rhubarb processed by yellow rice wine |
| **Western Medicine** | **Combined Western Medicine (different form)** | **Integrated Traditional Chinese and Western Medicine** |
| Conventional WM pharmacological therapy for AP (including β-blockers, aspirin, statins, and angiotensin-converting enzyme inhibitors) | Conventional WM pharmacological therapy for AP combined with External Counter pulsation treatment | Conventional WM pharmacological therapy for AP combined with External Counter pulsation treatment and Hedan capsule |

**4. Specific information of 4 assigned groups among included articles**

| All groups are ITCWM design, which receive conventional WM pharmacological therapy for AP (including β-blockers, aspirin, statins, and angiotensin-converting enzyme inhibitors) combined with different TCM therapy | | | |
| --- | --- | --- | --- |
| Disease-affected meridian acupuncture | Non-affected meridian acupuncture | Sham-acupuncture | Wait-list |
| Selecting acupoint along meridian acupuncture | Selecting acupoint from another meridian acupuncture | Sham acupoint acupuncture | Wait-list |
| Compound salvia miltiorrhiza medicinal granules | Compound salvia miltiorrhiza capsule | Compound salvia miltiorrhiza tablets | Compound salvia miltiorrhiza condensed pill |
| Western Medicine | Combined Western Medicine (different medicine) | Integrated Traditional Chinese and Western Medicine | Integrated Traditional Chinese and Western Medicine (different medicine) |
| Aspirin | Aspirin combined with Simvastatin | Aspirin combined with Huangqi Guizhi Wuwu decoction | Aspirin combined with Simvastatin and Huangqi Guizhi Wuwu decoction |
| Western Medicine | Integrated Traditional Chinese and Western Medicine | Integrated Traditional Chinese and Western Medicine (different medicine) | Integrated Traditional Chinese and Western Medicine (different medicine) |
| Conventional WM pharmacological therapy (Enteric aspirin, Clopidogrel sulfate tablets, Atorvastatin calcium tablets, Enoxaparin, Betaloc sustained-release tablets, Diltiazem) | Conventional WM pharmacological therapy combined with Xinmaitong powder for acupoint application | Conventional WM pharmacological therapy combined with Qishen Yiqi condensed pill | Conventional WM pharmacological therapy combined with Qishen Yiqi condensed pill and Xinmaitong powder for acupoint application |
| Conventional WM pharmacological therapy for AP (including β-blockers, aspirin, statins, and angiotensin-converting enzyme inhibitors) | conventional WM pharmacological therapy for AP combined with Juhong tablets | conventional WM pharmacological therapy for AP combined with Tongxinluo capsule | conventional WM pharmacological therapy for AP combined with Tongxinluo capsule and Juhong tablets |

**5. Specific information of complex TCM and external TCM therapies**

| **Complex TCM intervention (n=25)** | **n (%)** |
| --- | --- |
| Acupuncture combined with Oral Chinese medicine | 8 (32) |
| Acupoint application combined with Oral Chinese medicine | 5 (20) |
| Ear points therapy combined with Oral Chinese medicine | 3 (12) |
| Moxibustion combined with Oral Chinese medicine | 2 (8) |
| Foot bath combined with Oral Chinese medicine | 1 (4) |
| Acupoint application and acupuncture combined with Oral Chinese medicine | 1 (4) |
| Acupoint application and Tuina combined with Oral Chinese medicine | 1 (4) |
| Guasha therapy combined with Oral Chinese medicine | 1 (4) |
| Gongfa and Tuina combined with Oral Chinese medicine | 1 (4) |
| Tuina combined with Oral Chinese medicine | 1 (4) |
| Gongfa combined with Five element music therapy | 1 (4) |
| **External therapy (n=27)** | **n (%)** |
| Acupuncture | 10 (37.04) |
| Acupoint application | 9 (33.33) |
| Gongfa | 3 (11.11) |
| Moxibustion | 1 (3.70) |
| Tuina | 1 (3.70) |
| Psychological treatment of TCM | 1 (3.70) |
| Five element music therapy | 1 (3.70) |
| Ear points therapy | 1 (3.70) |

**Supplementary file 7 Details of the CONSORT and ITCWM-specific items**

| **Section/topic** | **Item number and description** | **Fully reported,**  **n (%)** | | **Partially reported,**  **n (%)** | | **Not reported,**  **n (%)** | **^#^Agreement** |  |  |
| --- | --- | --- | --- | --- | --- | --- | --- | --- | --- |
| Title and abstract | 1a. Identification as a randomized trial in the title | 8 (1.77) | | - | | 443 (98.23) | 1 |  |  |
|  | 1b. Structured summary of trial design, methods, results, and conclusions | See Table 4 | | | | | |  | See Abstract |
|  | *Q1. Whether the feature of ITCWM was presented in Title?* | 71 (15.74) | | - | | 380 (84.26) | 0.84 |  |  |
|  | *Q6.* *Whether the feature or design of ITCWM study were reflected in Keywords?* | 58 (12.86) | | 390 (86.47) | | 3 (0.67) | 1 |  |  |
| Introduction |  |  | |  | |  |  |  |  |
| Background | 2a. Scientific background and explanation of rationale | 450 (99.78) | | - | | 1 (0.22) | 1 |  |  |
|  | *Q7. Whether the reason/rationale about ITCWM intervention for the study design was reported in the Background?* | 183 (40.58) | | 102 (22.62) | | 166 (36.81) | 0.87 |  |  |
| Objectives | 2b. Specific objectives or hypotheses | 362 (80.27) | | - | | 89 (19.73) | 0.90 |  |  |
|  | *Q8. Whether the objectives or hypotheses were focused on the ITCWM interventions in the Background?* | 95 (21.06) | | - | | 356 (78.94) | 0.81 |  |  |
| Methods |  |  | |  | |  |  |  |  |
| Trial design | 3a. Description of trial design (such as parallel, factorial) including allocation ratio | 27 (5.99) | | 102 (22.62) | | 322 (71.40) | 0.60 |  |  |
|  | 3b. Important changes to methods after trial commencement (such as eligibility criteria), with reasons | 13 (2.88) | | - | | 438 (97.12) | 0.91 |  |  |
| Participants | 4a. Eligibility criteria for participants | 443 (98.23) | | 7 (1.55) | | 1 (0.22) | 1 |  |  |
|  | *Q9. Whether the eligibility criteria of participants included both Chinese and western medical diagnosis in Methods?* | 395 (87.58) | | 7 (1.55) | | 49 (10.86) | 0.65 |  |  |
|  | *Q10. Whether the specific information of disease (e.g., classification of disease, treatment points, stages of diseases) of the ITCWM was reported in Methods?* | 400 (88.69) | | - | | 51 (11.31) | 0.68 |  |  |
|  | 4b. Settings and locations where the data were collected | 416 (92.24) | | 29 (6.43) | | 6 (1.33) | 0.80 |  |  |
| Interventions | 5. The interventions for each group with sufficient details to allow replication, including how and when they were actually administered | 330 (73.17) | | 118 (26.16) | | 3 (0.67) | 0.84 |  |  |
|  | *Q11. Whether the specific type/way of integration of TCM and WM interventions (such as overlying, one-after-another, or add-on design) was reported in Methods?* | 442 (98.00) | | - | | 9 (2.00) | 0.92 |  |  |
|  | *Q12. In the ITCWM group, whether TCM intervention(s) was reported with sufficient details to allow replication, including how and when they were administered?* | 426 (94.46) | | - | | 25 (5.54) | 0.64 |  |  |
|  | *Q13. In the ITCWM group, whether WM intervention(s) was reported with sufficient details to allow replication, including how and when they were administered?* | 350 (77.61) | | - | | 101 (22.39) | 0.91 |  |  |
|  | *Q14. In the control group, whether sufficient details were reported to allow replication, especially for the placebo?* | 360 (79.82) | | - | | 91 (20.18) | 0.77 |  |  |
| Outcomes | 6a. Completely defined pre-specified primary and secondary outcome measures, including how and when they were assessed | 24 (5.32) | | 345 (76.50) | | 82 (18.18) | 0.90 |  |  |
|  | 6b. Any changes to trial outcomes after the trial commenced, with reasons | - | | - | | 451 (100) | 1 |  |  |
|  | *Q15. Whether the outcome measures included both TCM and WM related endpoints in Methods?* | 390 (86.47) | | - | | 61 (13.53) | 0.79 |  |  |
| Sample size | 7a. How sample size was determined | 40 (8.87) | | 16 (3.55) | | 395 (87.58) | 0.96 |  |  |
|  | ^a^ 7 b. When applicable, explanation of any interim analyses and stopping guidelines | 93 (100) | | - | | - | 0.91 |  |  |
| Sequence generation | 8a. Method used to generate the random allocation sequence | 293 (64.97) | | 148 (32.82) | | 10 (2.22) | 0.83 |  |  |
|  | 8b. Type of randomization; details of any restriction (such as blocking and block size) | 36 (7.98) | | - | | 415 (92.02) | 0.73 |  |  |
| Allocation concealment mechanism | 9. Mechanism used to implement the random allocation sequence (such as sequentially numbered containers), describing any steps taken to conceal the sequence until interventions were assigned | 22 (4.88) | | - | | 429 (95.12) | 0.73 |  |  |
| Implementation | 10. Who generated the random allocation sequence, who enrolled participants, and who assigned participants to interventions | 2 (0.44) | | 7 (1.55) | | 442 (98.00) | 0.88 |  |  |
| Blinding | ^b^ 11a. If done, who was blinded after assignment to interventions (for example, participants, care providers, those assessing outcomes) and how | 6 (1.36) | | 28 (6.33) | | 408 (92.31) | 0.81 |  |  |
|  | *Q16. For the studies with open label, whether any reasons or explanations for such design was reported?* | 9 (100) | | - | |  | 0.74 |  |  |
|  | ^c^11b. If relevant, description of the similarity of interventions | 14 (63.64) | | - | | 8 (36.36) | 0.74 |  |  |
|  | *Q17. In the control group(s), did the placebo of WM invention(s) was included? If so, whether sufficient details were provided?* | - | | - | | 22 (100) | 1 |  |  |
|  | *Q18. In the control group(s), did the placebo of TCM invention(s) was included? If so, whether sufficient details were provided?* | 22 (100) | | - | | - | 1 |  |  |
| Statistical methods | 12a. Statistical methods used to compare groups for primary and secondary outcomes | 30 (6.65) | | 414 (91.80) | | 7 (1.55) | 0.93 |  |  |
|  | 12b. Methods for additional analyses, such as subgroup analyses and adjusted analyses | - | | - | | 451 (100) | 1 |  |  |
| Results |  |  | |  | |  |  |  |  |
| Participant flow (a diagram is strongly recommended) | 13a. For each group, the numbers of participants who were randomly assigned, received intended treatment, and were analysed for the primary outcome | 435 (96.45) | | 16 (3.55) | | - | 0.89 |  |  |
|  | 13b. For each group, losses and exclusions after randomization, together with reasons | 122 (27.05) | | 44 (9.76) | | 285 (63.19) | 0.86 |  |  |
| Recruitment | 14a. Dates defining the periods of recruitment and follow-up | 41 (9.09) | | 399 (88.47) | | 11 (2.44) | 0.63 |  |  |
|  | 14b. Why the trial ended or was stopped |  |  | | 451 (100) | | 1 |  |  |
| Baseline data | 15. A table showing baseline demographic and clinical characteristics for each group | 451 (100) | | - | | - | 0.93 |  |  |
|  | *Q19. In the section of Results, whether any information about the participants exposed to ITCWM treatment prior to recruitment was mentioned in the baseline data?* | - | | 8 (1.77) | | 443 (98.23) | 0.92 |  |  |
| Numbers analyzed | 16. For each group, number of participants (denominator) included in each analysis and whether the analysis was by original assigned groups | 436 (96.67) | | - | | 15 (3.33) | 0.87 |  |  |
| Outcomes and estimation | 17a. For each primary and secondary outcome, results for each group, and the estimated effect size and its precision (such as 95% confidence interval) | 3 (0.67) | | 438 (97.12) | | 10 (2.22) | 0.90 |  |  |
|  | 17b. For binary outcomes, presentation of both absolute and relative effect sizes is recommended | - | | - | | 451 (100) | 1 |  |  |
| Ancillary analyses | 18. Results of any other analyses performed, including subgroup analyses and adjusted analyses, distinguishing pre-specified from exploratory | - | | - | | 451 (100) | 1 |  |  |
| Harms | 19. All important harms or unintended effects in each group | 334 (74.06) | | 5 (1.11) | | 112 (24.83) | 0.84 |  |  |
| Discussion |  |  | |  | |  |  |  |  |
| Limitations | 20. Trial limitations, addressing sources of potential bias, imprecision, and, if relevant, multiplicity of analyses | 261 (57.87) | | - | | 190 (42.13) | 0.86 |  |  |
| Generalizability | 21. Generalizability (external validity, applicability) of the trial findings | 1 (0.22) | | - | | 450 (99.78) | 1 |  |  |
| Interpretation | 22. Interpretation consistent with results, balancing benefits and harms, and considering other relevant evidence | 451 (100) | | - | | - | 1 |  |  |
|  | *Q20. Whether interpretation and significance of studied ITCWM interventions for the disease was reported in Discussion?* | 66 (14.63) | | 261 (57.87) | | 124 (27.49) | 0.81 |  |  |
| Other information |  |  | |  | |  |  |  |  |
| Interests | *Q21. Whether any potential conflicts of interests were clearly reported?* | 16 (3.55) | | 65 (14.41) | | 370 (82.04) | 0.70 |  |  |
| Registration | 23. Registration number and name of trial registry | 7 (1.55) | | - | | 444 (98.45) | 1 |  |  |
| Protocol | 24. Where the full trial protocol can be accessed, if available | 1 (0.22) | | 1 (0.22) | | 449 (99.56) | 1 |  |  |
| Funding | 25. Sources of funding and other support (such as supply of drugs), role of funders | 1 (0.22) | | 123 (27.27) | | 327 (72.51) | 0.91 |  |  |

^a^ 358 studies was not calculated because it doesn’t include explanation of any interim analyses and stopping guidelines.

^b^ 9 studies was not calculated for open lable.

^c^ 429 studies was not calculated as it is not placebo design.

^#^Agreement: Calculation of Cohen’s kappa (k) coefficient for each item. The average value is 0.87.

**Supplementary file 8 Details of the CONSORT for abstract and ITCWM-specific items.**

| **Section/topic** | **Item number and description** | **Fully reported,**  **N (%)** | **Partially reported,**  **n (%)** | **Not reported,**  **N (%)** | **^#^Agreement** |
| --- | --- | --- | --- | --- | --- |
| Title | 1. Identification of the study as randomized | 8 (1.77) | - | 443 (98.23) | 1 |
| Authors | ^a^2. Contact details for the corresponding author | 123 (64.06) | 69 (35.94) | - | 1 |
| Trial design | 3. Description of the trial design (e.g., parallel, cluster, non-inferiority) | 69 (15.30) | - | 382 (84.70) | 0.62 |
| Methods |  |  |  |  |  |
| Participants | 4. Eligibility criteria for participants and the settings where the data were collected | 199 (44.12) | 252 (55.88) | - | 0.89 |
|  | *Q2. Whether the eligibility criteria of participants included both Chinese and western medical diagnosis in Methods of Abstract?* | 310 (68.74) | 141 (31.26) | - | 0.64 |
| Interventions | 5. Interventions intended for each group | 142 (31.49) | 296 (65.63) | 13 (2.88) | 0.69 |
| Objective | 6. Specific objective or hypothesis | 451 (100) | - | - | 1 |
|  | *Q3. Whether the study objectives or hypotheses were focused on the ITCWM interventions in Abstract?* | 93 (20.62) | - | 358 (79.38) | 0.93 |
| Outcome | 7. Clearly defined primary outcome for this report | 6 (1.33) | 427 (94.68) | 18 (3.99) | 0.84 |
|  | *Q4. Whether the outcome measures included both TCM and WM related endpoints in Abstract?* | 359 (79.60) | 72 (15.96) | 20 (4.43) | 0.85 |
| Randomization | 8. How participants were allocated to interventions | 161 (35.70) | 280 (62.08) | 10 (2.22) | 0.94 |
| Blinding (masking) | ^b^ 9. Whether or not participants, care givers, and those assessing the outcomes were blinded to group assignment | 2 (0.45) | 11 (2.45) | 436 (97.10) | 0.66 |
| Results |  |  |  |  |  |
| Numbers randomized | 10. Number of participants randomized to each group | 391 (86.70) | 2 (0.44) | 58 (12.86) | 0.81 |
| Recruitment | 11. Trial status | 4 (0.89) | 368 (81.60) | 79 (17.52) | 0.75 |
| Numbers analysed | 12. Number of participants analysed in each group | 37 (8.20) | 2 (0.44) | 412 (91.35) | 0.79 |
| Outcome | 13. For the primary outcome, a result for each group and the estimated effect size and its precision | - | 451 (100) | - | 0.91 |
| Harms | 14. Important adverse events or side effects | 148 (32.82) | 37 (8.20) | 266 (58.98) | 0.70 |
| Conclusions | 15. General interpretation of the results | 451 (100) | - | - | 1 |
|  | *Q5. Whether the interpretation of studied ITCWM interventions was reported in Conclusion of Abstract?* | 181 (40.13) | - | 270 (59.87) | 0.89 |
| Trial registration | 16. Registration number and name of trial register | 3 (0.67) | 4 (0.89) | 444 (98.45) | 1 |
| Funding | 17. Source of funding | 10 (2.22) | 114 (25.28) | 327 (72.51) | 0.91 |

^a^ 259 dessertations was not calculated because dessertations don’t include corresponding author.

^b^ 2 studies did not be calculated for open lable design.

^#^Agreement: Calculation of Cohen’s kappa (k) coefficient for each item. The average value is 0.85.

**Supplementary file 9 Overall reporting scores for included studies, by subgroup**

| Subgroup Mean (95% CI) | Type of publications (n) | | | | Total reports  (n=451) |
| --- | --- | --- | --- | --- | --- |
|  | Publications in Chinese  (n=442) | Publications in English  (n=9) | Dissertations  (n=259) | Journal publications  (n=192) |  |
| CONSORT items ^1^ | 27.65 (27.31-27.99) | 36 (28.22-43.78) | 29.36 (28.99-29.73) | 25.73 (25.13-26.34) | 27.82 (27.44-28.19) |
| CONSORT for AB items ^2^ | 14.15 (13.97-14.34) | 15.22 (11.50-18.94) | 14.13 (13.88-14.38) | 14.23 (13.93-14.54) | 14.17 (13.98-14.37) |
| ITCWM-specific items ^3^ | 21.11 (20.74-21.47) | 18.78 (13.33-24.22) | 21.42 (20.99-21.84) | 20.58 (19.93-21.23) | 21.06 (20.69-21.43) |

Abbreviation: CI, Confidence interval; AB, Abstract; ITCWM, integrated traditional Chinese medicine and western medicine

^1^ Full score of the CONSORT checklist is 72.

^2^ Full score of the CONSORT for AB checklist is 34.

^3^ Full score of the ITCWM-specific checklist is 42.
